# Virus Elimination from Naturally Infected Field Cultivars of Potato (*Solanum tuberosum*) by Transgenic RNA Interference

**DOI:** 10.3390/ijms23148020

**Published:** 2022-07-20

**Authors:** Alyona Alexandrova, Oxana Karpova, Ruslan Kryldakov, Victor Golyaev, Rufina Nargilova, Bulat Iskakov, Mikhail M. Pooggin

**Affiliations:** 1 M.A. Aitkhozhin Institute of Molecular Biology and Biochemistry, Almaty 050012, Kazakhstan; karpova480@gmail.com (O.K.); kryldakov@imbb.org.kz (R.K.); r.nargilova@imbb.org.kz (R.N.); iskakov.b@imbb.org.kz (B.I.); 2 Faculty of Biology and Biotechnology, Al-Farabi Kazakh National University, Almaty 050040, Kazakhstan; 3 PHIM Plant Health Institute, University of Montpellier, INRAE, CIRAD, IRD, Institute Agro, 34060 Montpellier, France; victor.golyaev@gmail.com

**Keywords:** *Solanum tuberosum*, potato virus S, potato virus M, carlavirus, virus elimination, RNA interference, siRNAs, transgenic plants, field trials, virus resistance

## Abstract

Tissue culture methods enable virus elimination from vegetatively propagated crop plants but cannot prevent new infections. Here we used a tissue culture transgenic approach for curing field cultivars of *Solanum tuberosum* through the stimulation of RNA interference (RNAi)-based antiviral defenses. Expression cassettes carrying inverted repeats of potato virus S (PVS, genus *Carlavirus*) movement or coat protein sequences were used for the transformation of potato cultivars naturally infected with PVS and/or a related carlavirus potato virus M (PVM), without or with potato virus Y (PVY, genus *Potyvirus*). A high proportion of transformants PCR-positive for transgenes were cured from both carlaviruses and PVY. After 3-year field trials, 22 transgenic lines representing seven cultivars remained free of any virus or became infected only with PVY. Vegetative progenies of the transgenic lines of cultivar Zeren (initially coinfected with PVS, PVM, and PVY), sampled after in vitro propagation or field trials, and other field cultivars accumulated transgene-derived 21, 22, and 24 nt small interfering (si)RNAs almost exclusively from the PVS inverted repeats. Additionally, some field progenies accumulated 21–22 nt siRNAs from the entire PVY genome, confirming PVY infection. Taken together, transgenic RNAi is effective for virus elimination from naturally infected potato cultivars and their sequence-specific immunization against new infections.

## 1. Introduction

Cultivated potato (*Solanum tuberosum*) is the world’s third most important food crop after wheat and rice. A large proportion of produced potato tubers are also used as livestock feed and as seed material [1]. Vegetative propagation of potatoes via seed tubers results in the accumulation and spread of pathogens and pests such as viruses, bacteria, fungi, and nematodes. Up to 75% yield losses can be caused by viral infection alone [2]. More than 40 different viruses are known to infect cultivated potatoes, with the most widespread being potato virus Y (PVY, genus *Potyvirus*, family *Potyviridae*), potato leaf roll virus (PLRV, genus *Polerovirus*, family *Luteoviridae*), potato virus X (PVX, genus *Potexvirus*, family *Alphaflexiviridae*), and potato viruses S and M (PVS and PVM, genus *Carlavirus*, family *Betaflexiviridae*) [3,4].

In Kazakhstan, the carlaviruses PVS and PVM are the most widespread and economically important potato viruses, followed by PVY and PVX [5]. PVS is most commonly detected in mixed infections with PVM and/or PVX, while PVM is prevalent both in single infections and coinfections with PVS, PVY, or both PVS and PVY [5]. PVS and PVM exhibit no leaf symptoms in the majority of potato varieties, but in some cases, they do induce mild leaf symptoms such as mosaics, rugosity, vein deepening, and bronzing [3]. PVS and PVM are transmitted by aphids and by mechanical means, as well as through infected seed tubers [3,6].

PVS has a ~8.5 Kb positive-sense single-stranded RNA genome that contains six conserved open reading frames (ORFs) encoding a 223 KDa (K) RNA-dependent RNA polymerase (RdRP) mediating viral genomic RNA replication; triple gene-block (TGB) proteins TGBp1 (25K), TGBp1 (12K), and TGBp3 (7K) mediating cell-to-cell movement; a 34K coat protein (CP); and an 11K terminal cysteine-rich nucleic acid binding protein (NABP) implicated in suppression of RNA interference (RNAi)-based antiviral defenses [7,8].

Meristem culture, thermotherapy, electrotherapy, cryotherapy, and chemotherapy and their combinations are the main methods to eliminate viruses from potato plants. These methods are sufficiently effective, but none of them protects the cured plants from new infections [2]. Identification of virus resistance genes and breeding for resistance are time-consuming and do not guarantee success [9]. Presently, genome editing and transgenic RNA interference (RNAi) are the main and most promising technologies for engineering virus-resistant plants [10,11].

RNAi is a conserved mechanism that regulates gene expression and defends against invasive nucleic acids such as transposons, transgenes, and viruses in most eukaryotes [12,13,14,15]. In plants, RNAi is mediated by Dicer-like (DCL), Argonaute (AGO), and RNA-dependent RNA polymerase (RDR) gene families [16,17]. RNAi is induced by double-stranded (dsRNA) or hairpin RNA molecules, which are processed by DCLs into 21–24 nt small RNA (sRNA) duplexes. Based mostly on 5′-terminal nucleotide identity and size, one strand of the sRNA duplex is incorporated into one of the AGO family proteins, forming an RNA-induced silencing complex [18]. DCL4 and DCL2 generate, respectively, 21 and 22 small interfering RNAs (siRNAs) from dsRNA intermediates of viral replication and thereby mediate defenses against RNA viruses via siRNA-directed and AGO-mediated cleavage and degradation of viral RNA [15,19]. Additionally in the case of DNA viruses, DCL3 generates 24 nt siRNAs that can potentially direct transcriptional silencing of viral genes in the nucleus. RDRs reinforce RNAi by converting aberrant single-stranded RNA into dsRNA precursors of secondary siRNAs [12,15]. To establish successful infection, both RNA and DNA viruses suppress or evade RNAi, as well as innate immunity-based antiviral defenses that work in concert with RNAi [20,21].

Boosting natural RNAi-based resistance to viral infection can be achieved by transgene expression of viral genome fragments in sense or antisense orientation, or as inverted repeats separated by a spacer or an intron. In the latter case, a so-called intron-hairpin RNA transcribed by RNA polymerase II (Pol II), after splicing and formation of dsRNA structure, is directly processed by DCL4, DCL2, and DCL3 into 21, 22, and 24 nt viral siRNAs, resulting in complete (or near-complete) immunity to cognate/target virus infection (for an example, see Reference [22]; reviewed in Reference [23]). Notably, the production of transgene-derived 24 nt siRNAs correlates with immunity to both DNA and RNA viruses [23].

In this study, we tested an intron-hairpin RNA-based stable transformation method to cure field cultivars of *Solanum tuberosum* from natural infections with PVS and/or PVM, or their mixed infections with PVY, and thereby obtained transgenic plants with durable resistance to carlavirus infection.

## 2. Materials and Methods

### 2.1. Construction of Intron-Hairpin RNA Expression Cassettes

Total RNA was extracted from PVS (Kazakh isolate MK442089)-infected potato leaves, using TRI Reagent (Molecular Research Center, Cincinnati, OH, USA), according to the manufacturer’s protocol. Reverse transcription (RT) was performed by using 5 μg total RNA, oligo(dT)18 primer, and Maxima Reverse Transcriptase (Thermo Fisher Scientific, Rockford, IL, USA) according to the manufacturer’s protocol. Then 884 and 732 nt coding sequences of the PVS CP and 25K (TGBp1) proteins, respectively, were amplified by PCR (94 °C for 5 min, followed by 30 cycles of 95 °C for 30 s, 57 °C for 30 s, and 72 °C for 60 seq, and the last extension for 5 min at 72 °C), using the PVS sequence-specific primers (Dataset S1A) containing restriction sites for subsequent cloning of the amplified cDNA fragments in forward and reverse orientations and vice versa into the binary vector pCAMBIA2300 via a derivative of pBluescriptSK+ carrying a *Ricinus communis* catalase gene *cat1* intron I. In the resulting constructs, the inverted repeats of CP or 25K sequences, separated by the intron, were inserted between the cauliflower mosaic virus (CaMV) 35S promoter and the nopaline synthase (NOS) terminator (Supplementary Figure S1A). The binary plasmids were mobilized to *Agrobacterium tumefaciens* pGV2260S by electroporation, using Gene Pulser II (Bio-Rad, Hercules, CA, USA) with the parameters 25 mF, 1.8 kV, and 200 Ohm. Transformed cells were selected on standard Luria-Bertani medium with kanamycin (50 mg/L) and then were used for stable transformation of potato plants.

### 2.2. Virus Detection by ELISA

Double-antibody sandwich enzyme-linked immunosorbent assay (ELISA) analysis of leaf samples of potato plants cultivated under field or greenhouse conditions was carried out for detection of PVS, PVM, and PVY, using BIOREBA kits (Switzerland) according to the manufacturer’s protocol. Absorbance values (A_405nm_ and A_492nm_) were recorded using EL311 Microplate Autoreader (BioTek Instruments, Winooski, VT, USA) after sample incubation with substrate for 1 h at room temperature (22 °C), with 3 repeats for each sample.

### 2.3. Potato Transformation

Stem internode explants of virus-infected potato plants were transformed by co-cultivation with *A. tumefaciens* strains carrying the intron-hairpin RNA expression constructs. The explants were co-cultivated with agrobacterial suspension (OD_600nm_ = 0.9) in the standard Murashige and Skoog (MS) salts containing liquid medium supplemented with 2% sucrose for 30 min and then placed on the solid callus medium (MS medium with 1.6% glucose, 0.05 mg/L naphthalene acetic acid, 0.1 mg/L trans-zeatin, and 0.7% plant agar) for 2 days for callus formation. The explants with calli were placed on the solid callus medium supplemented with 0.5 g/L cefotaxime for two weeks to eliminate agrobacteria. Then the explants were placed on the solid morphogenesis medium containing standard MS salts, 1.6% glucose, 40 mg/L adenine, 5 mg/L calcium pantothenate, 1 mg/L biotin, 1.5 mg/L trans-zeatin, 0.5 mg/L gibberellic acid, 0.7% plant agar, 0.5 g/L cefotaxime, and 25 mg/L kanamycin and cultivated for shoot regeneration. The regenerated shoots of 0.7–1.0 cm in size were cut and placed in vitro on the standard MS medium supplemented with 2.6% sucrose, 1 g/L glycine, 0.8% plant agar, and 0.25 g/L cefotaxime for rooting and further growth under sterile conditions.

### 2.4. Molecular Analysis of Putative Transgenic Plants

Total DNA was extracted from leaves of putative transgenic plants by a CTAB method [24] and was tested by PCR for the presence of respective transgenes, using PVS-specific and *cat1* intron-specific primers (Dataset S1A). PCR-positive plants were grown in the greenhouse for 6 months and were tested for the presence of PVS, PVM, and PVY every two months, using ELISA. Tubers of PCR positive and control lines obtained in the greenhouse were planted in a field plot with high viral background. Leaf samples were taken for virus detection by ELISA every month (three times per season).

### 2.5. RNA Extraction and Blot Hybridization Analysis of Transgenic Plants

Total RNA was extracted for potato-leaf tissues, using a CTAB-LiCl method as described previously [25], and was quantified by using a NanoDrop 2000c Spectrophotometer (Thermo Fisher Scientific, Rockford, IL, USA). Small RNA blot hybridization analysis was performed as described previously [26]. Briefly, 10 μg total RNA was loaded on 15% denaturing polyacrylamide–urea gel. After gel electrophoresis and gel staining with ethidium bromide, RNA was transferred onto nylon membrane Hybond-N+ by blotting, followed by UV crosslinking. The membrane was hybridized successively with two ^32^P-labeled DNA oligonucleotide probes specific for PVS 25K sequence and plant miRNA (miR160) (Dataset S1A).

### 2.6. Illumina Sequencing Analysis of Transgenic, Viral, and Plant sRNAs

RNA integrity was verified by capillary electrophoresis on LabChip GX (Perkin Elmer). Illumina sequencing was performed at Fasteris AG (www.fasteris.com), using TruSeq small RNA protocol for library preparation and multiplexing 28 libraries (including 5 potato libraries ALYU-75-79 and 23 non-potato libraries) in one SP flowcell of NovaSeq 6000, which yielded for the potato libraries 31,627,721 to 35,869,294 reads with Q30 = 94.42 to 94.66. The libraries were de-multiplexed, followed by adapter trimming. The resulting reads, most of which were within a 20–25 nt size range, containing functional plant miRNAs and siRNAs, as well as viral siRNAs (Dataset S2A), were first used for de novo assembly of complete viral genomes, using a pipeline previously developed for reconstruction of virus genomes from cultivated *Solanum* plants [27]. Briefly, redundant 20–25 nt reads were de novo assembled by using Velvet 1.2.10 and Oases 0.2.09, followed by filtering the Oases contigs though the *Solanum tuberosum* genome (PGSC_DM_v3_superscaffolds, PGSC_DM_v3_2.1.9_superscaffolds_unanchored, S_tuberosum_Group_Phureja_chloroplast_DM1-3-516-R44, and S_tuberosum_Group_Phureja_mitochondrion_DM1-3-516-R44 http://solanaceae.plantbiology.msu.edu/pgsc_download.shtml; accessed on 20 April 2020), using Burrows–Wheeler Aligner (BWA) 0.7.12 and scaffolding non-plant contigs with SeqMan module of Lasergene DNASTAR software suite 7.1.0. The SeqMan contigs were manually corrected (if needed) and verified by mapping back redundant 20–25 nt reads and analyzing the mapping results with MISIS-2 [28] to yield a consensus sequence of each viral genome.

For the analysis of plant, viral, and transgene-derived siRNAs, 20–25 nt (or 15–34 nt) reads from each library were mapped onto reference sequences of the *S. tuberosum* genome, the genomes of PVS (reconstructed only from the sample ALYU-75) and PVY (reconstructed from ALYU-75, ALYU-76 and ALYU-79 samples), and the intron-hairpin RNA construct expression cassette S-25K-AS (Supplementary Figure S1A; see Dataset S1B for the viral and transgene reference sequences). The mapped reads were analyzed by using in-house scripts to count plant-, viral-, and transgene-derived sRNAs sorted by size (20, 21, 22, 23, 24, 25 nt, total 20–25 nt), polarity (forward, reverse, and total), and 5’-terminal nucleotide identity (5’A, 5’C, 5’G, and 5’U) and to create count tables (Dataset S2).

## 3. Results and Discussion

### 3.1. Virus Elimination from Field Potato Cultivars Using Transgenic RNAi

Eleven potato cultivars naturally infected with up to three different viruses, including at least one carlavirus (PVS or PVM), as evaluated by ELISA (Table 1), were transformed with the intron-hairpin RNA constructs S-CP-AS, AS-CP-S, S-25K-AS, or AS-25K-S (Supplementary Figure S1A), as described in Materials and Methods. Transformants regenerated on the kanamycin (Km)-containing selective medium and PCR-positive for the respective transgenes were obtained for nine cultivars, including seven Kazakh cultivars (Albinka, Dunyasha, Fortuna, Kormilitsa, Orbita, Tokhar, and Zeren) and two foreign cultivars (Picasso and Sante) (Table 1). The transformation efficiency was independent of single (PVS or PVM), double (PVS+PVM or PVM+PVY), or triple (PVS+PVM+PVY) virus infection of parental plants (Table 1). Although the number of Km-resistant regenerants was approximately equal for CP (n = 192) and 25K (n = 198) constructs, the number of PCR-positive transformants was higher for the 25K constructs (60 vs. 36) (Supplementary Figure S1B), and some of the cultivars were transformed only with 25K constructs (Albinka, Kormilitsa, and Zeren) or only with CP constructs (Fortuna, Orbita, Sante, and Tokhtar) (Supplementary Table S1).

A total of fifty PCR-positive potato transformants (lines) grown up in vitro were placed in pots and cultivated under greenhouse conditions for six months (Supplementary Table S1). Every two months, they were tested for virus infection, using ELISA. No carlavirus infection was detected at most time points, including the last one in 18 lines, representing cultivars Albinka (7 of 8 tested lines), Dunyasha (4 of 11 lines), Kormilitsa (single tested line), Picasso (3 of 9 lines), Sante (one of 3 lines), and Zeren (2 of 5 lines) (Table 1). In the case of the cultivar Fortuna, which was initially infected with PVS and PVM, only one of the two carlaviruses was below detection (PVS) in one of the three lines (all with CP constructs), while original carlavirus infection was not eliminated from the cultivars Orbita (single line with CP construct was still infected with PVS) or Tokhtar (all nine lines with CP constructs were still infected with PVM). Although carlavirus elimination did not generally depend on the presence of any particular transgenic construct, a bigger number of carlavirus-free lines were obtained with 25K constructs (n = 15) than with CP constructs (n = 3) (Supplementary Table S1).

Remarkably, in the case of the cultivar Zeren, which was initially infected with PVS, PVM, and PVY, two of the five PCR-positive lines carrying 25K transgenes were free of any virus. Likewise, PVY infection was eliminated from three of the nine lines of Picasso and from all three lines of Sante, but it remained to be detectable in all eight lines of Albinka (Supplementary Table S1).

### 3.2. Field Trials of Transgenic Plants for Virus Resistance

Tubers obtained for PCR-positive transformant lines of the cultivars Albinka (n = 8), Dunyasha (n = 11), Fortuna (n = 3), Kormilitsa (n = 1), Picasso (n = 11), Sante (n = 3), and Zeren (n = 5), as well as progeny tubers of the respective non-transformed parental plants and three additional cultivars, were taken for 3-year field trials in 2017, 2018, and 2019 (Supplementary Table S2). Each line was tested for virus infection using ELISA three times per season. As a result of the first season, 17 of the 42 transformant lines were found to be virus-free, including 7 lines of Dunyasha (#44, #62, #67, #146, #162, #294, and #312); 3 lines of Picasso (#257, #382, and #384); 2 lines of each Albinka (#43, #46) and Zeren (#119, #336) each; and one line of each Fortuna (#360), Sante (#291), and Kormilitsa (#103) each. Seven of these lines (#43, #44, #62, #67, #46, #103, and #119) carried the S-25K-AS construct, four lines (#312, #336, #382, #384) carried the AS-25K-S construct, and six lines (#146, #162, #257, #291, #294, and #360) carried the AS-CP-S construct. The other 25 transformant lines were found to be infected with PVY alone (n = 7), PVM alone (n = 9), PVS alone (n = 2), PVM and PVY (n = 4), or PVS and PVY (n = 3) (Supplementary Table S2).

In 2018, tubers from 30 transgenic lines (including 13 virus-free lines) from the first season were again planted in the field (Supplementary Table S2). By the end of the second season, ELISA revealed no virus in any of the eleven tested lines of Dunyasha (#44, #61, #62, #65, #67, #146, #162, #293, #294, #312, and #374), one of the three lines of Fortuna (#360), one of the five lines of Zeren (#336), one of the five lines of Picasso (#257), and a single tested line of Kormilitsa (#103). Note that these five cultivars were infected by at least one carlavirus (with or without PVY) before transformation (Table 1). Single infections with PVY were detected in all five tested lines of Albinka, three of the four lines of Picasso (both initially coinfected with PVM and PVY), and in one of the five lines of Zeren (initially coinfected with PVS, PVM, and PVY). On the other hand, single infections with PVS were detected in two of the three lines of Fortuna (initially coinfected with PVS and PVM), while double infections with PVS and PVY were detected in a single tested line of Sante (initially coinfected with PVM and PVY) and three of the five lines of Zeren (Supplementary Table S2).

In 2019, field trials performed for 29 PCR-positive lines from the second season revealed that most of them were free of PVS (n = 23) and PVM (n = 27), but they were infected with PVY (n = 19) (Supplementary Table S2). No virus was detected in four of the eleven lines of Dunyasha (#61, #146, #162, and #294), two of the three lines of Fortuna (#360 and #361), and one line of Kormilitsa (#103). Single infections with PVY were detected in five of the eleven lines of Dunyasha (#44, #62, #65, #67, and #374), two of the five lines of Zeren (#119 and #336), three of the five lines of Albinka (#39, #46, and #48), all four tested lines of Picasso (#192, #194, #257, and #351), and a single tested line of Sante (#295). Single infections with PVS were detected in one of the eleven lines of Dunyasha (#293) and one of the three lines of Fortuna (#309). Double infections with PVS and PVY were detected in three of the five lines of Zeren (#38, #59, and #344), while triple infections with PVY, PVS, and PVM was detected in one of the five lines of Albinka (#74) (Supplementary Table S2). Thus, the 3-year field trials pointed at the development of resistance to both PVS and PVM but not PVY. Ten of the twenty-two lines exhibiting resistance to both PVS and PVM carried the S-25K-AS construct, while eight and four of those twenty-two lines carried AS-CP-S and AS-25K-S constructs, respectively. No carlavirus-resistant line was obtained with the S-CP-AS construct (Supplementary Table S2).

In conclusion, the tissue culture transgenic RNAi approach was found to be effective for elimination of both target (PVS) and non-target (PVM) carlaviruses from naturally infected potato cultivars with subsequent resistance to those two carlaviruses under the field conditions over 3 years of trials.

### 3.3. Small RNA Blot Hybridization and Illumina Sequencing Analyses of Transgenic Plants

Tubers of three transgenic lines carrying the S-25K-AS construct and representing the cultivars Dunyasha (#61) and Kormilitsa (#103), both being ELISA-negative for virus infection after the third year of trials, and the cultivar Zeren (line #119), which scored ELISA-positive for PVY alone after the third year of trials (Supplementary Table S2), were collected after the third year for analysis of transgene- and virus-derived siRNAs and reconstruction of virome components (if any). As controls, we collected tubers of a non-transformed plant of cultivar Zeren from the field (coinfected with PVS and PVY) and leaves of an in vitro grown transgenic plant of cultivar Zeren (line #119, cured from triple infection with PVS, PVM, and PVY). After tuber seeding and plant cultivation in a greenhouse for one month and leaf sampling, total RNA was isolated from the leaf samples of all plants for blot hybridization and Illumina sequencing analysis of sRNAs.

**Figure 1:**
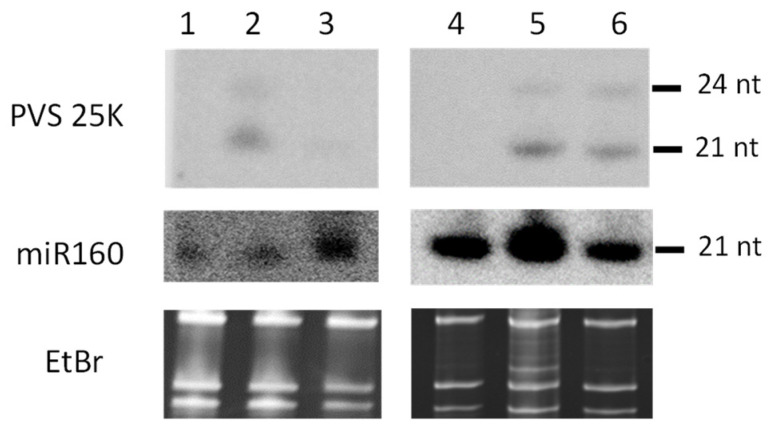
Blot hybridization analysis of sRNAs from transgenic potato lines. Total RNA extracted from leaf tissues of non-transformed potato plants of cultivars Zeren (Lane 1) and Ushkonyr (Lane 4) from the field trials; S-25K-AS transgene-carrying plants of the lines #119 (Lane 2), #61 (Lane 5), and #103 (Lane 6) from the field trials; and an in vitro propagated S-25K-AS transgenic plant of line #119 (Lane 3) was separated on 15% polyacrylamide–urea gel, blotted onto membrane, and UV-crosslinked. The membrane was successively hybridized with ^32^P-labled DNA oligonucleotide probes specific to the PVS 25K antisense strand and the plant miRNA miR160 (Dataset S1A). Positions of 21 and 24 nt transgene-derived siRNAs and 21 nt miR160 are indicated. The gel stained with ethidium bromide (EtBr) before blotting is shown as the loading control.

The small RNA blot hybridization analysis using a 22 nt DNA probe complementary to the PVS 25K antisense strand revealed that all the transgenic lines produce transgene-derived siRNAs of ca. 21–22 and 24 nts in size, with the in vitro line #119 accumulating lower levels of siRNAs than the field lines #119, #61, and #103, all carrying the same transgene (Figure 1, Lane 3 vs. Lanes 2, 5, and and 6). PVS-infection-derived viral siRNAs in the non-transformed Zeren plant were below detection (Figure 1, Lane 1).

To reconstruct virome components (if any) present in each leaf sample, we used a previously developed pipeline for de novo assembly of viral genomes from siRNAs [27,29] (see Section 2). As a result, complete genome sequences of PVS and PVY were reconstructed from the non-transformed field plant of Zeren (ALYU-75), while a single complete PVY genome was reconstructed from each field plant of the transgenic lines #119 (Zeren; ALYU-76) and #103 (Kormilitsa; ALYU-79). Interestingly, the PVS isolate ALYU-75 (deposited in NCBI GenBank under accession number ON583978) shares 98.4% identity with the PVS isolate KZ.Fortune (MK442089), which was identified in Kazakhstan in 2016 and was then used by us for construction of the intron-hairpin RNA transgenes. The PVY isolates ALYU-75 (ON583979) and ALYU-79 share 100% identity with each other (being 99.9% identical to JF927753 collected in 2007 in Poland) and 98.7% identity with the PVY isolate ALYU-76 (ON583980) (being 99.8% identical to JF927754 collected in 2007 in Germany; see Dataset S1B for the reconstructed viral sequences). No virus was reconstructed from the transgenic line #61 (ALYU-78) of Dunyasha from the field (cured from PVM infection) or from the in vitro propagated transgenic line #119 (ALYU-77) of Zeren (cured from triple infection with PVS, PVM, and PVY). These results confirmed our field ELISA analysis, with the exception for the transgenic Kormilitsa line #103, which scored negative for any virus (Dataset S2), suggesting that PVY escaped from ELISA detection at the sampling time point in the field. Notably, sRNA contigs representing a complete or near-complete PVS 25K coding sequence—obviously assembled from the PVS 25K transgene-derived siRNAs (see below)—were reconstructed from all the four transgenic plant samples.

To understand the biogenesis and function of intron-hairpin RNA transgene-derived siRNAs, we performed a bioinformatic analysis of their size, polarity, 5′-nucleotide identity. and hotspot profiles, in comparison with the respective profiles of virus (PVS and PVY)-derived siRNAs and plant sRNAs (Datasets S2 and S3).

**Figure 2:**
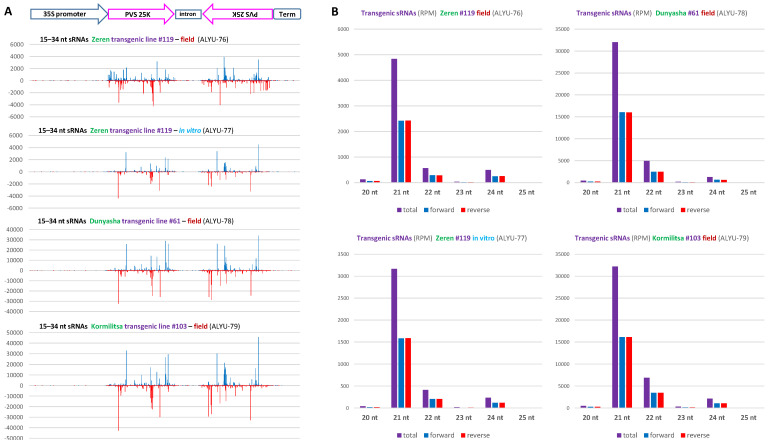
Single-nucleotide resolution maps and size profiles of transgene-derived sRNAs. (**A**) Single-nucleotide resolution maps of transgene-derived sRNAs. Redundant 15–34 nt sRNA reads obtained by Illumina sequencing of total RNA from leaf samples of the transgenic lines of cultivars Zeren (line #119: field plant ALYU-76 and in vitro plant ALYU-77), Dunyasha (line #61: field plant ALYU-78), and Kormilitsa (line #103: field plant ALYU-79) were mapped to the transgene expression cassette reference sequence (Dataset S1B) with zero mismatches; and single-nucleotide resolution sRNA maps were created by using MISIS-2 [28] (Dataset S3). The histograms plot the number of 15–34 nt sRNA reads at each nucleotide position of the 2743 bp transgene expression cassette. Bars above the axis represent sense (forward) reads starting at each respective position; those below represent antisense (reverse) reads ending at the respective position. The organization of the transgene expression cassette is shown schematically above the histograms: the CaMV 35S promoter, the PVS-25K-coding-sequence inverted repeats with the intervening intron, and the NOS terminator are delineated. (**B**) Size profiles and polarities of transgene-derived sRNAs. Redundant 20–25 nt sRNA reads mapped to the transgene expression cassette with zero mismatches were sorted by size (20, 21, 22, 23, 24, and 25 nt) and polarity (total, forward, and reverse) and counted in reads per million (RPM) of total 20–25 nt reads in each library (Dataset S2D). The resulting counts are plotted as bar graphs and color-coded.

Our analysis of single-nucleotide resolution maps and counts of 15–34 nt sRNAs revealed that, in each of the four transgenic plant samples, transgene-derived sRNAs are produced almost exclusively from the inverted repeats of the PVS 25K coding sequence and belong predominantly to the 21 nt class, followed by less abundant 22 and 24 nt classes (Figure 2 and Datasets S2 and S3). For each of the three major size classes, sense and antisense siRNAs are equally abundant (Figure 2B and Dataset S2A). The hotspot profiles of sense and antisense siRNAs differ substantially and mirror each other at the two inverted repeats (Figure 2A), apparently because the mapping tool BWA distributed the redundant reads of sRNAs derived from ascending and descending arms of the hairpin RNA randomly between the two repeats.

**Figure 3:**
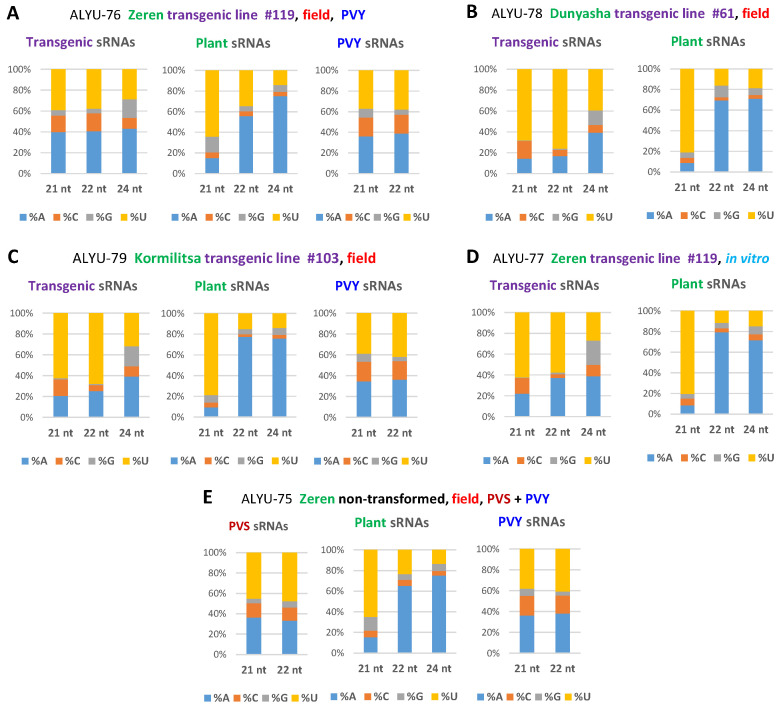
The 5′-nucleotide profiles of major size classes of transgenic, plant, and viral sRNAs. Redundant 20–25 nt sRNA reads from leaf samples of the transgenic lines of cultivars Zeren (line #119: field plant ALYU-76 and in vitro plant ALYU-77), Dunyasha (line #61: field plant ALYU-78), and Kormilitsa (line #103: field plant ALYU-79) and from a non-transformed Zeren plant (field plant ALYU-75) were mapped with zero mismatches to reference sequences of the transgene expression cassette, the *S. tuberosum* genome, and the reconstructed genomes of viruses (PVS and/or PVY) present in some of the plants (Dataset S1B); and the mapped sRNAs were sorted by size and 5′-terminal nucleotide identity (5′A, 5′C, 5′G, and 5′U) and counted (Dataset S2C). The frequencies of each 5′-nucleotide (in % of total) for each major size-class are plotted as bar graphs and color-coded. Panels show the resulting 5´-nucleotide profiles for the plants ALYU-76 (**A**), ALYU-78 (**B**), ALYU-79 (**C**), ALYU-77 (**D**) and ALYU-76 (**E**).

Our analysis of the 5′-terminal nucleotide identity of transgene-derived siRNAs revealed that 21 and 22 nt classes are enriched in 5′U (39 to 76%), followed by 5′A (14 to 41%) and 5′C (4 to 17%) (Figure 3A–D; Dataset S2C), suggesting their association with potato AGO1-, AGO2-, and AGO5-like proteins, respectively, as established in model plants (reviewed in Reference [18]). The 24 nt siRNAs were found to be enriched in 5′A (39 to 44%) and 5′U (27 to 39%) (Figure 3A–D; Dataset S2C), suggesting their association with potato AGO4-like and yet-to-be-identified AGO proteins, respectively.

Based on these findings that are generally consistent with those previously reported for intron-hairpin RNA transgenes [22,30,31,32,33], we assume that the PVS 25K inverted repeat-derived 21, 22, and 24 nt siRNAs are processed by, respectively, DCL4, DCL2, and DCL3 from the hairpin dsRNA structure of spliced and/or unspliced Pol II transcripts and then become associated with several AGO proteins to target complementary viral RNAs and thereby interfere with viral infection (reviewed in Reference [23]).

Negligible amounts of sRNAs derived from the transgene expression cassette outside of the PVS 25K inverted repeats (i.e., from the 35S promoter, intron, and NOS terminator regions) indicate limited involvement (if any) of RDR-dependent production of secondary siRNAs and transitive (transcriptional) silencing, thus explaining the robust production of transgene-derived siRNAs over three years of field trials and their durable protective effect. This is consistent with the results of field trials of intron-hairpin RNA transgenic tomato plants that exhibited immunity to tomato yellow leaf curl virus (genus *Begomovirus*) [22].

**Figure 4:**
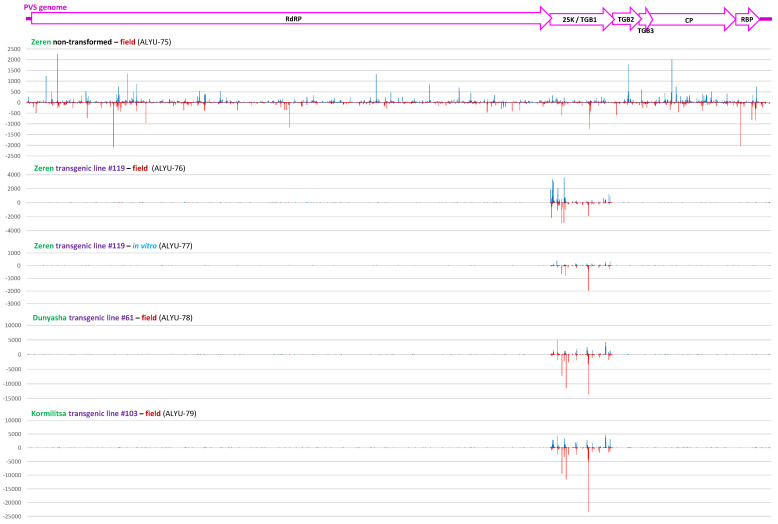
Single-nucleotide resolution maps of viral sRNAs from a PVS-infected non-transformed plant and transgene-derived sRNAs from PVS-free transgenic plants mapped on the PVS genome. Redundant 15–34 nt sRNA reads from the non-transformed Zeren plant (field plant ALYU-75) and the transgenic plant lines of cultivars Zeren (line #119: field plant ALYU-76 and in vitro plant ALYU-77), Dunyasha (line #61: field plant ALYU-78), and Kormilitsa (line #103: field plant ALYU-79) were mapped with zero mismatches to the PVS genome sequence (reconstructed from ALYU-75), and single-nucleotide resolution sRNA maps were created by using MISIS-2 [28] (Dataset S3). The histograms plot the number of 15–34 nt sRNA reads at each nucleotide position of the 8505 bp PVS genome. Bars above the axis represent sense (forward) reads starting at each respective position; those below represent antisense (reverse) reads ending at the respective position. The PVS genome organization is shown schematically above the histograms with conserved ORFs delineated and encoded proteins indicated.

**Figure 5:**
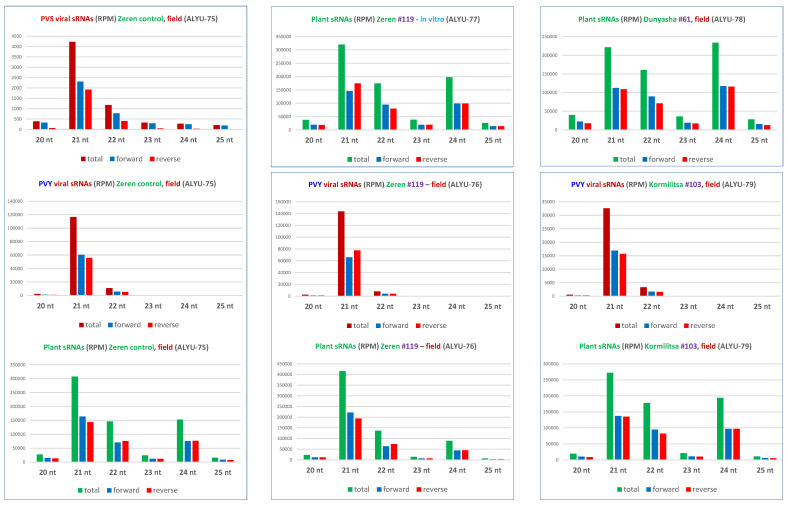
Size profiles of virus-derived and plant sRNAs. Redundant 20–25 nt sRNA reads from leaf samples of the non-transformed plant of cultivar Zeren (field plant ALYU-75) and the transgenic plant lines of cultivars Zeren (line #119: field plant ALYU-76 and in vitro plant ALYU-77), Dunyasha (line #61: field plant ALYU-78), and Kormilitsa (line #103: field plant ALYU-79) were mapped with zero mismatches to reference sequences of the reconstructed genomes of viruses (PVS and/or PVY) present in some of the plants (Dataset S1B) and the *S. tuberosum* genome. The mapped reads were sorted by size (20, 21, 22, 23, 24, and 25 nt) and polarity (total, forward, and reverse) and counted in reads per million (RPM) of total 20–25 nt reads in each library (Dataset S2D). The resulting counts are plotted as bar graphs and color-coded.

Our analysis of 15–34 nt sRNAs from the non-transformed Zeren plant (ALYU-75) coinfected with PVS and PVY revealed that viral siRNAs are derived from both strands of the entire PVS and PVY genomes and belong predominantly to the 21 nt and less abundant 22 nt classes, likely produced by DCL4 and DCL2, respectively, while other size classes accumulate at lower levels and are derived mostly from the sense strand (Figure 4 and Figure 5 and Datasets S2 and S3), suggesting their DCL-independent production by RNA decay pathways. Similar results were obtained for PVY-derived siRNAs accumulating in transgenic (ALYU-76 and ALYU-79) plants (Figure 5 and Datasets S2 and S3). In both PVS and PVY, the two major classes of viral siRNAs are enriched in 5′U (40 to 48%) and 5′A (33 to 38%), followed by 5′C (13 to 19%) (Figure 3 and Dataset S2C), suggesting their association with AGO1-, AGO2-, and AGO5-like proteins, respectively.

Mapping of sRNAs from the transgenic plants (ALYU-76, ALYU-77, ALYU-78, and ALYU-79) onto the PVS genome reconstructed only from the non-transgenic plant (ALYU-75) revealed that they match exclusively to the PVS 25K coding sequence (Figure 4 and Dataset S3), thus confirming their transgene origin and the absence of PVS infection in the transgenic plants (note that few reads outside of the PVS 25K sequence in ALYU-76, ALYU-77, ALYU-78, and ALYU-79 libraries represent cross-contaminating viral reads from the ALYU-75 library multiplexed and sequenced in the same flowcell). The 732 nt PVS 25K coding sequences present in the transgene and the PVS isolate ALYU-75 differ at 32 SNP (single nucleotide polymorphism) positions, and as ca. 15–25% of the transgene-derived 20–25 nt sRNAs mapped to the PVS ALYU-75 reference sequence with zero mismatches, almost 100% of those sRNAs mapped to the same reference sequence when up to two mismatches were allowed (Dataset S2A,B). This shows that transgene-derived siRNAs have sufficient homology for sequence-specific RNAi targeting of this particular isolate of PVS, as well as other genetic variants of this virus, provided that sufficient homology in their 25K coding sequence is preserved (see below).

**Figure 6:**
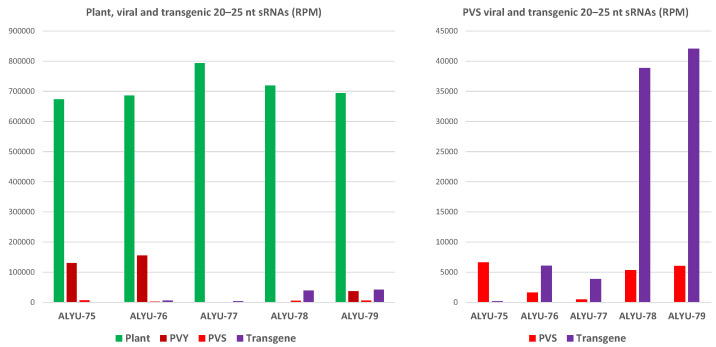
Relative abundance of plant, viral, and transgenic sRNAs. Redundant 20–25 nt sRNA reads from leaf samples of a non-transformed plant of cultivar Zeren (field plant ALYU-75) and the transgenic plant lines of cultivars Zeren (line #119: field plant ALYU-76 and in vitro plant ALYU-77), Dunyasha (line #61: field plant ALYU-78), and Kormilitsa (line #103: field plant ALYU-79) were mapped with zero mismatches to reference sequences of the *S. tuberosum* genome, the reconstructed genomes of viruses (PVS and/or PVY) present in some of the plants (Dataset S1B), and the S-25K-AS transgene expression cassette; and the sRNA reads mapped to each reference sequence were counted in reads per million (RPM) of total sRNAs in each library (Dataset S2D). The resulting counts are plotted as bar graphs and color-coded. Note that the transgenic plants ALYU-76, ALYU-77, ALYU-78, and ALYU-79 are not infected with PVS, and “PVS” reads (red bars) in these plants represent the PVS 25K transgene-derived siRNAs mapped to the 25K coding region of the PVS genome sequence reconstructed from the non-transgenic plant ALYU-75 infected with PVS.

Notably, counts of the PVS 25K transgene-derived siRNAs in the Zeren line #119 from the field (ALYU-76) and the whole PVS genome-derived viral siRNAs in the non-transformed Zeren plant from the field (ALYU-75) were comparable: 6617 and 6066 reads per million (RPM) of total 20–25 nt sRNAs, respectively (i.e., 0.66 and 0.61% of total sRNAs) (Figure 6 and Dataset S2D). Given that the 0.73 Kb PVS 25K repeat sequence in the transgene represents only 8.6% of the PVS genome length (8.5 Kb), the production rate of transgenic siRNAs is about 11 times higher than the production rate of viral siRNAs from the target region of PVS. This shows the strong potential of intron-hairpin RNA transgenes in boosting natural antiviral RNAi and accounts for both virus elimination and subsequent resistance of transgenic plants to new carlavirus infections.

Consistent with sRNA blot hybridization results, the in vitro propagated transgenic Zeren line #119 accumulated lower amounts of transgene-derived siRNAs (0.38% of total) than the field progeny of this line, whereas the field progenies of transgenic Dunyasha and Kormilitsa lines #61 and #103 accumulated higher amounts of transgene-derived siRNAs (3.9 and 4.2%, respectively). Since the Dunyasha line #61 is free of any virus, while the Kormilitsa line #103 is infected with PVY, PVY infection does not appear to interfere with production of transgene-derived siRNAs. This is despite the fact that the accumulation levels of PVY-derived viral siRNAs are much higher than those of PVS- and transgene-derived siRNAs (Figure 6 and Dataset S2D). A high abundance of potyviral siRNAs has been reported previously (in References [27,34], for example) and can potentially be explained by high replication and tissue tropism of potyviruses and/or an sRNA-stabilizing effect of the potyviral silencing suppressor HC-Pro that binds and sequesters viral siRNAs [34]. Our findings of durable resistance of some of the transgenic lines to PVS and/or PVM even in the presence of PVY indicate that siRNA binding activity of the PVY HC-Pro does not suppress the protective action of transgene-derived siRNAs against carlaviruses. Nonetheless, we cannot exclude the possibility that PVY infection or other biotic (or abiotic) factors can potentially interfere with the action of transgene-derived siRNAs, especially in those lines where their production rates are not high enough. This may explain why some of the transformant lines cured from carlavirus infection were eventually re-infected with PVS and/or PVM (Supplementary Table S2).

Our finding that intron-hairpin RNA transgenes had curing and protective effects not only against the target carlavirus PVS but also a non-target carlavirus (PVM) is consistent with the results of a previous study in which one of the cucumber lines expressing the highest amounts of intron-hairpin RNA transgene-derived 21, 22, and 24 nt siRNAs exhibited resistance to infections with both a target potyvirus and two non-target potyviruses that share 67% and 63% identity with the transgene sequence [30]. Our analysis of 22 isolates of PVM available in the NCBI GenBank revealed that most of these isolates have at least two >21 nt sequences with sufficient identity to the PVS 25K transgene sequence (Dataset S1C) for sequence-specific cleavage of PVM RNA by siRNAs derived from respective sequences of the transgene. Compared to 25K, CP sequences of the PVM isolates are too divergent from the PVS CP transgene sequence to predict their sequence-specific cleavage by transgenic siRNAs, although we cannot exclude inhibition of translation of PVM CP mRNA via complementary interaction of transgenic siRNAs with homologous viral sequences (Dataset S1C). Thus, mechanisms underlying the protective effects of intron-hairpin RNA transgenes against a non-target virus of the same genus observed in our study and by Leibman et al. [30] remain to be further investigated.

## Figures and Tables

**Table 1:** Initial infection status of potato cultivars and carlavirus resistance of PCR-positive transformants.

Cultivar	Virus Infection Status before Transformation	Number of Km-Resistant Regenerants	Number of PCR-Positive Regenerants	Number of Carlavirus-free Regenerants	Number of Carlavirus-Free Lines after 3 Years in Field
Albinka	PVM, PVY	19	8	7	3
Dunyasha	PVM	24	11	4	9
Fortuna	PVS, PVM	10	3	0	2
Kormilitsa	PVS	5	1	1	1
Orbita	PVM	6	1	0	not tested
Picasso	PVM, PVY	34	9	3	4
Sante	PVM, PVY	36	3	1	1
Tokhtar	PVM	80	9	0	not tested
Tian-Shanskiy	PVM	25	0	0	not tested
Ushkonyr	PVS	0	0	0	not tested
Zeren	PVS, PVM, PVY	20	5	2	2

## Data Availability

Viral genome sequences obtained in this study were deposited in the NCBI GenBank, with the accession numbers ON583978 (PVS isolate ALYU-75), ON583979 (PVY isolate ALYU-75), and ON583980 (PVY isolate ALYU-76).

## Supplementary Materials

The following supporting information can be downloaded at https://www.mdpi.com/article/10.3390/ijms23148020/s1.
