# Supplementary material for: Virus Elimination from Naturally Infected Field Cultivars of Potato (Solanum tuberosum) by Transgenic RNA Interference
Source: Int J Mol Sci. 2022 Jul 20;23(14):8020. doi: 10.3390/ijms23148020 (PMC9321115; doi:10.3390/ijms23148020)
Supplement: Supplementary file 1 [file ijms-23-08020-s001.zip › Table S2 IJMS.pdf]

Table S2. Field trials of PCR-positive transformant lines of naturally-infected potato cultivars

Cultivar (virus infection before transformation)	Line #	Construct used for transformation	Virus detection by ELISA		
			2017	2018	2019
**Albinka**<br>(**PVM** + PVY)	Non transformed parent	-	**PVM**	PVY	**PVM**<br>PVY
	**39**	S-25K-AS	PVY	PVY	PVY
	43	S-25K-AS	-	*	*
	**46**	S-25K-AS	-	PVY	PVY
	47	S-25K-AS	PVY	*	*
	**48**	S-25K-AS	**PVM**<br>PVY	PVY	PVY
	74	S-25K-AS	PVY	PVY	**PVS**<br>**PVM**<br>PVY
	159	S-25K-AS	PVY	PVY	*
	218	S-25K-AS	**PVM**<br>PVY	*	*
**Dunyasha**<br>(**PVM**)	Non transformed parent	-	-	**PVM**	**PVM**<br>PVY
	**44**	S-25K-AS	-	-	PVY
	**61**	S-25K-AS	**PVM**	-	-
	**62**	S-25K-AS	-	-	PVY
	**65**	S-25K-AS	**PVM**	-	PVY
	**67**	S-25K-AS	-	-	PVY
	**146**	AS-CP-S	-	-	-
	**162**	AS-CP-S	-	-	-
	293	AS-CP-S	**PVM**	-	**PVS**
	**294**	AS-CP-S	-	-	-
	312	AS-25K-S	-	-	**PVM**
	**374**	AS-25K-S	**PVM**	-	PVY
**Fortuna**<br>(**PVS** + **PVM**)	Non transformed parent	-	*	**PVS**	**PVS**
	309	AS-CP-S	**PVS**	**PVS**	**PVS**
	**360**	AS-CP-S	-	-	-
	**361**	AS-CP-S	**PVS**<br>PVY	**PVS**	-
**Kormilitsa**<br>(**PVS**)	Non transformed parent	-	*	*	*
	**103**	S-25K-AS	-	-	-
**Picasso**<br>(**PVM** + PVY)	Non transformed parent	-	*	*	*
	**192**	AS-CP-S	PVY	PVY	PVY
	**194**	AS-25K-S	**PVM**	PVY	PVY
	**257**	AS-CP-S	-	-	PVY
	**351**	AS-25K-S	**PVM**<br>PVY	PVY	PVY
	161	AS-CP-S	**PVM**	*	*
	172	AS-CP-S	PVY	*	*
	352	AS-25K-S	**PVM**<br>PVY	*	*
	381	AS-25K-S	PVY	*	*
	382	AS-25K-S	-	*	*
	383	AS-25K-S	**PVM**	*	*
	384	AS-25K-S	-	*	*
**Sante**<br>(**PVM** + PVY)	Non transformed parent	-	**PVM**	**PVM**<br>PVY	PVY
	**295**	AS-CP-S	**PVM**	**PVS**<br>PVY	PVY
	291	AS-CP-S	-	*	*
	339	AS-CP-S	**PVM**	*	*
**Zeren**<br>(**PVS** + **PVM** + PVY)	Non transformed parent	-	*	*	**PVS**<br>PVY
	38	S-25K-AS	**PVS**<br>PVY	**PVS**<br>PVY	**PVS**<br>PVY
	59	S-25K-AS	**PVS**<br>PVY	**PVS**<br>PVY	**PVS**<br>PVY
	**119**	S-25K-AS	-	PVY	PVY
	**336**	AS-25K-S	-	-	PVY
	344	AS-25K-S	**PVS**	**PVS**<br>PVY	**PVS**<br>PVY
Triumph	Non transformed	-	-	**PVM**<br>PVY	**PVM**<br>PVY
Imperatritsa	Non transformed	-	**PVM**	*	*
Milena	Non transformed	-	**PVM**	**PVM**<br>PVY	**PVM**<br>PVY

* not detected, because the plant died, or no tuber obtained in the previous season
- virus free
