# Supplementary material for: Virus Elimination from Naturally Infected Field Cultivars of Potato (Solanum tuberosum) by Transgenic RNA Interference
Source: Int J Mol Sci. 2022 Jul 20;23(14):8020. doi: 10.3390/ijms23148020 (PMC9321115; doi:10.3390/ijms23148020)
Supplement: Supplementary file 1 [file ijms-23-08020-s001.zip › Dataset S1 IJMS.pdf]

Dataset S1. Supplementary sequence information.

(**A**) Primers for cloning of PVS ORFs and probes for sRNA blot hybridization analysis
(**B**) Consensus sequences of *de novo* reconstructed viral genomes from the field potato samples ALYU-75 (cv. Zeren, non-transformed), ALYU-76 (cv. Zeren, transgenic line #119) and ALYU-79 (cv. Kormilitsa, transgenic line #103) and the reference sequence of the S-25K-AS RNAi transgene expression cassette (CaMV 35S promoter-PVS 25K ORF sense-*Ricinus communis* catalase gene *cat1* intron I-PVS 25K ORF antisense-NOS terminator)
(**C**) Multiple alignments of PVS and PVM 25K and CP coding sequences

Dataset S1A. Primers for cloning of PVS ORFs and probes for sRNA blot hybridization analysis.

Primers:

#302	5′-cgtatctagaatgccgcccaaaccggatcc-3′	XbaI
#304	5′-cgtaagatcttcattggttgatcgcattacggt-3′	BglII
#303	5′-cgtacccgggatgccgcccaaaccggatcc-3′	SmaI
#305	5′-cgtagtcgactcattggttgatcgcattacggt-3′	SalI
#306	5′-cgtaggatccttaggcggcgtgtaagtgg-3′	NcoI
#308	5′-cgtaggatccatgaggatatttgatagctt-3′	BamHI
#307	5′-cgtagagctcatgaggatatttgatagctt-3′	SacI
#309	5′-cgtagtcgacttaggcggcggtgtaagtgg-3′	SalI
intAS	5′-agtcgctatgtaatatttgcgcccataacc-3′	

Probes:

PVS_25K_s	5′-atatatggatgtgtttttgcaa-3′
miR160_as	5′-tggcatacagggagccaggca-3′

Dataset S1B. Consensus sequences of *de novo* reconstructed viral genomes from the field potato samples ALYU-75 (cv. Zeren, non-transformed), ALYU-76 (cv. Zeren, transgenic line #119) and ALYU-79 (cv. Kormilitsa, transgenic line #103) and the reference sequence of the S-25K-AS RNAi transgene expression cassette (CaMV 35S promoter-PVS 25K ORF sense-*Ricinus communis* catalase gene *cat1* intron I-PVS 25K ORF antisense-NOS terminator)

>PVS_ALYU-75_ON583978
TAAACACTCCCGAAAATAATTTGACTTAAACAACGCGACAGTTCAAGCAAATTACTTAACATGGCACTTACTTACAGAAGTCCAATCGAGGAAGTGTTAACACTACTAGAGCCTAATGCT
CAGTCCCTTaTTTCTAACGTCGCCACCAGCAGCTTTCAAGAGAGTGAGAAGGATAACTTCGCCTGGTTTTGCTACCATGTGTCGGCTAGCGCCAAGGAACACCTTAGTAGAGCAGGAATT
TACCTAAGCCCCTACTCGGGGTATCCTCATTCTCACCCGGTGTGCAAGACATTGGAAAATTACCTACTGTACAAAGTCCTACCACCACTTGTAAATAACACCTTTTACTTTGTAGGAATA
AAAGAATTCAAGCTCAATTTTCTTAAGAAGAGAATCAAACAAATGAGCATGATTCAAGCTATAAATAGGTATGTGAGCAGTGCCGATAAATTGCGATATGGTAATGAGTTCGTGATCAAA
TTCGGCGCGGCGTCGCCCGAACTCAAGCGGCACCATGGTTATTCACTGGATCCAGCCTTGCGTGATCTCTTGCCGAACATAAAGAGGGATTCTAATCTCTTCTTCCACGATGAGATGCAT
TATTGGGAAAAGAACCAGTTGATCAACTTCTTGGAACAATGCAGACCCAATACATGCTTGTGCACAATTGTGTACCCAACAGAGATATTCGTTGGGGCCCGACGTTCTTTGAATCCGTGG
GCATATGAGTTTGAGATCAAGAGAGATAAACTGCTCTTTTACCCAGATGGGGTGCGTAGTGAAGGCTATGAGCAGCCTGTCAACTGTGGGTATCTCCTCCGCACTAGAAAGATATTGCTG
AGGGATGGCACTATGTACAGTGTTGATCTAGTGTGCAGCAAATTCGCCCATCATCTAATAGCAATCACCAAGGGCGATTTGATCACCCCGACTTACCGTAGCTTCGGCCCTTTTGAGGCG
ATCAAGAGTGCGGGCCTGCAGGGGATAAGCAAAGGTAGGCCGAAATTCTATCCTGTACCGTGCCACATGATTTCTCGTTTATACAGGTACTTACGGTCtTTAAAGAAACCTGATAAGCAG
TCTGCAATGGCAAAATTCTCGCAGATGTGCCCTGAGCCGAGTGGTGACATGATAAGATTTATTGAGGAACTGAGTGATCTGATAATCAACACTGGCACTTTGAGGGTTATGATCGACGCA
GAACTGTGCAAAAATTTCTTTGGTAATCTGGGTCTGGCCTTACCGGCCACTCTCGCGTCAAAGATAAAAAGTACTCGTGCTGTAAGTTTAGAAGCCTTTATAGCCTCGCTTGAGCCGCTC
GTCGTTGATTGCGAATTGCAGACCATCTCGTGGGCCGTACCGCTGGCGCAGCTGTTGTTCAGTGAATCACCTGATGATCCCCCTGAGGATATGATTGAAGCAATGGATAGGAAGTGGGAG
AGTAGTAGCACGATGTTGTGCGACAGGGTCCCGGCACCTTACCGTGGGGATATGTGGAGCGGAACATCCCGCTCAATGAGCTTTTGGAGCATAGATTTTCAGCGGATCAAGTTTCTGAGA
GGGCTGATGGAATTGTACGTGGAAAGCATGTGCACAGAAGGCCTTGCAACCACCGTGACTTTTGAGTCTTACGTGGCCCAGATCGCGTCGTGTTGCTCGCTTCTCGGGCTCGCATTGATC
AAGTGCTTAACTGTTGCCGAGTATGCTGAGGTAGCCCAAATAGTGAGCAACACACGTTTGATTGATGTGCTCTTCGTTGCTGGGGACCTCCGTTGGTTCCGCGCCACCCGACATTCCAGG
CGTAACGTTAAGTTCTTGGACGAGACGGCCGATTGGGCAAGGTACAAAAGTGAGTTCGAGTGCGCAACATATGCAAAGCCCAAAGGAACAGGTCATATAGGTTATCTCCAAAACGTTGTG
TACAGCTTTTGCGGGGTGGGGGCACGATGGTCGTTTGACCCCGGCTACTGTTGCGAAAGTGATTCTGAGGCCACCCACTCTGACTACAGCGTAGTTGATCACCCCAGAGCAGCACCAATA
AATTCAGAAAGAACTTTGATCCAAGGGACCAGGTTGGGGAGAGCGTTGGGCTGCGCATGTGGGCTGCAGTCTGCAACAAGGGTGTTAGCGTTCCCCACGGAACACGGATTCAATTTGGAG
AGGGGGGTTTCAGGCAAGCGTGTTGCATGGTACTGCAGGGGTCAGGTCGATTATACATCCGGAGCTGTTTGCCATAAGAACTTGGGCTGGCCTAGATGGTTAAGCCAGTGGATGGAATTG
CATGAGATAGATGAGACGTACTATAATAGCATGCTGGCCCAGGAATTCCCTGCTGGCGGAGCTCTAGAGTGTagAGTGGGTGATGGAGGCCAGTTCATCCCgGGCTCAAATGTGGCCATA
GCTGAAGTTGGAGGTCAGTCCCAGGTTTCAATTGGCTGTGTGGCAGGAACTGGGCAGTTGTTGCTGGAATTGGGGGATTTCATTGAAGTGCCTGGTCCATGTTGGAGCAAGCACCATCTC
CACgTGCGCTGcAGCaAAATGaGCGGGGTGACGTTTATCTTCAGGCAAATTAAGGTTCCAGACCCAGTGGAGAATGCCGCAGTGGGGCAAGTCGCAACGCCTGCTTCAACAGCAGGTGCG
GGGAGGTCCAAGCTCAATGAGCATGATGCCCACCACACACGAGAGGGGGTCGCAGTGCATGCGTCCGGCAGGTGCCCAGCAGCAAAGAAATTCCATAGGGTACCTAATGCAGGTGGTGGA
GATTGTTTTTGGCTGGCCATCTCGCACTTCACAGGGGTGAGCGTGCAAGATATGAAACAGGGATTGCAACAGCTGGATTGGGAGAGTGATGCATTCAGCACCGAGTTAGCCTTGCAATTG
AAACCACAAGCTTGGGCTGAAGAGGAGGCCATTATCGCGACAAGTAAGCAATACCGGTACAGGATCGTGGTGCTGAGTGCCGATAAGGaGCAAACAGTCATTTATAGCCCGAAGGGCGAG
GCGGTGCAGTCCATGGTTCTATACCACTCCGGGGCCCACTTTGAGGCAGCTTTGCCCCGGAACGATTGCGTGCTTGTGGCTGTTGCGTCTGTCTTGCGGAGACGAGTTGAAGAGGTGCTT
TCAATTCTAGGTGCGCAGTTGGGTAATGAGTTTCTTCAGGATGTAATAAAGGGTGAAGGAATTAATCGGGACCAGTTGGCCATGGTTTTCAAACTCTTTGACATCTGCGCGCATGTGCAC
GCGGAGGGTGAGGTTTTCGTGATAAACTCTGAAGGTAGATTGCACGGCACATTCAATCTGAGCAAGGATCATATTGAGCATTGTAAGAGTAAACCGATGGGAATAACCAAATTCACAAGT
GTGCATGATGCTAGCTGTGAGATTAAGCAGGAAACACTCTCCATGCTTAAAGCCATGTGCACGTTGCTACCGTACAACCCATGTGAGCTTAGAGCAAAAGTGCTCGCTGATAGTTTGAAT
GCAGGCAGTACTGGGGTCCTCTGCGACGAGTTGTTCAATAAGGTCGGGAATTTACTCGAGGCAAATGAGGGGCGGCTGCGGGAGAATGTTAGAGAGGTGGGTTGCTTACTTGGAACTTTC
GGAGCTGGGAAGAGCATGGTCTTTAGGAAAGTGTTAAGTAGCAATCATGGGAAGAGCATTATCTACGTgTCTCCAAGAAAGCATTTGGCAGATTCATTCAATGAGCTTGTGAAGTCTATC
AAGCAGCAAGAGGGGGCAGCAAGTGTGCAAGGATTCCGCACCTTCACGTTCGAGAGAGCGCTCCTGAAGTGCGCGCAATTTAGGCCAGATGCAACGATCATCATTGATGAAATTCAGCTG
TTCCCACCAGGTTACTTGGATCTATTCTCTATGTTGGTACCAGCGGGGGTGCACATGTTCTTGGTGGGCGATCCGTGCCAAAGCGACTATGACTCAGAAAAAGACCGGAGCTTGTTTCAA
GCCATGAAATCCGACATCAATCTCCTGTTGGATGATGCGGATTATGATTTTAATTGCAGGAGTCGTAGATTTAAGGACAGGCTTTTTGATGGCCGTTTGCCATGCTCAATGGGGCCCATG
GAAGGGGAGCCATCCAAGTTCACAATCATTGAGGGCATTGAAAATTGTAAAGCCATTCACTCACAGGCCGAGGTTTGTTTAGTATCCTCGTTTGATGAAAAGAAGATAGTGCAGACTTAC
TTCCCGAGCTCTTGCCATTGCTTTACTTTTGGAGAATCAACGGGGATGACATACAAGTCTGGAGTGATACTGATCACAGACACCTCACAATACACCAGTGAGAGGAGGTGGTTAACTGCC
CTGAGCCGCTTCTCACATTCAATCGCCTTCGTGAATGCAACCGGTGGAAACATCCAGTTGGTGACCAGGTTATACCAAAATAGGGTTCTAGGTCGATTTCTGCTCAAAACTGCAAAGATC
GATGACCTTAAGATGTTGTTACCTGGTAGGCCACGCTTTAAGGAAGGATTTGGGGGTGAAAGAATTGGCGCAGATGAGGGCAAGAGAGAGTTCAAGTTGGAGGGTGATCCGTGGTTGAAA
ACTATGCTGGATCTACTACAGAAAGAGGATCAGGAGGAGGTTGAGGAAGCCGTCGTAGAGCTTGGTGAGGAATGGTTTCGCACACATTTGCCGCAATGCGAGCTGGAGGGCGTCAGAGCA
AGGTGGGTTGAAAGGATATTGGCAAAAGAAGTCCGTGAGAAGCGGATGGGGCTATTGGTCTCTGAGCAATTCACAGATGAGCATTCAAGGCAGTTGGGGAAGCAAATCACAAACGCCGCC
GAGAGGTTTGAAACTATCTACCCACGACATAGAGCTGCGGACACAGTCACTTTCATCATGGCTGTGAGGAAAAGATTGAGGTTTTCGGACCCAATTAGAGAGAGTGCAAAGCTCCGGGTT
GCGGAGATGTATGGGCCCTTCCTACTGAAAGAATTTCTCAAGCATGTGCCACTGAAACCAATGCATGACACAAGAATGATGGCTGAGGCAAAGTTTGATTTCGAGGAGAAGAAGACGCAG
AAGAGCGCAGCCACAATTGAGAACCACAGCAACAGATCTTGTAGAGACTGGCTGGCCGACATGGGCATGGTCTTCTCAAAGTCTCAACTCTGCACAAAGTTTGACAATCGGTTCAGGGAC
GCAAAAGCAGCGCAAACCATTGTCTGTTTCCAACATAGCGTCCTATGCCGCTTTGCCCCGTACATGAGGTACATTGAAAAGAAACTCAATGAAGTGTTACCGGCAAGGTTTTACATTCAT
TCAGGCAAAGGCTTGGAAGAGCTAAATAAATGGGTCATAGAGTCCAAATTCGATGGGTTGTGCACAGAGTCTGACTATGAAGCCTTCGACGCTAGTCAAGACCAGTACATAGTGGCGTTT
GAGCTAGCATTGATGAGGTATTTGGGCTTGCCCAATGATCTCATAGAGGATTACAAGTACATCAAAACGCACCTGGGCTCAAAGTTGGGGAACTTTGCCATAATGCGCTTCTCCGGTGAG
GCTAGCACCTTCTTGTTCAACACAATGGCCAACATGCTTTTCACATTCTTGAGATACAAGCTGAAGGGGGATGAGCGGATATGCTTCGCTGGTGACGACATGTGCGCCAACAGGGCCCTA
TTCATTAAAGATACTCATGAGGGCTTTCTTAAGAAGCTTAAGTTGAAGGCAAAGGTTGATAGGACAAACCGACCGAGTTTCTGCGGGTGGAGCTTGTGCTCGGATGGGATTTACaAAAAA
CCGCAGCTGGTCTTTGAGAGGCTTTGTATCGCCAAGGAAACGGCCAACTTGGCCAATTGCATTGACAATTATGCAATTGAGGTATCCTATGCCTACAAGCTCGGGGAGAGGATTAAGGAG
CGCATGTCGGAGGAGGAATTGGATGCCTTCTACAATTGTGTGAGAGTTATTATTAAGCATAAGCATCTGCTGAAGTCTGAGATCCGCTGTGTGTATGAGGATGTTTGATAGCTTAGGTAA
TCAGCTTAGTAGTATTGAATATATGGATGTGTTTTTGCAAATTTTGAATAAATATAAGTTTGAGCGTGTTAGTAGTACTTTAAATAAACCAATAGTTGTTCATAGTGTCCCAGGAGCTGG
TAAAAGTTCTGCAATTCGGGAGTTGCTTAAGTTAGATAGTAGGTTTGAGTGCATTACCCGTGGCCGGCCAGACATCCCGAATCTAGAGGGGGCCTTCATCAAGGCTGAGCGTGGTGGGGA
GAATAAATTGCTGCTGGTCGATGAGTACATAGAAGGGCCGGTCCCAGAGGACGCCTTTGCAATCTTTGCTGATCCACTTCAGAGTACCGCTGTGAGCCCATACCGGGCACACTTCATCAA
AACATTGAGCCATCGCTTTGGCAAGTGTACCGCTTCACTTTTGAGAGATTTGGGTTGGGACGTACAGGCAGAAGGTCAAGATTCAGTTCAAATTGCGGATATCTTCACAGTTGACCCTAG
GGATACTATCGTGTACTTCGAGCCGGAAGTTGGAGAGTTGCTGAGGAGCCACGGCGTCGAGGCGAGCTGCATTGGTGAGGTGCGCGGGGCTACTTTCGAACACGTAACCTTCGTCACTTC

TGAGAACAGCCCACTGGTTGATAAGGCTGCTGCTTTTCAGTGCTTGACGAGGCACACTAAGAGCTTGCTCATATTGTGCCCTGATGCCACTTACACCGCCGCCTAACTACACAGGGTTAT
ACATTGCAGCGGCATTAGGAGTGTCCCTTGCTGCCGTAGTAGCATTGTTCACTAGGAGTACATTGCCAATTGTTGGGGATTCGCAGCACAACCTCCCACACGGGGGACGGTATCGCGACG
GCACTAAGGCTATTGATTACTTCAAGCCCGCGAAACTGAATTCTGTTGAGCCTGGTAATCACTGGTACGCTCAACCTTGGCTACTAGTTCTACTTCTAGTTGCGCTCATCTGCTTATCAG
GGCGTCATGCTCCATGCTGTCCAAGGTGCAACCGAGTGCACAATGCTTAATGGTTTTCATCTTGGCATTCGCGCTAAGTTGGTATGCGCTCAGGCCAGGAAATACAAGCTGCGTTCTACT
CATCACTGGGGAATCAGTCCGGCTAGTCAATTGCGAGCTCACAAGAGATTTGGTGGAAGCCGTAGCAACATTGGGGCCGTTGAAGCACCTTTAGGTTCACAGGTAAGAGTTCGAAGAAAC
TGTCCCACAGAGAAAATGCCGCCCAAACCGGATCCAACAAGCTCAGGAGAGACACCACAAGCAACGCCGCTAGCGCCGCCGCCCCGGAACGTAGAGGAGCATAGAGTCGGCCCAAGTCAA
GGGCACGGGCAGAATGAAGAAGCTATGCTGGAGCAGAGGCTCATCAGATTGATTGAACTCATGGCCTCGAAAAGGCACAATTCAACATTGAGCAACATCTCTTTTGAGATAGGTAGGCCC
TCgCTTGAGCCGACCCCTGAAATGCGGAGGAATCCGGAGAACCCATACTCGCGGTTTTCAATCGATGAGCTGTTCAAGATGGAAATCCGATCTGTGTCCAACAACATGGCGAACACTGAG
CAAATGGCACAAATCACTGCTGACATCGCTGGGCTTGGGGTTCCAACTGAACACGTTGCAGGGGTAATACTGAAAGTGGTGATCATGTGTGCAAGCGTGAGTAGCTCTGTTTATCTAGAC
CCAGCAGGGACTGTAGAGTTCCCAACAGGCGCAGTGCCTTTGGACTCAATCATTGCAATTATGAAGAATCGCGCGGGATTGAGAAAAGTGTGCAGGCTGTATGCTCCAGTCGTGTGGAAT
TACATGCTAGTCCAGAATAGGCCACCTTCGGATTGGCAGGCCATGGGATTCCAGTGGAACGCACGCTTCGCCGCATTTGACACATTCGATTATGTGACTAATGGGGCTGCAATCCAGCCC
GTAGAGGGGCTCATACGCAGGCCCACACCTGAGGAGACAATAGCTCACAATGCCCACAAGAGTATGGCGATTGACAAGTCGAACAGAAATGAGCGATTGGCCAACACGAATGTTGAGTAC
ACTGGAGGGATGCTTGGCGCCGAGATTGTGCGCAACCACCGTAATGCGATCAACCAATGAAGGCAGACCGTTTAGCCATGTTATTATTGTGTGTCCATCGACTGGGATATGTTTTGCCAG
TTGAAGTTTGTGTAAATATAATAAGCCTAAGCGCAGGTCCAGTTTCTAGGGGCAGTTCCACTTACGCTCGTAAGCGGAGGGCCCGCAGCATTGGGCGTTGCTGGCGATGTTATCGTGTTT
ATCCACCTATTTGTAATTCTAAGTGTGATAATAGAACGTGCCGTCCAGGCATTAGTCAAAATTATAAAGTAGTGACTTTCATTCGGGGTTGGAGTAACTGAGGTGATACCACCCATGGTG
CAAAGTCAGAGTTTCGCATAAAACTTAAATAATATATAAGTGTGCAACTATAAAGAAAATATGTTTTTAAAATATTTTAGCAAAAAAAAAAAAAAAAAAAAAAAA

>PVY_ALYU-75_ON583979
AAATTAAAACAACTCAATACAACATAAGAAAAACAACGCAAAAACACTCATAAACGCTTATTCTCACTCAAGCAACTTGCTAAGTTTCAGTTTAAATCATTTCCTTGCAATTCTCTTAAA
CGATATTGGAAACCATTTCAACTCAACAAGTAATTTCATCACTTCCAACCAATTTTAGATCCTCAATGGCAACTTACATGTCAACAATCTGTTTCGGTTCGTTTGAATGCAAGCTACCAT
ACTCACCCGCCTCTTGCGGGCATATTGTGAAGGAACGAGAAGTGCTAGCTTCCGTTGATCCTTTCGCAGATCTGGAAACACAACTTAGTGCACGATTGCTCAAGCAAGAATATGCTACTG
TTCGTGTGCTCAAGAACGGTACTCTTACGTACCGATACAAGACTGATGCCCAGATAACGCGCATCCAAAAGAAACTGGAAAGGAAGGATAGGGAAGAATATCACTTCCAGATGGCAGCTC
CTAGTATTGTGTCAAAGATCACCATTGCTGGTGGAGAGCCACCTTCAAAACTTGAATCACAAGTGCGGAGGGGTGTCATCCACACAACTCCAAGGATGCGCACAGCAAAAACATATCGCA
CGCCAAAGTTGACAGAGGGACAAATGAACCACCTTATCAAGCAGGTGAAGCAAATTATGTCAACCAAAGGAGGGTCTGTTCAACTGATTAGCAAGAAAAGTACCCATGTTCACTATAAAG
AAGTTTTGGGATCACATCGCGCAGTTGTTTGCACTGCACATATGAAAGGTTTACGAAAAAGAGTGGACTTTCGGTGTGATAAATGGACCGTTGTGCGTCTACAGCATCTCGCCAGGACGG
ACAAGTGGAATAACCAAGTTCGTGCTACTGATCTACGCAAGGGCGATAGTGGAGTTATATTGAGTAATACTAATCTCAAAGGAAACTTTGGGAGAAGCTCGGAGGGCCTATTCATAGTGC
GTGGGTCGCACGAAGGAAAAATCTATGATGCACGTTCCAAGGTTACTCAAGGGGTTATGGATTCAATGGTTCAGTTCTCAAGCGCTGAAAGCTTTTGGAAGGGATTGGACGGCAATTGGG
CACAAATGAGATATCCTACAGATCATACATGTGTGGCAGGCTTACCAGTTGAAGACTGCGGCAGAGTTGCAGCGATAATGACACACAGTATTTTACCGTGCTATAAGATAACCTGCCCTA
CCTGTGCCCAACAATATGCCAACTTGCCAGCCAGTGACTTACTTAAGATATTACACAAGCACGCAAGTGATGGTTTAAATCGATTGGGGGCAGACAAAGATCGCTTTGTGCATGTCAAAA
AGTTCTTGACAATCTTAGAGCACTTAACTGAACCGGTTGATCTGAGTCTAGAAATTTTCAATGAAGTATTCAAGTCTATAGGGGAGAAGCAACAATCACCTTTCAAAAACCTGAATATTC
TGAATAATTTCTTTTTGAAAGGAAAGGAAAATACAGCTCGTGAATGGCAGGTGGCTCAATTAAGCTTACTTGAATTGGCAAGATTCCAAAAGAACAGAACGGATAATATCAAGAAAGGAG
ACATCTCGTTCTTTAGGAATAAACTATCTGCCAAAGCAAATTGGAACTTGTATCTGTCATGTGATAACCAGCTGGATAAGAATGCAAACTTCCTGTGGGGACAGAGGGAATATCATGCTA
AGCGATTTTTCTCGAACTATTTCGAGGAAATTGATCCAGCGAAGGGCTATTCAGCATATGAAAATCGTTTGCATCCGAATGGGACAAGAAAACTTGCAATTGGAAACCTAATTGTACCAC
TTGATCTGGCTGAGTTTAGGCGGAAGATGAAAGGCGATTATAAAAGACAGCCAGGGGTGAGTAAGAAGTGCACGAGCTCGAAGGATGGAAACTACGTGTATCCCTGTTGTTGCACTACAC
TTGATGATGGCTCAGCTGTTGAATCAACATTTTACCCGCCAACTAAGAAGCACCTCGTAATAGGTAATAGTGGCGACCAAAAGTATGTTGACTTACCAAAAGGGAATTCTGAGATGTTAT
ATATTGCCAAGCAAGGCTTCTGTTACATTAACATTTTCCTCGCAATGTTGATTAACATTAGTGAGGAAGATGCAAAGGATTTCACTAAGAAGGTTCGTGACATGTGTGTGCCAAAGCTTG
GAACCTGGCCAACCATGATGGATCTAGCCACAACTTGTGCTCAAATGAAAATATTCTACCCTGATGTTCATGATGCAGAACTGCCTAGAATACTAGTCGATCACGAAACGCAGACATGCC
ATGTGGTTGACTCGTTTGGCTCACAAACAACTGGGTATCATATTTTGAAAGCATCTAGCGTGTCCCAACTTATTTTGTTTGCTAATGATGAGTTGGAGTCTGACATTAAACATTATAGAG
TTGGTGGTGTTCCTAATGCATGCCCTGAACTTGGGTCCACAATATCACCTTTCAGAGAAGGAGGAGTTATAATGTCTGAGTCGGCAGCGCTGAAACTGCTTTTGAAAGGAGTTTTTAGAC
CTAAGGTGATGAGACAGTTGCTGTTAGATGAGCCTTACCTGTTGATTCTATCAATATTATCCCCTGGCATACTGATGGCTATGTACAATAATGGGATTTTTGAACTTGCGGTAAGGTTGT
GGATTAATGAGAAACAATCCATAGCTATGATAGCATCGCTACTATCAGCTTTAGCCCTACGAGTGTCAGCGGCAGAAACACTCGTCGCACAGAGGATTATAATTGATACTGCAGCTACAG
ATCTCCTTGATGCTACGTGCGATGGGTTCAACCTACATCTAACGTACCCCACTGCGTTGATGGTGTTGCAAGTTGTTAAGAATAGAAATGAATGTGATGATACCCTATTCAAGGCGGGTT
TTCCAAGTTACAACACGAGCGTCGTACAGATCATGGAAAAAAATTATCTAAATCTCTTGAACGATGCTTGGAAAGATTTAACTTGGCGGGAAAAATTATCCGCAACATGGTACTCATACA
GAGCAAAACGCTCTATCACTCGGTACATAAAACCCACAGGAAGGGCAGATTTGAAAGGGTTATACAACATATCACCACAAGCATTCTTGGGCCGAGGCGCCCAGGTGGTCAAAGGCACTG
CCTCAGGATTGAGCGAGCGATTTAATAATTATTTCAATACTAAGTGTGTAAATATTTCATCCTTTTTCATTCGTAGAATCTTTAGGCGTTTGCCAACTTTCGTCACTTTTGTTAACTCAT
TATTAGTTATTAGTATGTTAACCAGCGTAGTGGCAGTGTGTCAGGCAATAATTTTAGATCAGAGGAAGTATAGGAGAGAAATCGAGTTGATGCAGATAGAGAAGAATGAGATCGTCTGCA
TGGAGCTATATGCAAGTTTACAGCGCAAACTTGAACGCGATTTCACATGGGATGAGTACATTGAGTATTTGAAGTCAGTAAACCCTCAGATAGTTCAGTTTGCTCAAGCGCAGATGGAAG
AATATGATGTGCGACACCAACGTTCCACACCAGGTGTTAAAAATTTGGAACAAGTGGTAGCATTTATGGCTTTAGTCATCATGGTGTTCGATGCTGAAAGGAGTGATTGCGTGTTTAAAA
CTCTCAATAAATTTAAGGGTGTCCTTTCCTCACTGGACCATGAAGTTAGACATCAGTCCTTAGACGATGTGATCAAGAATTTTGATGAGAGGAATGAGATTATTGATTTTGAATTGAGTG
AGGACACAATTCGAACATCATCAGTGCTAGATACAAAGTTTAGTGATTGGTGGGACCGACAAATCCAGATGGGACATACACTTCCACATTACAGAACCGAGGGGCACTTCATGGAATTCA
CAAGAGCAACTGCTGTCCAAGTGGCTAATGACATTGCCCATAGCGAACACCTAGACTTTCTAGTAAGGGGAGCTGTTGGGTCTGGAAAGTCAACTGGGTTGCCTGTTCATCTTAGTGTAG
CCGGATCTGTGCTTTTAATTGAACCAACGCGACCACTAGCGGAAAACGTTTTCAAACAGCTATCTAGTGAACCATTCTTCAAGAAGCCAACACTGCGTATGCGTGGAAATAGTATATTTG
GCTCTTCTCCAATCTCCATCATGACTAGCGGATTTGCGCTACACTACTTCGCCAATAATCGCTCTCAATTAGCTCAGTTCAACTTTGTAATATTTGATGAGTGCCATGTTCTGGATCCTT
CCGCAATGGCGTTCCGTAGTCTGCTGAGTGTTTATCATCAAGCATGCAAAGTATTAAAAGTGTCAGCTACTCCAGTGGGAAGAGAGGTTGAATTTACAACACAGCAACCAGTCAAGTTAA
TAGTGGAGGACACACTGTCTTTCCAATCATTTGTTGATGCACAAGGTTCTAAAACTAATGCTGATGTTGTTCAATTTGGTTCAAACGTACTTGTGTACGTGTCGAGCTACAATGAAGTTG
ACACCTTGGCTAAGCTCCTAACAGACAAGAATATGATGGTCACAAAGGTTGATGGCAGAACAATGAAGCACGGTTGCCTAGAAATTGTCACAAAGGAACCAGTGCGAGACCACATTTTG
TTGTAGCAACCAACATAATTGAGAATGGAGTAACTTTGGACATAGACGTGGTTGTAGATTTTGGGTTGAAAGTCTCACCGTTCTTGGACATTGACAATAGGAGCATTGCTTACAATAAGG
TGAGTGTTAGCTATGGTGAGAGAATTCAAAGGCTGGGTCGTGTTGGACGCTTCAAGAAAGGAGTAGCATTGCGCATTGGACACACTGAGAAGGGAATTATTGAAATTCCAAGCATGATCG
CTACAGAGGCGGCTCTTGCTTGCTTTGCATATAACTTGCCAGTGATGACAGGAGGCGTTTCAACTAGTCTGATTGGTAATTGTACTGTGCGCCAAGTTAAAACAATGCAGCAATTTGAAT
TGAGTCCCTTCTTTATCCAGAATTTCGTTGCCCATGATGGATCAATGCATCCTGTCATACATGACATTCTTAAAAAGTATAAACTTCGAGATTGTATGACACCTTTGTGCGATCAATCTA
TACCATACAGGGCATCGAGCACTTGGTTATCGGTTAGTGAATATGAGCGACTTGGAGTGGCCTTAGAAATTCCAAAGCAAGTCAAAATTGCATTCCATATCAAAGAGATCCCTCCTAAGC
TCCACGAAATGCTTTGGGAAACGGTTGTCAAGTATAAAGACGTTTGCTTATTTCCAAGCATTCGAGCATCGTCCATCAGCAAAATCGCATACACATTGCGTACAGACCTCTTCGCCATCC
CAAGAACTCTAATATTGGTGGAGAGACTGCTTGAAGAAGAGCGAGTGAAGCAGAGCCAATTCAGAAGTCTCATCGACGAAGGATGCTCAAGCATGTTTTCAATTGTCAACTTGACAAACA
CTCTCAGAGCTAGATATGCAAAAGATTACACCGCAGAGAACATACAAAAACTTGAGAAAGTGAGAAGTCAATTGAAAGAATTCTCAAATTTGGATGGTTCTGCATGTGAGGAAAATTTAA
TAAAGAGGTATGAGTCTTTGCAGTTCGTTCATCACCAAGCTGCGACGTCACTTGCAAAGGATCTCAAGTTGAAGGGGACTTGGAAGAAGTCATTAGTGGCCAAAGACTTGATCATAGCAG
GCGCTGTTGCAATTGGTGGAATAGGACTTATATATAGTTGGTTCACACAATCAGTTGAGACTGTGTCTCACCAAGGGAAAAATAAATCCAAAAGAATTCAAGCCTTGAAGTTTCGCCATG
CTCGTGACAAAAGGGCTGGCTTTGAAATTGACAACAATGATGACACAATAGAGGAATTCTTTGGATCTGCATACAGGAAAAAGGGAAAAGGTAAAGGTACCACAGTTGGTATGGGCAAGT
CAAGCAGGAGGTTCATCAACATGTATGGGTTTGATCCAACAGAGTACTCATTCATCCAATTCGTTGATCCACTCACTGGGGCGCAAATAGAAGAGAATGTCTATGCTGACATTAGAGATA
TTCAAGAGAGATTTAGTGAGGTGCGAAAGAAAATGGTTGAGAATGATGACATTGAAATGCAAGCCTTGGGCAGTAACACGACCATACATGCATACTTCAGGAAAGATTGGTCTGACAAAG
CTTTGAAGATTGATTTAATGCCACATAATCCACTCAAAGTTTGTGACAAGACAAaTGGCATTGCCAAATTTCCTGAGAGAGAGCTTGAACTAAGGCAGACTGGGCCAGCTGTAGAAGTCG
ACGTGAAGGACATACCAACACAGGAGGTGGAGCATGAAGCTAAATCGCTCATGAGAGGCTTGAGAGACTTCAACCCAATTGCCCAAACAGTTTGTAGGCTGAAAGTATCTGTTGAATATG
GAACATCAGAGATGTACGGTTTTGGATTTGGAGCATATATAATAGCGAACCACCATTTGTTCAGGAGTTACAATGGTTCCATGGAGGTGCGATCCATGCACGGTACATTCAGGGTGAAGA
ATCTACACAGTTTGAGTGTTCTGCCAATTAAAGGTAGGGATATTATCCTCATCAAAATGCCGAAAGATTTCCCTGTCTTCCCACAGAAATTGCATTTCCGAGCTCCTACACAGAATGAAA
GAGTTTGTTTAGTTGGAACCAACTTTCAGGAGAAGTATGCATCGTCGATCATCACAGAAACAAGCACCACTTACAATATACCAGGCAGCACATTCTGGAAGCATTGGATTGAAACAGATA
ATGGACATTGTGGACTACCAGTGGTGAGCACCACCGATGGATGTCTAGTCGGAATTCACAGTTTGGCAAACAACAAACACACCACGAACTACTACTCAGCCTTCGATGAAGATTTTGAAA
GCAAGTATCTCCGAACCAATGAGCACAATGAATGGGTCAAGTCTTGGATTTATAATCCAGACACAGTGTTGTGGGGCCCGTTGAAACTTAAAGACAGCACTCCCAAAGGACTATTCAAAA
CAACAAAGCTTGTGCAAGATCTAATCGATCATGATGTAGTGGTGGAGCAAGCTAAGCACTCTGCGTGGATGTTTGAAGCCTTGACAGGAAATTTGCAAGCTGTCGCAACAATGAAGAGCC
AATTAGTAACCAAGCATGTAGTTAAAGGAGAGTGTCGACACTTCAAAGAATTCCTGACTGTGGATGCAGAAGCAGAGGCATTCTTCAGGCCTTTGATGGATGCGTATGGGAAAAGCTTGC
TGAATAGAGATGCATACATCAAGGACATAATGAAGTATTCAAAACCTATAGATGTTGGTATCGTGGACTGTGATGCATTTGAGGAAGCCATCAATAGGGTTATCATCTACCTGCAAGTGC
ACGGCTTCAAGAAGTGCGCATACGTCACTGACGAGCAAGAAATTTTCAAAGCGCTCAACATGAAAGCTGCAGTCGGAGCCATGTATGGTGGCAAAAAGAAGGACTATTTTGAGCATTTCA
CTGATGCAGATAAGGAAGAAATAGTCATGCAAAGCTGTCTGCGATTGTATAAAGGCTTGCTTGGCATTTGGAATGGATCATTGAAGGCAGAGCTCCGGTGTAAGGAAAAGATACTTGCAA
ATAAGACGAGGACATTCACTGCTGCACCTTTAGACACTTTGCTGGGTGGTAAAGTGTGTGTTGATGACTTCAATAATCAATTTTATTCAAAGAATATTGAATGCTGTTGGACGGTTGGGA
TGACTAAGTTTTATGGTGGTTGGGATAAACTGCTGCGGCGTTTGCCTGAGAATTGGGTATACTGTGATGCCGATGGCTCACAGTTTGATAGTTCACTAACTCCATACTTAATCAATGCTG
TTCTCACCATTAGAAGCACATACATGGAAGATTGGGATGTGGGGTTGCAAATGTTGCGCAATTTATACACTGAGATTGTTTACACACCTATTTCAACTCCAGATGGAACAATTGTTAAGA
AGTTCAGAGGAAATAACAGTGGTCAGCCTTCTACTGTTGTGGACAACTCTCTTATGGTCGTCCTTGCCATGCACTATGCTCTCATCAAAGAATGCATTGAGTTTGAAGAGATTGACAGCA
CGTGCGTATTCTTTGTCAATGGTGATGATTTGCTGATTGCTGTGAATCCGGATAAAGAGGGCATTCTTGACAGATTGTCACAACACTTCTCAGATCTTGGTTTGAATTATGATTTCTCGT
CAAGAACAAGAAATAAGGAGGAATTGTGGTTTATGTCTCATAGAGGCCTACTGATTGAGGGCATGTACGTGCCGAAACTTGAAGAAGAAAGGATTGTGTCCATTCTCCAATGGGACAGAG
CAGACTTGGCTGAACATAGGCTTGAAGCGATTTGCGCAGCTATGATAGAATCCTGGGGTTATTCTGAACTAACACACCAAATCAGGAGATTCTACTCATGGCTATTGCAACAGCAACCCT
TTGCAACAATAGCGCAGGAAGGGAAGGCTCCTTATATAGCAAGCATGGCATTAAGGAAATTGTATATGGATAGGGCTGTGGATGAGGAAGAGCTGAGAGCTTTCACTGAAATGATGGTCG
CATTAGACGATGAGTTTGAATTTGACTCTTATGAAGTATACCATCAAGCAAATGACACAATCGATGCAGGAGGAAGCAGCAAGAAAGATGCAAGACCAGAGCAAGGCAGCATCCAGTCAA
ACCCGAACAAAGGAAAAGATAAGGATGTGAATGCTGGTACATCTGGGACACATACTGTGCCGAGAATCAAGGCTATCACGTCCAAAATGAGAATGCCCAAAAGCAAGGGAGCAACCGTGC
TAAACTTAGAACACTTGCTTGAGTATGCTCCACAACAAATTGATATTTCAAATACTCGGGCAACTCAATCACAGTTTGATACGTGGTATGAGGCAGTGCGGATGGCATACGACATAGGAG
AAACTGAGATGCCAACTGTGATGAATGGGCTTATGGTTTGGTGCATTGAAAATGGAACCTCGCCAAATGTCAACGGAGTTTGGGTTATGATGGATGGGGATGAACAAGTCGAGTACCCGT
TGAAACCAATCGTTGAGAATGCAAAACCAACCCTTAGGCAAATCATGGCACATTTCTCAGATGTTGCAGAAGCGTATATAGAAATGCGCAACAAAAAGGAACCATATATGCCACGATATG
GTTTAATTCGAAATCTGCGGGATGTGGGTTTAGCGCGTTATGCCTTTGACTTTTATGAGGTCACATCACGAACACCAGTGAGGGCTAGGGAAGCGCACATTCAAATGAAGGCCGCAGCAT

TGAAATCAGCCCAACCTCGACTTTTCGGGTTGGACGGTGGCATCAGTACACAAGAGGAGAACACAGAGAGGCACACCACCGAGGATGTCTCTCCAAGTATGCATACTCTACTTGGAGTTA
AGAACATGTGATGTAGTGTCTCTCCGGACGATATATAAGTATTTACATATGCAGTAAGTATTTTGGCTTTTCCTGTACTACTTTTATCATAATTAATAATCAGTTTGAATATTACTAATA
GATAGAGGTGGCAGGGTGATTTCGTCATTGTGGTGACTCTATCTTTTAATTCCGCATTATTAAGTCTTAGATAAAAGTGCCGGGTTGTCGTTGTTGTGGATGATTCATCGATTAGGTGAT
GTTGCGATTCTGTCGTAGCAGTGACTATGTCTGGATCTATCTGCTTGGGTGGTGTTGTGATTTCGTCATAACAGTGACTGTAAACTTCAATCAGGAGACAAAAAAAAAAAAAAAAAAAA
AAAAAAAAAAAAAAAAAAAAAAAAAAAAAAAA

>PVY_ALYU-76_ON583980
AAATTAAAACAACTCAATACAACATAAGAAAAACAACGCAAAAACACTCATAAACGCTTATTCTCACTCAAGCAACTTGCTAAGTTTCAGTTTAAATCATTTCCTTGCAATTCTCTTAAA
CGATATTGGAAACCATTTCAACTCAACAAGTAATTTCATCACTTCCAACCAATTTTAGATCCTCAATGGCAACTTACATGTCAACAATCTGTTTCGGTTCGTTTGAATGCAAGCTACCAT
ACTCACCCGCCTCTTGCGGGCATATTGTGAAGGAACGAGAAGTGCTGGCTTCCGTTGATCCTTTCGCAGATCTGGAAACACAACTTAGTGCACGATTGCTCAAGCAAGAGTATGCTACTG
TTCGAGTGCTCAAGAACGGTACTCTTACGTACCGATACAAGACTGATGCCCAGATAACGCGCATCCAGAAGAAACTGGAAAGGAAGGATAGGGAAGAATATCACTTCCAGATGGCAGCTC
CTAGTATTGTGTCAAAGATCACTATTGCTGGTGGAGAGCCACCTTCAAAACTTGAATTACAAGTGCGGAGGGGTGTCATCCACACAACTCCAAGGATGCGCACAGCAAAAACATATCACA
CGCCAAAGTTGACAGAGGGACAAATGAACCACCTTATCAAGCAGGTGAAGCAAATTATGTCAACCAAAGGAGGGTCTGTTCAACTGATTAGCAAGAAAAGTACCCATGTTCACTATAAAG
AAGTTTTGGGATCACATCGCGCAGTCGTTTGCACTGCACATATGAGAGGTTTACGAAAGAGAGTGGACTTTCGGTGTGATAAATGGACCGTTGTGCGTCTACAGCATCTCGCTAGGACGG
ACAAGTGGACTAACCAAGTTCGTGCTACTGATCTACGCAAGGGCGATAGTGGAGTTATATTGAGTAATACTAATCTCAAAGGAAACTTTGGGAGAAGCTCGGAGGGCCTATTCATAGTGC
GTGGGTCGCACGAAGGAAAAATCTATGATGCACGTTCCAAGGTTACTCAAGGGGTTATGGATTCAATGGTTCAGTTCTCAAGCGCTGAAAGCTTTTGGAAGGGATTGGACGGCAATTGGG
CACAAATGAGATATCCTACAGATCATACATGTGTGGCAGGCTTACCAGTTGAAGACTGTGGCAGAGTTGCAGCGATAATGACACACAGTATTTTACCGTGCTATAAGATAACCTGCCCTA
CCTGTGCCCAACAATATGCCAACTTGCCAGCCAGTGACTTACTTAAGATATTACACAAGCACGCAAGTGATGGTTTAAATCGATTGGGGGCAGACAAAGATCGCTTTGTGCATGTCAAAA
AGTTCTTGACAATCTTAGAGCACTTAACTGAACCGGTTGATCTAAGTCTAGAAATTTTCAATGAAGTATTCAAGTCTATAGGGGAGAAGCAACAATCACCTTTCAAAAACCTGAATATTC
TGAATAATTTCTTTTTGAAAGGAAAGGAAAATACAGCTCGTGAATGGCAGGTGGCTCAATTAAGCTTACTTGAATTGGCGAGATTCCAAAAGAACAGAACGGATAATATCAAGAAAGGAG
ACATCTCGTTCTTTAGGAATAAACTATCTGCCAAAGCAAATTGGAACTTGTATCTGTCATGCGATAACCAGCTGGATAAGAATGCAAACTTCCTGTGGGGACAGAGGGAATATCATGCTA
AGCGATTTTTCTCGAACTACTTCGAGGAAATTGATCCAGCGAAGGGCTACTCAGCATACGAAAATCGTTTGCATCCGAATGGGACAAGAAAACTTGCAATTGGAAACCTAATTGTACCAC
TTGATCTGGCTGAGTTTAGGCGGAATATGAAAGGTGATTATAAAAGACAGCCAGGGGTGAGTAAGAAGTGCACGAGCTCGAAGGATGGAAACTACGTGTATCCCTGTTGTTGCACTACAC
TTGATGATGGCTCAGCTGTTGAATCAACATTTTACCCGCCAACTAAGAAGCACCTCGTAATAGGTAATAGTGGCGACCAAAAGTATGTTGACTTACCAAAAGGGAATTCTGAGATGTTAT
ATATTGCTAGGCAAGGCTTCTGTTACATTAACATTTTCCTCGCGATGTTGATTAACATTAGTGAGGAAGATGCAAAGGATTTCACTAAGAAGGTTCGTGACATGTGTGTGCCAAAGCTTG
GAACCTGGCCAACCATGATGGATCTGGCTACAACTTGTGCTCAAATGAAAATATTCTACCCTGATGTTCATGATGCAGAACTGCCTAGAATACTAGTCGATCACGAAACGCAAACATGCC
ATGTGGTTGACTCGTTTGGCTCACAAACAACTGGGTATCATATTTTGAAAGCATCTAGCGTGTCCCAACTTATTTTGTTTGCTAATGATGAGTTGGAGTCTGACATTAAGCATTATAGAG
TTGGTGGTGTTCCTAATGCATGCCCTGAACTTGGGTCCACAATATCACCTTTCAGAGAAGGAGGAGTTATAATGTCTGAGTCGGCAGCGCTGAAACTGCTTTTAAAGGGAATTTTTAGAC
CTAAGGTGATGAGACAGTTGCTGTTAGATGAGCCTTACCTGTTGATTCTATCAATATTATCTCCTGGCATACTGATGGCTATGTATAACAATGGGATTTTTGAACTTGCGGTAAGGTTGT
GGATTAATGAGAAACAATCCATAGCTATGATAGCATCGCTACTATCAGCTTTAGCCCTACGAGTGTCAGCGGCAGAAACACTCGTTGCACAGAGGATTATCATTGATGCTGCAGCTACAG
ACCTCCTTGATGCTACGTGTGATGGGTTCAACCTACATCTAACGTACCCTACTGCATTGATGGTGTTGCAAGTTGTTAAGAATAGAAATGAATGTGATGATACCCTATTCAGGGCGGGTT
TTTCAAGTTATAACACAAGCGTCGTACAGATTATGGAAAAAAATTATCTAAATCTCTTGGACGATGCTTGGAAAGATTTAACTTGGCGGGAAAAATTATCCGCAACATGGTACTCATACA
GAGCAAAACGCTCTATCACTCGGTACATAAAACCCACAGGAAGGGCAGATTTGAAAGGGTTATACAACATATCACCACAAGCATTCTTGGGCCGAAGCGCCCAGGTGGTCAAAGGTACTG
CCTCAGGATTGAGCGAGCGATTTAATAATTATTTCAATACTAAGTGTGTAAATATTTCATCCTTTTTCATTCGTAGAATCTTTAGGCGTTTGCCAACTTTTGTCACTTTTGTTAACTCAT
TATTAGTTATTAGTATGTTAACTAGCGTAGTGGCAGTGTGTCAGGCAATAATTTTAGATCAGAGGAAGTATAGGAGAGAAATCGAGTTGATGCAGATAGAGAAGAATGAGATTGTCTGCA
TGGAGCTATATGCAAGTTTACAGCGCAAACTTGAACGCGATTTCACATGGGATGAGTACATTGAGTATTTGAAATCAGTAAACCCTCAGATAGTTCAGTTTGCTCAAGCGCAGATGGAAG
AATATGATGTGCGACACCAGCGTTCCACACCAGGTGTTAAAAATTTGGAACAAGTGGTAGCATTTATGGCTTTGGTCATCATGGTGTTCGATGCTGAAAGGAGTGATTGCGTGTTCAAGA
CTCTCAATAAATTTAAGGGTGTCCTTTCCTCAATGGACCATGAAGTTAGACATCAGTCCTTAGACGATGTGATCAAGAATTTTGATGAGAGGAATGAGATTATTGATTTTGAATTGAGTG
AAGACACAATTCGAACATCATCAGTGCTAGATACAAAGTTTAGTGATTGGTGGGACCGACAAATCCAGATGGGACATACACTTCCACATTACAGAACCGAGGGGCACTTCATGGAATTTA
CAAGAGCAACTGCCGTTCAAGTGGCTAATGACATTGCCCATAGCGAACACCTAGACTTCCTAGTAAGGGGAGCTGTTGGGTCTGGAAAGTCAACTGGGTTGCCTGTTCATCTTAGTGTAG
CTGGATCTGTGCTTTTGATTGAACCAACGCGACCACTAGCGGAGAACGTTTTCAAACAGCTATCTAGTGAACCATTCTTCAAGAAGCCAACACTGCGTATGCGTGGAAATAGTATATTTG
GCTCTTCTCCAATCTCCGTCATGACTAGTGGATTTGCGCTACACTACTTTGCCAATAATCGCTCTCAATTAGCTCAGTTCAACTTTGTAATATTTGATGAGTGCCATGTTCTGGATCCTT
CCGCAATGGCGTTCCGCAGTTTGCTGAGTGTTTATCATCAAGCATGCAAAGTATTAAAAGTGTCAGCTACTCCAGTGGGAAGAGAGGTTGAATTCACAACACAGCAGCCAGTCAAGTTAA
TAGTGGAAGACACACTGTCTTTCCAATCATTTGTTGATGCACAAGGTTCTAAAACTAATGCTGATGTTGTTCAGTTTGGTTCAAACGTACTTGTGTACGTGTCGAGCTACAATGAAGTTG
ATACCTTGGCTAAGCTCCTAACAGACAAGAATATGATGGTCACAAAGGTTGATGGCAGAACAATGAAGCACGGTTGCCTAGAAATTGTCACAAAGGAACCAGTGCGAGACCACATTTTG
TTGTAGCAACCAACATAATTGAGAATGGAGTGACTTTGGACATAGACGTGGTTGTAGATTTTGGGTTGAAAGTCTCACCGTTCTTGGACATTGACAATAGGAGCATTGCTTACAATAAGG
TGAGTGTTAGCTATGGTGAGAGAATTCAAAGGCTGGGTCGTGTTGGACGCTTCAAGAAAGGAGTAGCATTGCGCATTGGACACACTGAGAAGGGAATTATTGAAATTCCAAGCATGATCG
CTACAGAGGCGGCTCTTGCTTGTTTTGCATATAACTTGCCAGTGATGACAGGAGGCGTCTCAACTAGTCTGATTGGCAATTGTACTGTGCGCCAAGTTAAAACAATGCAGCAATTTGAAT
TGAGTCCCTTCTTTATCCAGAATTTCGTTGCTCATGATGGATCAATGCATCCTGTCATACATGACATTCTTAAAAAGTATAAACTTCGAGATTGTATGACACCTTTGTGCGATCAGTCTA
TACCATACAGGGCATCGAGCACTTGGTTATCGGTTAGTGAATATGAGCGACTTGGAGTGGCCTTAGAAATTCCAAAGCAAGTCAAAATTGCATTCCACATCAAAGAGATCCCTCCTAAGC
TCCACGAAATGCTTTGGGAAACGGTTGTCAAGTACAAAGATGTTTGCTTATTTCCAAGCATTCGAGCATCGTCCATCAGCAAAATCGCATACACATTGCGTACAGACCTCTTCGCCATCC
CAAGAACTCTAATATTGGTGGAGAGACTGCTTGAAGAGGAGCGAGTGAAGCAGAGCCAATTCAGAAGTCTCATCGACGAAGGATGCTCAAGCATGTTTTCAATTGTCAACTTGACAAACA
CTCTCAGAGCTAGATATGCAAAAGATTACACTGCAGAGAACATACAAAAACTTGAGAAAGTGAGAAGTCAATTGAAAGAATTCTCAAATTTGGATGGTTCTGCATGTGAGGAAAATTTAA
TAAAGAGGTATGAGTCTTTGCAGTTCGTTCATCACCAAGCTGCGACGTCACTTGCAAAGGATCTCAAGTTGAAGGGGACTTGGAAGAAGTCATTAGTGGCCAAAGACTTGATCATAGCAG
GCGCTGTTGCAATTGGTGGAATAGGACTCATATATAGTTGGTTCACACAATCAGTTGAGACTGTGTCTCACCAAGGGAAAAATAAATCCAAAAGAATTCAAGCCTTGAAGTTTCGCCATG
CTCGCGACAAAAGGGCTGGCTTTGAAATTGACAACAATGATGACACAATAGAGGAATTCTTTGGATCTGCATACAGGAAAAAGGGAAAAGGTAAAGGTACCACAGTTGGTATGGGCAAGT
CAAGCAGGAGGTTCATCAACATGTATGGGTTTGATCCAACAGAGTACTCATTCATCCAATTCGTTGATCCACTCACTGGGGCGCAAATAGAAGAGAATGTCTATGCTGACATTAGAGATA
TTCAAGAGAGATTTAGTGAAGTGCGAAAGAAAATGGTTGAGAATGATGACATTGAAATGCAAGCCTTGGGCAGTAACACGACCATACATGCATACTTCAGAAAAGATTGGTCTGACAAAG
CTTTGAAAATTGATTTAATGCCACATAATCCACTCAAAGTTTGTGACAAGACAAATGGCATTGCCAAATTTCCTGAGAGAGAGCTCGAACTAAGGCAGACTGGGCCAGCTGTAGAAGTCG
ACGTGAAGGACATACCAGCACAGGAGGTGGAGCATGAAGCTAAATCGCTCATGAGAGGCTTGAGAGACTTCAACCCAATTGCCCAAACAGTTTGTAGGCTGAAAGTATCTGTTGAATATG
GGACATCAGAGATGTATGGTTTTGGATTTGGAGCATACATAATAGCGAACCACCATTTGTTCAGGAGTTACAATGGTTCCATGGAGGTGCGATCCATGCACGGTACATTCAGGGTGAAGA
ATTTACACAGTTTGAGCGTTCTGCCAATTAAAGGTAGGGATATCATCCTCATCAAAATGCCGAAAGATTTCCCTGTCTTTCCACAGAAATTGCATTTCCGAGCTCCTACACAGAATGAAA
GAGTTTGTTTAGTTGGAACCAACTTTCAGGAGAAGTATGCATCGTCAATCATCACAGAAACAAGCACCACTTACAATATACCAGGCAGCACATTCTGGAAGCATTGGATTGAAACAGATA
ATGGACATTGTGGACTACCAGTGGTGAGCACTACCGATGGATGTCTAGTTGGAATTCACAGTTTGGCAAACAACAAACACACCACGAACTACTACTCAGCCTTCGATGAAGATTTTGAAA
GCAAGTACCTCCGAACCAATGAGCACAATGAATGGGTCAAGTCTTGGATTTATAATCCAGACACAGTGTTGTGGGGCCCGTTGAAACTTAAAGACAGCACTCCCAAAGGATTGTTCAAAA
CAACAAAGCTTGTGCAAGATCTAATCGATCATGATGTAGTGGTGGAGCAAGCTAAGCACTCTGCGTGGATGTTTGAAGCCTTGACAGGAAATTTGCAAGCTGTCGCAACAATGAAGAGCC
AATTAGTAACCAAGCATGTAGTTAAAGGGGAGTGTCGACACTTCAAAGAATTCCTGACTGTGGATGCAGAAGCAGAGGCATTCTTCAGGCCTTTGATGGATGCGTATGGAAAAGCTTGC
TGAATAGAGATGCATACATCAAGGACATAATGAAGTATTCAAAACCTATAGATGTTGGTATCGTGGACTGTGATGCATTTGAGGAAGCCATCAATAGGGTTATCATCTACCTGCAAGTGC
ATGGCTTCAAGAAGTGCGCATACGTCACTGACGAGCAAGAAATTTTCAAAGCGCTCAACATGAAAGCTGCAGTCGGAGCCATGTATGGTGGCAAAAAGAAAGACTATTTTGAGCATTTCA
CTGATGCAGATAAGGAAGAAATAGTCATGCAAAGCTGTCTGCGATTGTATAAAGGCTTGCTTGGCATTTGGAATGGATCATTGAAGGCAGAGCTCCGGTGTAAGGAAAAGATACTTGCAA
ATAAGACGAGGACATTCACTGCTGCACCTTTAGACACTTTGCTGGGTGGTAAAGTGTGTGTTGATGACTTCAATAATCAATTTTATTCAAAGAATATTGAATGCTGTTGGACGGTTGGGA
TGACTAAGTTTTATGGTGGTTGGGATAAACTGCTGCGGCGTTTACCTGAGAATTGGGTATACTGTGATGCCGATGGCTCACAGTTTGATAGTTCACTAACTCCATACTTAATCAATGCTG
TTCTCACCATTAGAAGCACATACATGGAAGATTGGGATGTGGGGTTGCAAATGTTGCGCAATTTATACACTGAAATTGTTTACACACCTATTTCAACTCCAGATGGAACAATTGTTAAGA
AGTTCAGAGGAAATAACAGTGGTCAGCCTTCTACTGTTGTAGACAACTCTCTTATGGTCGTCCTTGCCATGCACTATGCTCTCATCAAAGAATGCATTGAGTTTGAAGAGATTGACAGCA
CGTGCGTGTTCTTTGTTAATGGTGATGATTTGCTGATTGCTGTGAATCCGGATAAAGAGGGCATTCTTGACAGATTGTCACAACACTTCTCAGATCTTGGTTTGAATTATGATTTCTCGT
CAAGAACAAGAAATAAGGAGGAGTTGTGGTTTATGTCTCATAGAGGCCTACTGATTGAGGGCATGTACGTGCCGAAACTTGAAGAAGAAAGGATTGTGTCCATTCTCCAATGGGACAGAG
CAGACTTGGCTGAACATAGGCTTGAGGCGATTTGCGCAGCTATGATAGAGTCCTGGGGTTATTCTGAACTAACACACCAAATCAGGAGATTCTACTCATGGTTATTGCAGCAGCAACCCT
TTGCAACAATAGCGCAGGAAGGGAAGGCTCCTTATATAGCAAGCATGGCATTAAGGAAATTGTATATGGATAGGGCTGTGGATGAGGAAGAGCTGAGAGCCTTCACTGAAATGATGGTCG
CATTAGACGATGAGTTTGAATTTGACTCTTATGAAGTATACCATCAAGCAAATGACACAATCGATGCAGGAGGAAGCAGCAAGAAAGATGCAAGACCAGAGCAAGGCAGCATCCAGTCAA
ACCCGAACAAAGGAAAAGATAAGGATGTGAATGCTGGTACATCTGGGACACATACTGTGCCGAGAATCAAGGCTATCACGTCCAAAATGAGAATGCCCAAAAGCAAGGGAGCAACCGTGC
TAAACTTAGAACACTTGCTTGAGTATGCTCCACAACAAATTGATATTTCAAATACTCGGGCAACTCAATCACAGTTTGATACGTGGTATGAGGCAGTGCGGATGGCATACGACATAGGAG
AAACTGAGATGCCAACTGTGATGAATGGGCTTATGGTTTGGTGCATTGAAAATGGAACCTCGCCAAATGTCAACGGAGTTTGGGTTATGATGGATGGGAATGAACAAGTCGAGTACCCGT
TGAAACCAATCGTTGAGAATGCAAAACCAACCCTTAGGCAAATCATGGCACATTTCTCAGATGTTGCAGAAGCGTATATAGAAATGCGCAACAAAAAGGAACCATATATGCCACGATATG
GTTTAATTCGAAATCTGCGGGATGTGGGTTTAGCGCGTTATGCCTTTGACTTTTATGAGGTCACATCACGAACACCAGTGAGGGCTAGGGAAGCGCACATTCAAATGAAGGCCGCAGCAT
TGAAATCAGCTCAACCTCGACTTTTCGGGTTGGACGGTGGCATCAGTACACAAGAGGAGAACACAGAGAGGCACACCACCGAGGATGTCTCTCCAAGTATGCATACTCTACTTGGAGTCA
AGAACATGTGATGTAGTGTCTCTCCGGACGATATATAAGTATTTACATATGCAGTAAGTATTTTGGCTTTTCCTGTACTACTTTTATCATAATTAATAATCAGTTTGAATATTACTAATA
GATAGAGGTGGCAGGGTGATTTCGTCATTGTGGTGACTCTATCTTTTAATTCCGCATTATTAAGTCTTAGATAAAAGTGCCGGGTTGTCGTTGTTGTGGATGATTCATCGATTAGGTGAT
GTTGCGATTCTGTCGTAGCAGTGACTATGTCTGGATCTATCTGCTTGGGTGGTGTTGTGATTTCGTCATAACAGTGACTGTAAACTTCAATCAGGAGACAAAAAAAAAAAAAAAAAAAA
AAAAAAAAAAAAAAAAAAAAAAAAAAAAAAAAA

>PVY_ALYU-79
aaATTAAAACAACTCAATACAACATAAGAAAAACAACGCAAAAACACTCATAAACGCTTATTCTCACTCAAGCAACTTGCTAAGTTTCAGTTTAAATCATTTCCTTGCAATTCTCTTAAA
CGATATTGGAAACCATTTCAACTCAACAAGTAATTTCATCACTTCCAACCAATTTTAGATCCTCAATGGCAACTTACATGTCAACAATCTGTTTCGGTTCGTTTGAATGCAAGCTACCAT
ACTCACCCGCCTCTTGCGGGCATATTGTGAAGGAACGAGAAGTGCTAGCTTCCGTTGATCCTTTCGCAGATCTGGAAACACAACTTAGTGCACGATTGCTCAAGCAAGAATATGCTACTG
TTCGTGTGCTCAAGAACGGTACTCTTACGTACCGATACAAGACTGATGCCCAGATAACGCGCATCCAAAAGAAACTGGAAAGGAAGGATAGGGAAGAATATCACTTCCAGATGGCAGCTC

CTAGTATTGTGTCAAAGATCACCATTGCTGGTGGAGAGCCACCTTCAAAACTTGAATCACAAGTGCGGAGGGGTGTCATCCACACAACTCCAAGGATGCGCACAGCAAAAACATATCGCA
CGCCAAAGTTGACAGAGGGACAAATGAACCACCTTATCAAGCAGGTGAAGCAAATTATGTCAACCAAAGGAGGGTCTGTTCAACTGATTAGCAAGAAAAGTACCCATGTTCACTATAAAG
AAGTTTTGGGATCACATCGCGCAGTTGTTTGCACTGCACATATGAAAGGTTTACGAAAAAGAGTGGACTTTCGGTGTGATAAATGGACCGTTGTGCGTCTACAGCATCTCGCCAGGACGG
ACAAGTGGAATAACCAAGTTCGTGCTACTGATCTACGCAAGGGCGATAGTGGAGTTATATTGAGTAATACTAATCTCAAAGGAAACTTTGGGAGAAGCTCGGAGGGCCTATTCATAGTGC
GTGGGTCGCACGAAGGAAAAATCTATGATGCACGTTCCAAGGTTACTCAAGGGGTTATGGATTCAATGGTTCAGTTCTCAAGCGCTGAAAGCTTTTGGAAGGGATTGGACGGCAATTGGG
CACAAATGAGATATCCTACAGATCATACATGTGTGGCAGGCTTACCAGTTGAAGACTGCGGCAGAGTTGCAGCGATAATGACACACAGTATTTTACCGTGCTATAAGATAACCTGCCCTA
CCTGTGCCCAACAATATGCCAACTTGCCAGCCAGTGACTTACTTAAGATATTACACAAGCACGCAAGTGATGGTTTAAATCGATTGGGGGCAGACAAAGATCGCTTTGTGCATGTCAAAA
AGTTCTTGACAATCTTAGAGCACTTAACTGAACCGGTTGATCTGAGTCTAGAAATTTTCAATGAAGTATTCAAGTCTATAGGGGAGAAGCAACAATCACCTTTCAAAAACCTGAATATTC
TGAATAATTTCTTTTTGAAAGGAAAGGAAAATACAGCTCGTGAATGGCAGGTGGCTCAATTAAGCTTACTTGAATTGGCAAGATTCCAAAAGAACAGAACGGATAATATCAAGAAAGGAG
ACATCTCGTTCTTTAGGAATAAACTATCTGCCAAAGCAAATTGGAACTTGTATCTGTCATGTGATAACCAGCTGGATAAGAATGCAAACTTCCTGTGGGGACAGAGGGAATATCATGCTA
AGCGATTTTTCTCGAACTATTTCGAGGAAATTGATCCAGCGAAGGGCTATTCAGCATATGAAAATCGTTTGCATCCGAATGGGACAAGAAAACTTGCAATTGGAAACCTAATTGTACCAC
TTGATCTGGCTGAGTTTAGGCGGAAGATGAAAGGCGATTATAAAGACAGCCAGGGGTGAGTAAGAAGTGCACGAGCTCGAAGGATGGAAACTACGTGTATCCCTGTTGTTGCACTACAC
TTGATGATGGCTCAGCTGTTGAATCAACATTTTACCCGCCAACTAAGAAGCACCTCGTAATAGGTAATAGTGGCGACCAAAAGTATGTTGACTTACCAAAAGGGAATTCTGAGATGTTAT
ATATTGCCAAGCAAGGCTTCTGTTACATTAACATTTTCCTCGCAATGTTGATTAACATTAGTGAGGAAGATGCAAAGGATTTCACTAAGAAGGTTCGTGACATGTGTGTGCCAAAGCTTG
GAACCTGGCCAACCATGATGGATCTAGCCACAACTTGTGCTCAAATGAAAATATTCTACCCTGATGTTCATGATGCAGAACTGCCTAGAATACTAGTCGATCACGAAACGCAGACATGCC
ATGTGGTTGACTCGTTTGGCTCACAAACAACTGGGTATCATATTTTGAAAGCATCTAGCGTGTCCCAACTTATTTTGTTTGCTAATGATGAGTTGGAGTCTGACATTAAACATTATAGAG
TTGGTGGTGTTCCTAATGCATGCCCTGAACTTGGGTCCACAATATCACCTTTCAGAGAAGGAGGAGTTATAATGTCTGAGTCGGCAGCGCTGAAACTGCTTTTGAAAGGAGTTTTTAGAC
CTAAGGTGATGAGACAGTTGCTGTTAGATGAGCCTTACCTGTTGATTCTATCAATATTATCCCCTGGCATACTGATGGCTATGTACAATAATGGGATTTTTGAACTTGCGGTAAGGTTGT
GGATTAATGAGAAACAATCCATAGCTATGATAGCATCGCTACTATCAGCTTTAGCCCTACGAGTGTCAGCGGCAGAAACACTCGTCGCACAGAGGATTATAATTGATACTGCAGCTACAG
ATCTCCTTGATGCTACGTGCGATGGGTTCAACCTACATCTAACGTACCCCACTGCGTTGATGGTGTTGCAAGTTGTTAAGAATAGAAATGAATGTGATGATACCCTATTCAAGGCGGGTT
TTCCAAGTTACAACACGAGCGTCGTACAGATCATGGAAAAAAATTATCTAAATCTCTTGAACGATGCTTGGAAAGATTTAACTTGGCGGGAAAAATTATCCGCAACATGGTACTCATACA
GAGCAAAACGCTCTATCACTCGGTACATAAAACCCACAGGAAGGGCAGATTTGAAAGGGTTATACAACATATCACCACAAGCATTCTTGGGCCGAGGCGCCCAGGTGGTCAAAGGCACTG
CCTCAGGATTGAGCGAGCGATTTAATAATTATTTCAATACTAAGTGTGTAAATATTTCATCCTTTTTCATTCGTAGAATCTTTAGGCGTTTGCCAACTTTCGTCACTTTTGTTAACTCAT
TATTAGTTATTAGTATGTTAACCAGCGTAGTGGCAGTGTGTCAGGCAATAATTTTAGATCAGAGGAAGTATAGGAGAGAAATCGAGTTGATGCAGATAGAGAAGAATGAGATCGTCTGCA
TGGAGCTATATGCAAGTTTACAGCGCAAACTTGAACGCGATTTCACATGGGATGAGTACATTGAGTATTTGAAGTCAGTAAACCCTCAGATAGTTCAGTTTGCTCAAGCGCAGATGGAAG
AATATGATGTGCGACACCAACGTTCCACACCAGGTGTTAAAAATTTGGAACAAGTGGTAGCATTTATGGCTTTAGTCATCATGGTGTTCGATGCTGAAAGGAGTGATTGCGTGtTTAAAA
CTCTCAATAAATTTAAGGGTGTCCTTTCCTCACTGGACCATGAAGTTAGACATCAGTCCTTAGACGATGTGATCAAGAATTTTGATGAGAGGAATGAGATTATTGATTTTGAATTGAGTG
AGGACACAATTCGAACATCATCAGTGCTAGATACAAAGTTTAGTGATTGGTGGGACCGACAAATCCAGATGGGACATACACTTCCACATTACAGAACCGAGGGGCACTTCATGGAATTCA
CAAGAGCAACTGCTGTCCAAGTGGCTAATGACATTGCCCATAGCGAACACCTAGACTTTCTAGTAAGGGGAGCTGTTGGGTCTGGAAAGTCAACTGGGTTGCCTGTTCATCTTAGTGTAG
CCGGATCTGTGCTTTTAATTGAACCAACGCGACCACTAGCGGAAAACGTTTTCAAACAGCTATCTAGTGAACCATTCTTCAAGAAGCCAACACTGCGTATGCGTGGAAATAGTATATTTG
GCTCTTCTCCAATCTCCATCATGACTAGCGGATTTGCGCTACACTACTTCGCCAATAATCGCTCTCAATTAGCTCAGTTCAACTTTGTAATATTTGATGAGTGCCATGTTCTGGATCCTT
CCGCAATGGCGTTCCGTAGTCTGCTGAGTGTTTATCATCAAGCATGCAAAGTATTAAAAGTGTCAGCTACTCCAGTGGGAAGAGAGGTTGAATTTACAACACAGCAACCAGTCAAGTTAA
TAGTGGAGGACACACTGTCTTTCCAATCATTTGTTGATGCACAAGGTTCTAAAACTAATGCTGATGTTGTTCAATTTGGTTCAAACGTACTTGTGTACGTGTCGAGCTACAATGAAGTTG
ACACCTTGGCTAAGCTCCTAACAGACAAGAATATGATGGTCACAAAGGTTGATGGCAGAACAATGAAGCACGGTTGCCTAGAAATTGTCACAAAAGGAACCAGTGCGAGACCACATTTTG
TTGTAGCAACCAACATAATTGAGAATGGAGTAACTTTGGACATAGACGTGGTTGTAGATTTTGGGTTGAAAGTCTCACCGTTCTTGGACATTGACAATAGGAGCATTGCTTACAATAAGG
TGAGTGTTAGCTATGGTGAGAGAATTCAAAGGCTGGGTCGTGTTGGACGCTTCAAGAAAGGAGTAGCATTGCGCATTGGACACACTGAGAAGGGAATTATTGAAATTCCAAGCATGATCG
CTACAGAGGCGGCTCTTGCTTGCTTTGCATATAACTTGCCAGTGATGACAGGAGGCGTTTCAACTAGTCTGATTGGTAATTGTACTGTGCGCCAAGTTAAAACAATGCAGCAATTTGAAT
TGAGTCCCTTCTTTATCCAGAATTTCGTTGCCCATGATGGATCAATGCATCCTGTCATACATGACATTCTTAAAAAGTATAAACTTCGAGATTGTATGACACCTTTGTGCGATCAATCTA
TACCATACAGGGCATCGAGCACTTGGTTATCGGTTAGTGAATATGAGCGACTTGGAGTGGCCTTAGAAATTCCAAAGCAAGTCAAAATTGCATTCCATATCAAAGAGATCCCTCCTAAGC
TCCACGAAATGCTTTGGGAAACGGTTGTCAAGTATAAAGACGTTTGCTTATTTCCAAGCATTCGAGCATCGTCCATCAGCAAAATCGCATACACATTGCGTACAGACCTCTTCGCCATCC
CAAGAACTCTAATATTGGTGGAGAGACTGCTTGAAGAAGAGCGAGTGAAGCAGAGCCAATTCAGAAGTCTCATCGACGAAGGATGCTCAAGCATGTTTTCAATTGTCAACTTGACAAACA
CTCTCAGAGCTAGATATGCAAAAGATTACACCGCAGAGAACATACAAAAACTTGAGAAAGTGAGAAGTCAATTGAAAGAATTCTCAAATTTGGATGGTTCTGCATGTGAGGAAAATTTAA
TAAAGAGGTATGAGTCTTTGCAGTTCGTTCATCACCAAGCTGCGACGTCACTTGCAAAGGATCTCAAGTTGAAGGGGACTTGGAGAAGTCATTAGTGGCCAAAGACTTGATCATAGCAG
GCGCTGTTGCAATTGGTGGAATAGGACTTATATATAGTTGGTTCACACAATCAGTTGAGACTGTGTCTCACCAAGGGAAAAATAAATCCAAAAGAATTCAAGCCTTGAAGTTTCGCCATG
CTCGTGACAAAAGGGCTGGCTTTGAAATTGACAACAATGATGACACAATAGAGGAATTCTTTGGATCTGCATACAGGAAAAAGGGAAAAGGTAAAGGTACCACAGTTGGTATGGGCAAGT
CAAGCAGGAGGTTCATCAACATGTATGGGTTTGATCCAACAGAGTACTCATTCATCCAATTCGTTGATCCACTCACTGGGGCGCAAATAGAAGAGAATGTCTATGCTGACATTAGAGATA
TTCAAGAGAGATTTAGTGAGGTGCGAAAGAAAATGGTTGAGAATGATGACATTGAAATGCAAGCCTTGGGCAGTAACACGACCATACATGCATACTTCAGGAAAGATTGGTCTGACAAAG
CTTTGAAGATTGATTTAATGCCACATAATCCACTCAAAGTTTGTGACAAGACAAATGGCATTGCCAAATTTCCTGAGAGAGAGCTTGAACTAAGGCAGACTGGGCCAGCTGTAGAAGTCG
ACGTGAAGGACATACCAACACAGGAGGTGGAGCATGAAGCTAAATCGCTCATGAGAGGCTTGAGAGACTTCAACCCAATTGCCCAAACAGTTTGTAGGCTGAAAGTATCTGTTGAATATG
GAACATCAGAGATGTACGGTTTTGGATTTGGAGCATATATAATAGCGAACCACCATTTGTTCAGGAGTTACAATGGTTCCATGGAGGTGCGATCCATGCACGGTACATTCAGGGTGAAGA
ATCTACACAGTTTGAGTGTTCTGCCAATTAAAGGTAGGGATATTATCCTCATCAAAATGCCGAAAGATTTCCCTGTCTTCCCACAGAAATTGCATTTCCGAGCTCCTACACAGAATGAAA
GAGTTTGTTTAGTTGGAACCAACTTTCAGGAGAAGTATGCATCGTCGATCATCACAGAAACAAGCACCACTTACAATATACCAGGCAGCACATTCTGGAAGCATTGGATTGAAACAGATA
ATGGACATTGTGGACTACCAGTGGTGAGCACCACCGATGGATGTCTAGTCGGAATTCACAGTTTGGCAAACAACAAACACACCACGAACTACTACTCAGCCTTCGATGAAGATTTTGAAA
GCAAGTATCTCCGAACCAATGAGCACAATGAATGGGTCAAGTCTTGGATTTATAATCCAGACACAGTGTTGTGGGGCCCGTTGAAACTTAAAGACAGCACTCCCAAAGGACTATTCAAAA
CAACAAAGCTTGTGCAAGATCTAATCGATCATGATGTAGTGGTGGAGCAAGCTAAGCACTCTGCGTGGATGTTTGAAGCCTTGACAGGAAATTTGCAAGCTGTCGCAACAATGAAGAGCC
AATTAGTAACCAAGCATGTAGTTAAAGGAGAGTGTCGACACTTCAAAGAATTCCTGACTGTGGATGCAGAAGCAGAGGCATTCTTCAGGCCTTTGATGGATGCGTATGGAAAAGCTTGC
TGAATAGAGATGCATACATCAAGGACATAATGAAGTATTCAAAACCTATAGATGTTGGTATCGTGGACTGTGATGCATTTGAGGAAGCCATCAATAGGGTTATCATCTACCTGCAAGTGC
ACGGCTTCAAGAAGTGCGCATACGTCACTGACGAGCAAGAAATTTTCAAAGCGCTCAACATGAAAGCTGCAGTCGGAGCCATGTATGGTGGCAAAAAGAAGGACTATTTTGAGCATTTCA
CTGATGCAGATAAGGAAGAAATAGTCATGCAAAGCTGTCTGCGATTGTATAAAGGCTTGCTTGGCATTTGGAATGGATCATTGAAGGCAGAGCTCCGGTGTAAGGAAAGATACTTGCAA
ATAAGACGAGGACATTCACTGCTGCACCTTTAGACACTTTGCTGGGTGGTAAAGTGTGTGTTGATGACTTCAATAATCAATTTTATTCAAAGAATATTGAATGCTGTTGGACGGTTGGGA
TGACTAAGTTTTATGGTGGTTGGGATAAACTGCTGCGGCGTTTGCCTGAGAATTGGGTATACTGTGATGCCGATGGCTCACAGTTTGATAGTTCACTAACTCCATACTTAATCAATGCTG
TTCTCACCATTAGAAGCACATACATGGAAGATTGGGATGTGGGGTTGCAAATGTTGCGCAATTTATACACTGAGATTGTTTACACACCTATTTCAACTCCAGATGGAACAATTGTTAAGA
AGTTCAGAGGAAATAACAGTGGTCAGCCTTCTACTGTTGTGGACAACTCTCTTATGGTCGTCCTTGCCATGCACTATGCTCTCATCAAAGAATGCATTGAGTTTGAAGAGATTGACAGCA
CGTGCGTATTCTTTGTCAATGGTGATGATTTGCTGATTGCTGTGAATCCGGATAAAGAGGGCATTCTTGACAGATTGTCACAACACTTCTCAGATCTTGGTTTGAATTATGATTTCTCGT
CAAGAACAAGAAATAAGGAGGAATTGTGGTTTATGTCTCATAGAGGCCTACTGATTGAGGGCATGTACGTGCCGAAACTTGAAGAAGAAAGGATTGTGTCCATTCTCCAATGGGACAGAG
CAGACTTGGCTGAACATAGGCTTGAAGCGATTTGCGCAGCTATGATAGAATCCTGGGGTTATTCTGAACTAACACACCAAATCAGGAGATTCTACTCATGGCTATTGCAACAGCAACCCT
TTGCAACAATAGCGCAGGAAGGGAAGGCTCCTTATATAGCAAGCATGGCATTAAGGAAATTGTATATGGATAGGGCTGTGGATGAGGAAGAGCTGAGAGCTTTCACTGAAATGATGGTCG
CATTAGACGATGAGTTTGAATTTGACTCTTATGAAGTATACCATCAAGCAAATGACACAATCGATGCAGGAGGAAGCAGCAAGAAAGATGCAAGACCAGAGCAAGGCAGCATCCAGTCAA
ACCCGAACAAAGGAAAAGATAAGGATGTGAATGCTGGTACATCTGGGACACATACTGTGCCGAGAATCAAGGCTATCACGTCCAAAATGAGAATGCCCAAAAGCAAGGGAGCAACCGTGC
TAAACTTAGAACACTTGCTTGAGTATGCTCCACAACAAATTGATATTTCAAATACTCGGGCAACTCAATCACAGTTTGATACGTGGTATGAGGCAGTGCGGATGGCATACGACATAGGAG
AAACTGAGATGCCAACTGTGATGAATGGGCTTATGGTTTGGTGCATTGAAAATGGAACCTCGCCAAATGTCAACGGAGTTTGGGTTATGATGGATGGGGATGAACAAGTCGAGTACCCGT
TGAAACCAATCGTTGAGAATGCAAAACCAACCCTTAGGCAAATCATGGCACATTTCTCAGATGTTGCAGAAGCGTATATAGAAATGCGCAACAAAAAGGAACCATATATGCCACGATATG
GTTTAATTCGAAATCTGCGGGATGTGGGTTTAGCGCGTTATGCCTTTGACTTTTATGAGGTCACATCACGAACACCAGTGAGGGCTAGGGAAGCGCACATTCAAATGAAGGCCGCAGCAT
TGAAATCAGCCCAACCTCGACTTTTCGGGTTGGACGGTGGCATCAGTACACAAGAGGAGAACACAGAGAGGCACACCACCGAGGATGTCTCTCCAAGTATGCATACTCTACTTGGAGTTA
AGAACATGTGATGTAGTGTCTCTCCGGACGATATATAAGTATTTACATATGCAGTAAGTATTTTGGCTTTTCCTGTACTACTTTTATCATAATTAATAATCAGTTTGAATATTACTAATA
GATAGAGGTGGCAGGGTGATTTCGTCATTGTGGTGACTCTATCTTTTAATTCCGCATTATTAAGTCTTAGATAAAAGTGCCGGGTTGTCGTTGTTGTGGATGATTCATCGATTAGGTGAT
GTTGCGATTCTGTCGTAGCAGTGACTATGTCTGGATCTATCTGCTTGGGTGGTGTTGTGATTTCGTCATAACAGTGACTGTAAACTTCAATCAGGAGACAAAAAAAAAAAAAAAAAAAA
AAAAAAAAAAAAAAAAAAAAAAAAAAAAAAAAaaa

>RNAi_PVS_25K_cassette13.8
AAGCTTGGCGTGCCTGCAGGTCAACATGGTGGAGCACGACACACTTGTCTACTCCAAAAATATCAAAGATACAGTCTCAGAAGACCAAAGGGCAATTGAGACTTTTCAACAAAGGGTAAT
ATCCGGAAACCTCCTCGGATTCCATTGCCCAGCTATCTGTCACTTTATTGTGAAGATAGTGGAAAAGGAAGGTGGCTCCTACAAATGCCATCATTGCGATAAAGGAAAGGCCATCGTTGA
AGATGCCTCTGCCGACAGTGGTCCCAAAGATGGACCCCCACCCACGAGGAGCATCGTGGAAAAAGAAGACGTTCCAACCACGTCTTCAAAGCAAGTGGATTGATGTGATAACATGGTGGA
GCACGACACACTTGTCTACTCCAAAAATATCAAAGATACAGTCTCAGAAGACCAAAGGGCAATTGAGACTTTTCAACAAAGGGTAATATCCGGAAACCTCCTCGGATTCCATTGCCCAGC
TATCTGTCACTTTATTGTGAAGATAGTGGAAAAGGAAGGTGGCTCCTACAAATGCCATCATTGCGATAAAGGAAAGGCCATCGTTGAAGATGCCTCTGCCGACAGTGGTCCCAAAGATGG
ACCCCCACCCACGAGGAGCATCGTGGAAAAAGAAGACGTTCCAACCACGTCTTCAAAGCAAGTGGATTGATGTGATATCTCCACTGACGTAAGGGATGACGCACAATCCCACTATCCTTC
GCAAGACCCTTCCTCTATATAAGGAAGTTCATTTCATTTGGAGAGGACCTCGACTCTAGAGGATCCATGAGGATATTTGATAGCTTAGGTAATCAGCTTAGTAGTATTGAATATATGGAT
GTGTTTTTGCAAATTTTGAATAAATATAAATTTGAGCGTGTTAGTAGTACTTTAAATAAACCAATAGTTGTTCATAGCGTCCCGGGAGCTGGTAAAAGTTCTGCAATCCGGGAGTTGCTT
AAGTTAGATAGTAGGTTTGAGTGCATTACCCGTGGTCGGCCAGACATCCCGAATCTAGAGGGGGCTTTCATTAAGGCTGAACGCAGTGGGGAGAATAAATTGCTGCTGGTTGATGAGTAC
GTAGAAGGGCCGGTCCCAGAGGACGCCTTTGCAATCTTTGCAGATCCACTTCAGAGCACCGCTGTGAGCCCATACCGAGCGCACTTCATCAAAACATTGAGCCATCGTTTCGGCaAGTGT
ACCGCTTCTCTTTTGAGAGATTTGGGTTGGGACGTACAGGCAGAAGGTCAAGACTCAGTTCAAATTGCAGATATCTTCACAGTTGACCCTAGGGATACTATTGTGTACTTCGAGCCAGAA
GTTGGAGAGTTACTGAGGAGCCACGGAGTCGAGGCGAGCTGCATTGGTGAGGTGCGCGGGGCTACTTTtGAACACGTGACCTTCGTCACTTCTGAGAACGgCCCACTGGTTGATAAgGCT
GCTGCTTTTCAATGCTTaACGAGGCACACTAAGAGcTTGCTCATATTGTGCCCTGATGCCACTTACACCGCCGCCTAACCATGGATCCCTACAGGGTAAATTTCTAGTTTTTCTCCTTCA
TTTTCTTGGTTAGGACCCTTTTCTCTTTTTATTTTTTTGAGCTTTGATCTTTCTTTAAACTGATCTATTTTTTAATTGATTGGTTATGGCGCAAATATTACATAGCTTTAACTGATAATC
TGATTcCTTTATTTCGTGTGTCTATGATGATGATGATGTTACAGAACCGTCGACTTAGGCGGCGGTGTAAGTGGCATCAGGGCACAATATGAGCAAgCTCTTAGTGTGCCTCGTtAAGCA

```
TTGAAAAGCAGCAGCcTTATCAACCAGTGGGcCGTTCTCAGAAGTGACGAAGGTCACGTGTTCaAAAGTAGCCCCGCGCACCTCACCAATGCAGCTCGCCTCGACTCCGTGGCTCCTCAG
TAACTCTCCAACTTCTGGCTCGAAGTACACAATAGTATCCCTAGGGTCAACTGTGAAGATATCTGCAATTTGAACTGAGTCTTGACCTTCTGCCTGTACGTCCCAACCCAAATCTCTCAA
AAGAGAAGCGGTACACTtGCCGAAACGATGGCTCAATGTTTTGATGAAGTGCGCTCGGTATGGGCTCACAGCGGTGCTCTGAAGTGGATCTGCAAAGATTGCAAAGGCGTCCTCTGGGAC
CGGCCCTTCTACGTACTCATCAACCAGCAGCAATTTATTCTCCCCACTGCGTTCAGCCTTAATGAAAGCCCCCTCTAGATTCGGGATGTCTGGCCGACCACGGGTAATGCACTCAAACCT
ACTATCTAACTTAAGCAACTCCCGGATTGCAGAACTTTTACCAGCTCCCGGGACGCTATGAACAACTATTGGTTTATTTAAAGTACTACTAACACGCTCAAATTTATATTTATTCAAAAT
TTGCAAAAACACATCCATATATTCAATACTACTAAGCTGATTACCTAAGCTATCAAATATCCTCATGAGCTCGAATTTCCCCGATCGTTCAAACATTTGGCAATAAAGTTTCTTAAGATT
GAATCCTGTTGCCGGTCTTGCGATGATTATCATATAATTTCTGTTGAATTACGTTAAGCATGTAATAATTAACATGTAATGCATGACGTTATTTATGAGATGGGTTTTTATGATTAGAGT
CCCGCAATTATACATTTAATACGCGATAGAAAACAAAATATAGCGCGCAAACTAGGATAAATTATCGCGCGCGGTGTCATCTATGTTACTAGATCGGGAATTC
```

Dataset S1C. Multiple alignments of PVS and PVM 25K and CP coding sequences.

PVS vs PVM

25K ORF

```
PVS MK442089.1       GTATGAGGATGTTTGAGTGTATGAGGATGTTTGATAGCTTAGGTAATCAGCTTAGTAGTA
PVM HM854296.1       ATTTGAAGTGCTTTAATTA----GTG--------TAGCTTAGGTATT--GCT--ATTGTA
PVM AJ437481.1       GTTTGAAGTGCTTTAATTA----GTG--------TAGCTTAGGTATT--GCT--ATTGTA
PVM JN835299.1       GTTTGAAGTGCTTTAATTA----GTG--------TAGCTTAGGTATT--GCT--ATTGTA
PVM AY311394.1       GTTTGAAGTGCTTTAATTA----GTG--------TAGCTTAGGTATT--GCT--ATTGTA
PVM AY311395.1       GTTTGAAGTGCTTTAATTA----GTG--------TAGCTTAGGTATT--GCT--ATTGTA
PVM MH558036.1       ATTTGAAGTGCTTTAATTA----GTG--------TAGCTTAGGTATT--GCT--ATTGTA
PVM MH558037.1       ATTTGAAGTGCTTTAATTA----GTG--------TAGCTTAGGTATT--GCT--ATTGTA
PVM D14449.2         ATTTGAAGTGCTTTAATTA----GTG--------TAGCTTAGGTATT--GCT--ATTGTA
PVM NC_001361.2      ATTTGAAGTGCTTTAATTA----GTG--------TAGCTTAGGTATT--GCT--ATTGTA
PVM JX678982.1       ATTTGAAGTGCTTTAATTA----GTG--------TAGCTTAGGTATT--GCT--ATTGTA
PVM EU604672.1       GTTTGAAGTGCTTTAATTA----GTG--------TAGCTTAGGTATT--GCT--ATTGTA
PVM MW582794.1       GTTTGAAGTGCTTTAATTA----GTG--------TAGCTTAGGTATT--GCT--ATTGTA
PVM MT114149.1       CTTTGAAGTACTTTGATTGGTTAGTG--------TAGCTTAGGTAGT--GCT--ATTGTA
PVM MG356508.1       GTTTGAAGTGCTTTAGATA----AGA--------TAGCTTAGGTAGT--GCT--ATAGGT
PVM MG356509.1       GTTTGAAGTGCTTTAGATA----AGA--------TAGCTTAGGTAGT--GCT--ATAGGT
PVM MF133527.1       GTTTGAAGTGCTTTAGATA----AGG--------TAGCTTAGGTAGT--GCT--ATAGGT
PVM MF133530.1       GTTTGAAGTGCTTTAGATA----AGG--------TAGCTTAGGTAGT--GCT--ATAGGT
PVM MW307264.1       GTTCGAGGTGCTTTAAAGA---GAGG--------TAGCTTAGGTAGT--GCT--GTAGAT
PVM MF133528.1       GTTTGAAGTGCTTTAAATA-----GG--------TAGCTTAGGTAGT--GCT--ATAGGT
PVM MF133529.1       GTTTGAAGTGCTTTAAATA-----GG--------TAGCTTAGGTGGT--GCT--ATAGGT
PVM KY364848.1       ATTTGAAGTGCTTTAAATA-----GG--------TAGCTTAGGTAGT--GCT--ATAGGT
PVM MT114148.1       GTTTGAAGTGCTTTAGATA-----GG--------TAGCTTAGGTAGT--GCT--ATAGGT
                      *  ** *   *** * *      **        *********** *  ***   * ***

PVS MK442089.1       TTGAATA--TATGGATGTGTTTTTGCAAATTTTGAATAAATATAAGTTTGAGCGTGTTAG
PVM HM854296.1       TYGAATATTTATGGAYGTGATCGTAGATTTGTTGTATAAATACAAGTTTGAGCGCTTAAA
PVM AJ437481.1       TTGAATATTTATGGATGTGATTGTAGATTTGTTGTATAAATACAAGTTTGAGCGTTTAAG
PVM JN835299.1       TTGAATATTTATGGATGTGATTGTAGATTTGTTGTATAAATACAAGTTTGAGCGTTTAAA
PVM AY311394.1       TTGAATATTTATGGATGTGATTGTAGATTTGTTGTGTAAATACAAGTTTGAGCGTTTAAG
PVM AY311395.1       TTGAATATTTATGGATGTGATTGTAGATTTGTTGTATAAATACAAGTTTGAGCGTTTAAA
PVM MH558036.1       TTGAATATTTATGGATGTGATTGTAGATTTGTTGCATAAGTACAAGTTTGAGCGTTTAAA
PVM MH558037.1       TTGAATATTTATGGATGTGATTGTAGATTTGTTGCATAAGCACAAGTTTGAGCGTTTAAA
PVM D14449.2         TTGAATATTTATGGATGTGATTGTAGATTTGTTGTATAAATACAAGTTTGAGCGTTTAAG
PVM NC_001361.2      TTGAATATTTATGGATGTGATTGTAGATTTGTTGTATAAATACAAGTTTGAGCGTTTAAG
PVM JX678982.1       TTGAATATTTATGGATGTGATTGTAGATTTGTTGTATAAATACAAGTTTGAGCGTTTAAG
PVM EU604672.1       TTGAATATTTATGGATGTGATTGTAGATTTGTTGTATAAATACAAGTTTGAGCGTCTAAG
PVM MW582794.1       TTGAATATTTATGGATGTGATTGTAGATTTGTTGTATAAATACAAGTTTGAGCGTCTAAG
PVM MT114149.1       TTGAATATTTATGGATGTACTTGTAGATTTATTGCATAAGTATAAGTTTGAGCGTTTGAA
PVM MG356508.1       TTGAATATTTATGGATGTTGTTGTAGAATTGTTGTGTAAGTATAAGTTTGAACGTTTGCA
PVM MG356509.1       TTGAATATTTATGGATGTTGTTGTAGAATTGTTGTGTAAGTATAAGTTTGAACGTTTGCA
PVM MF133527.1       TTGAATATTTATGGATGTTGTTGTAGAATTGTTGTGTAAGTATAAGTTTGAACGTTTGCA
PVM MF133530.1       TTGAATATTTATGGATGTTGTTGTAGAATTGTTGTGTAAGTATAAATTTGAACGTTTGCA
PVM MW307264.1       TTGAATATTTATGGACGTACTTGTAGAATTATTGTATAAATATAAGTTTGAGCGTTTGCA
PVM MF133528.1       TTGAATATTTATGGATGTTGTTGTAGAATTGTTGTGTAAATACAAGTTTGAACGTTTGCA
PVM MF133529.1       TTGAATATTTATGGATGTTGTTGTAGAATTGTTGCGTAAATACAAGTTTGTGCGTTTGCA
PVM KY364848.1       TTGAATATTTATGGATGTAGTCGTAGAGTTGTTGTATAAATACAAGTTTGAACGTTTGCA
PVM MT114148.1       TTGAATATTTATGGATGTTGTTGTAGAACTGTTGTGTAAATACAAGTTTGAACGTTTGCA
                     ******* ********** ** *  *  * *** ******* ************ * *
```

```
PVS MK442089.1       TAGCACTTTAAATAAACCAATAGTTGTTCATAGTGTCCCAGGAGCTGGTAAAAGTTCTGC
PVM HM854296.1       TAATAAGTTAGTGTGCCCTATAGTTGTTCACTGTGTGCCTGGGGCTGGCAAGAGTAGCTT
PVM AJ437481.1       TAATAAATTAGTGTGCCCCATAGTCGTTCACTGCGTGCCTGGGGCTGGCAAGAGTAGCTT
PVM JN835299.1       TAATAAATTAGTGTGCCCCATAGTTGTTCACTGCGTGCCTGGAGCTGGCAAGAGTAGCTT
PVM AY311394.1       TAATAAGTTAGTGTGCCCCATAGTTGTTCACTGCGTGCCTGGAGCTGGTAAGAGTAGTTT
PVM AY311395.1       TAATAAGTTAGTGTGCCCCATAGTTGTTCACTGCGTGCCTGGGGCTGGCAAGAGTAGCTT
PVM MH558036.1       TAACAAGTTAGTGTGCCCTATAGTTGTTCACTGTGTGCCTGGGGCTGGCAAGAGTAGCTT
PVM MH558037.1       TAACAAGTTAGTGTGCCCTATTGTTGTTCACTGTGTGCCTGGGGCTGGCAAGAGTAGCTT
PVM D14449.2         TAATAAGTTAGTGTGCCCTATAGTTGTTCACTGTGTGCCTGGGGCTGGCAAGAGTAGCTT
PVM NC_001361.2      TAATAAGTTAGTGTGCCCTATAGTTGTTCACTGTGTGCCTGGGGCTGGCAAGAGTAGCTT
PVM JX678982.1       TAATAAGTTAGTGTGCCCTATAGTCGTTCACTGTGTGCCTGGGGCTGGCAAGAGTAGCTT
PVM EU604672.1       TAATAAGTTAGTGTACCCTATAGTTGTTCACTGTGTGCCTGGGGCTGGCAAGAGTAGCTT
PVM MW582794.1       TAATAAGTTAGTGTACCCTATAGTTGTTCACTGTGTGCCTGGGGCTGGCAAGAGTAGCTT
PVM MT114149.1       TAATAAATTGACGCAGCCCATTGTTGTGCATTGTGTGCCTGGGGCCGGGAAGAGTAGCCT
PVM MG356508.1       TAGTAAGTTAGAGTTGCCAATAGTTGTGCATTGCGTCCCTGGTGCAGGAAAAAGCAGCTT
PVM MG356509.1       TAGTAAGTTAGAGTTGCCAATAGTTGTGCATTGCGTCCCTGGTGCAGGAAAAAGCAGCTT
PVM MF133527.1       TAGTAAGTTAGAGTTGCCAATAGTTGTGCATTGCGTTCCTGGTGCAGGAAAAAGCAGCTT
PVM MF133530.1       TAGTAAGTTAGAGTTGCCAATAGTTGTGCATTGCGTCCCTGGTGCGGGAAAAAGCAGCTT
PVM MW307264.1       TAACAAGTTAGAGCTTCCAATTGTAGTTCATTGTGTGCCCGGCGCAGGGAAGAGTAGTTT
PVM MF133528.1       TAATAAATTAGCATTCCCAATTGTAGTGCATTGTGTGCCCGGTGCAGGGAAAAGCAGTTT
PVM MF133529.1       TAATAAATTGGCCTTTCCAATTGTAGTGCATTGTGTGCCCGGGGCCGGGAAAAGCAGTTT
PVM KY364848.1       TAATAAATTAGCATTTCCGATTGTAGTGCATTGTGTGCCCGGCGCAGGGAAAAGCAGTTT
PVM MT114148.1       TAACAAATTAGCATTTCCAATTGTAGTGCATTGTGTGCCTGGTGCAGGGAAAAGCAGTTT
                     **  *  ***      ** ***********  **** ** ** ** ** ** ***

PVS MK442089.1       AATTCGGGAGTTGCTTAAGTTAGATAGTAGGTTTGAGTGCAT-TACCCGTGGCCGGCCAG
PVM HM854296.1       AATTCGCGAGTTGTTAGAATTAGATAGTCGCTTCT-GTGCATACACGGCTGGTGTAGAGG
PVM J437481.1        AATTCGCGAGTTGCTAGAATTAGATAGTCGCTTCT-GTGCATACACAGCTGGTGTAGAGG
PVM JN835299.1       AATTCGCGAGTTGCTAGAATTAGATAGTCGCTTCT-GTGCATACACAGCTGGTGTGGAGG
PVM AY311394.1       AATTCGCGAGTTGCTAGAATTAGATAGTCGCTTCT-GTGCATACACAGCTGGTGTAGAGG
PVM AY311395.1       GATTCGCGAGTTGCTAGAATTAGATAGTCGCTTCT-GTGCATACACAGCTGGTGTAGAGG
PVM MH558036.1       AATTCGCGAGTTGTTAGAATTAGACAGTCGCTTCT-GTGCATACACAGCTGGTGTTGAGG
PVM MH558037.1       AATTCGCGAGTTGTTAGAATTAGACAGTCGCTTCT-GTGCATACACAGCTGGTGTTGAGG
PVM D14449.2         AATTCGCGAGTTGTTAGAATTAGATAGTCGCTTCT-GTGCATACACAGCTGGTGTAGAGG
PVM NC_001361.2      AATTCGCGAGTTGTTAGAATTAGATAGTCGCTTCT-GTGCATACACAGCTGGTGTAGAGG
PVM JX678982.1       AATTCGCGAGTTGTTAGAATTAGATAGTCGCTTCT-GTGCATACACAGCTGGTGTAGAGG
PVM EU604672.1       AATTCGCGAGTTGTTAGAATTAGATAGTCGCTTCT-GTGCATACACAGCTGGTGTAGAGG
PVM MW582794.1       AATTCGCGAGTTGTTAGAATTAGATAGTCGCTTCT-GTGCATACACAGCTGGTGTAGAGG
PVM MT114149.1       AATTCGAGAGCTCATTGAATTAGATAGTAGGTTTT-GCGCTTACACAGCTGGGGTGGAGG
PVM MG356508.1       GATCCGTGAATTGCTAGAATTGGACAGCCGATTTT-GTGCATACACCGCGGGCGTGGAAG
PVM MG356509.1       GATCCGTGAATTGCTAGAATTGGACAGCCGATTTT-GTGCATACACCGCGGGCGTGGAAG
PVM MF133527.1       GATCCGTGAATTGCTAGAATTGGACAGCCGATTTT-GTGCATATACCGCGGGCGTGGAAG
PVM MF133530.1       GATCCGTGAATTGCTAGAATTGGACAGCCGATTTT-GTGCATATACCGCGGGCGTGGAAG
PVM MW307264.1       AATTCGTGAGTTGCTTGAATTAGATAGTCGATTTA-GCGCATACACCGCTGGCGTTGAGG
PVM MF133528.1       GATTCGTGAGTTGCTAGAGTTAGATAGCCGTTTTT-GTGCCTACACAGCTGGCGTAGAGG
PVM MF133529.1       GATTCGTGAGTTGCTAGAGTTAGATAGCCGTTTTT-GTGCCTACACAGCCGGCGTAGAGG
PVM KY364848.1       GATTCGTGAGTTGCTAGAGTTAGATAGTCGTTTTT-GTGCCTACACAGCTGGCGTAGAGG
PVM MT114148.1       GATTCGTGAGTTGCTAGAGTTAGATAGTCGTTTTT-GTGCCTACACAGCTGGCGTGGAGG
                     ****** ********  * ********* * **   ******  **   ***       *

PVS MK442089.1       ACATCCCGAATCTAGAGGGGGCTTTCATCAAGGCTGAGCGTGGTGG---------GGAGA
PVM HM854296.1       ACCAACCAAGGTTGAGCGGGARTTGGATCAAG---AAGTGGAGCGGGCAACAACCGGAAG
PVM AJ437481.1       ACCAACCAAGGCTGAGTGGGAATTGGATCAAG---AAGTGGGGCGGGCAACAACCGGAGG
PVM JN835299.1       ACCAACCAAGGCTGAGTGGGGATTGGATAAAG---AAGTGGAGCGGGCAACAACCGGAGG
PVM AY311394.1       ACCAACCAAGGCTGAGTGGGAATTGGATCAAG---AAGTGGGGCGGGCAACAACCAGAGG
PVM AY311395.1       ACCAACCAAGGCTGAGTGGGAATTGGATCAAG---AAGTGGAGCGGGCAACAACCGGAGG
PVM MH558036.1       ATCAACCAAGGTTGAGTGGGAATTGGATCAAG---AAGTGGAGCGGGCAACAACCGGAAG
PVM MH558037.1       ATCAACCAAGGTTGAGTGGGAATTGGATCAAG---AAGTGGAGCGGGCAACAACCGGAAG
PVM D14449.2         ACCAACCAAGGTTGAGCGGGAATTGGATCAGG---AAGTGGAGCGGGCAACAACCGGAAG
PVM NC_001361.2      ACCAACCAAGGTTGAGCGGGAATTGGATCAGG---AAGTGGAGCGGGCAACAACCGGAAG
PVM JX678982.1       ACCAGCCAAGGTTGAGCGGGAATTGGATCAGG---AAGTAGAGCGGGCAACAACCGGAAG
PVM EU604672.1       ACCAACCAAGGTTGAGCGGGAATTGGATCAGG---AAGTGGAGCGGGCAACAACCGGAAG
PVM MW582794.1       ACCAACCAAGGTTGAGCGGGAATTGGATCAGG---AAGTGGAGCGGGCAACAACCGGAAG
PVM MT114149.1       ATCAACCAAGACTCAGCGGCAACTGGATCAGG---AAGTGGGGAGGACAGCAAACAGAGG
PVM MG356508.1       ATCAACCGCGGCTGAGTGGAAATTGGATAAGA---AAGTGGGAGGGCTCTGCGCCTGAAG
PVM MG356509.1       ATCAACCGCGGCTGAGTGGAAATTGGATAAGA---AAGTGGGAGGGCTCTGCGCCTGAAG
PVM MF133527.1       ATCAACCGCGGCTGAGTGGAAATTGGATAAGG---AAGTGGAGGGGCTCTGCGCCTGAAG
PVM MF133530.1       ATCAACCGCGGCTGAGTGGAAATTGGATAAGA---AAGTGGAAGGGCTCTGCGCCAGAAG
PVM MW307264.1       ATCAACCAAGGCTGAGCGGGAACTGGATCAGG---AAGTGGAAGGGTGCTGCAACTGAAG
PVM MF133528.1       ATCAGCCAAGGTTGAGCGGAAACTGGATCAAA---AAATGGAAGGGCGTAGCGGCAGAAG
PVM MF133529.1       ATCAGCCAAGGTTGAGCGGTAACTGGATCAAA---AAGTGGAAGGGCGTCGCCGCAGAAG
PVM KY364848.1       ATCAGCCAAGGTTGAGCGGGAACTGGATCAAA---AAGTGGAAGGGCGTCGCTACTGAAG
PVM MT114148.1       ATCAGCCAAGGTTGAGCGGAAACTGGATCAAA---AAGTGGAAGGGCGTGGCTGCAGAAG
                     *    ** *   *    ***  **  ******    ** *  * **         ***
```

```
PVS MK442089.1      ATAAATTGCTGCTGGTTGATGAGTACATA---------GAAGGGCCGGTCCCAGAGGATG
PVM HM854296.1      GCAAATTTGTGGTTTTGGACGAGTACACYCTGTTGACTGAAGYAC----CTCCG-----G
PVM AJ437481.1      GTAAATTTGTGGTTTTGGACGAGTACGCTTTGTTAACTGAAGTGC----CCCCG-----G
PVM JN835299.1      GTAAACTTGTGGTTTTGGACGAGTACACTTTATTGACTGAAGTGC----CCCCG-----G
PVM AY311394.1      GCAAATTTGTGGTTTTGGACGAGTACACTCTGTTGACTGAAGCGC----CCCCG-----G
PVM AY311395.1      GCAAATTTGTGGTTTTGGACGAGTACACTCTGTTGACTGAAGTGC----CCCCG-----G
PVM MH558036.1      GCAAATTTGTGGTTTTGGATGAGTACACCCTGTTAACTGAAGTGC----CCCCG-----G
PVM MH558037.1      GCAGATTTGTGGTTTTGGATGAGTACACTCTGTTGACTGAAGTGC----CCCCG-----G
PVM D14449.2       GCAAATTTGTGGTTCTGGACGAGTACACTCTGTTGACTGAAGTGC----CTCCG-----G
PVM NC_001361.2    GCAAATTTGTGGTTCTGGACGAGTACACTCTGTTGACTGAAGTGC----CTCCG-----G
PVM JX678982.1      GCAAATTTGTGGTTCTGGACGAGTACACTCTGTTGACTGAAGTGC----CCCCG-----G
PVM EU604672.1      GCAAATTTGTGGTTCTGGACGAGTACACTCTGTTGACCGAAGTGC----CTCCG-----G
PVM MW582794.1      GCAAATTTGTGGTTCTGGACGAGTACACTCTGTTGACCGAAGTGC----CTCCG-----G
PVM MT114149.1      GCAAGTTGCTAGTGATTGACGAGTACACACTGCTGACAGAGGTGC----CTAGA-----G
PVM MG356508.1      GTAAGTATCTAGTTCTAGATGAGTACACGCTGCTAGCTGAGGTAC----CCAGA-----G
PVM MG356509.1      GTAAGTATCTAGTTCTAGATGAGTACACGCTGCTAGCTGAGGTAC----CCAGA-----G
PVM MF133527.1      GTAAGTATCTAGTTCTAGATGAGTACACGCTGCTAGCTGAGGCAC----CCAGA-----G
PVM MF133530.1      GTAAGTATCTAGTCCTTGATGAGTACACGCTGCTAGCTGAGGTAC----CCAGA-----G
PVM MW307264.1      GTAAGTACCTGGTTCTGGACGAGTATACACTACTAACTGAGGTGC----CTGAG-----G
PVM MF133528.1      GTAAGCACTTGATTTTGGACGAGTACACGTTGCTAACAGAAGTGC----CGCAG-----G
PVM MF133529.1      GTAAGCACTTGATTTTGGACGAGTACACGTTGCTAGCAGAAGTGC----CGCAG-----G
PVM KY364848.1      GTAAGCACTTGATTTTGGACGAGTACACGTTGCTAGTTGAAGTGC----CACAG-----G
PVM MT114148.1      GCAAGCACTTGATTTTGGACGAGTACACGTTGCTAGTTGAAGTGC----CGCAG-----G
                      *****  ** *  * ** *******           **** **    * * *     *

PVS MK442089.1      CCTTTGCAATCTTTGCTGATCCACTTCAGAGTACCGCTGTGAGCCCATACCGGGCACACT
PVM HM854296.1      TATTTGCATTGTTCGGTGATCCCATACAATSSAACACAAGCGCCGTCCAGCGTGCTGACT
PVM AJ437481.1      TATTTGCATTATTCGGCGACCCCATACAATCGAACACAAGCGCCGTTCAGCGCGCTGACT
PVM JN835299.1      TATTTGCATTATTCGGCGACCCCATACAATCGAACACAGGCGCCGTTCAGCGCGCTGACT
PVM AY311394.1      TATTTGCATTATTCGGTGACCCCATACAATCGAGCACAAGCGCCGTTCAGCGCGCTGACT
PVM AY311395.1      TATTTGCATTATTCGGTGACCCCATACAATCGAACACAAGCGCCGTTCAGCGCGCTGACT
PVM MH558036.1      TATTTGCATTGTTCGGTGACCCCATACAATCGAACACAAGCGCCGTTCAGCGTGCCGACT
PVM MH558037.1      TATTTGCATTGTTCGGTGACCCCATACAATCGAACACAAGCGCCGTTCAGCGTGCTGACT
PVM D14449.2       TATTTGCATTGTTCGGTGACCCCATACAATCGAACACAAGCGCCGTTCAGCGTGCTGACT
PVM NC_001361.2    TATTTGCATTGTTCGGTGACCCCATACAATCGAACACAAGCGCCGTTCAGCGTGCTGACT
PVM JX678982.1      TATTTGCATTGTTCGGTGACCCCATACAATCGAACACAAGCGCCGTTCAGCGTGCTGACT
PVM EU604672.1      TATTTGCATTGTTCGGTGACCCCATACAATCGAACACGATCGCCGTTCAGCGTGCTGACT
PVM MW582794.1      TATTTGCATTGTTCGGTGACCCCATACAATCGAACACGATCGCCGTTCAGCGTGCTGACT
PVM MT114149.1      CATTCGCACTATTTGGCGATCCCATTCAATCGAGCGCAGGGTCAGTCCAGTCTGCTGATT
PVM MG356508.1      CTTTTGCATTATTTGGTGACCCAATCCAATCCAACGCTGAGTCTGTGAAAAGTGCGGATT
PVM MG356509.1      CTTTTGCATTATTTGGTGACCCAATCCAATCCAACGCTGAGTCTGTGAAAAGTGCGGATT
PVM MF133527.1      CTTTTGCGTTATTTGGTGACCCAATCCAATCCAACGCTGAGTCTGTGAAAAGTGCGGATT
PVM MF133530.1      CTTTTGCGTTATTTGGTGACCCAATTCAATCCAACGCTGGGTCTGTGAAAAGTGCGGATT
PVM MW307264.1      CATTTGCGTTATTTGGTGATCCTATACAGTCTAACGCAGATTCTGTACGGAGTGCCGACT
PVM MF133528.1      CTTTCGCCCTATTTGGTGATCCGATTCAATCTTGCACTGGATCTGTACGGAGCGCCGATT
PVM MF133529.1      CGTTCGCCCTATTTGGTGATCCGATTCAATCCTGCACTGGATCTGTACGGAGCGCCGATT
PVM KY364848.1      CATTCGCATTATTTGGTGATCCAATTCAGTCCTGCACTGGATCTGTACAGAGCGCCGATT
PVM MT114148.1      CATTCGCATTATTTGGTGATCCAATACAGTCCAGCACTGGATCTGTACGGAGCGCCGATT
                      ****** * ** * *** **  * **    * * *      *    * ** **  ***

PVS MK442089.1      TCATCAAAACATTGAGCCATCGCTTTGGCAAGTGTACCGCTTCCCTTTTGAGAGATTTGG
PVM HM854296.1      TTGTGTGCTCAGTGAGTAGAAGATTCGGCAGCGCCACGTGCGGGCTGTTGCGAGAGTTGG
PVM AJ437481.1      TCGTGTGCTCAGTGAGTAGAAGATTCGGCAGTGCCACGTGCGGGCTGTTACGAGAGTTGG
PVM JN835299.1      TCGTGTGCTCAGTGAGTAGAAGATTCGGCAGTGCCACGTGCGGGCTGTTGCGAGAGTTGG
PVM AY311394.1      TCGTGTGCTCAGTGAGTAGAAGATTCGGCAGTGCCACGTGCGGGCTGTTGCGAGAGTTGG
PVM AY311395.1      TTGTGTGCTCAGTGAGTAGAAGATTCGGCAGTGCCACGTGCGGGCTGTTGCGAGAGTTGG
PVM MH558036.1      TTGTGTGTTCAGTGAGTAGAAGATTCGGCAGTGCCACGTGCGGGCTGTTGCGAGAGTTGG
PVM MH558037.1      TTGTGTGTTCAGTGAGTAGAAGATTCGGCAGTGCTACGTGCGGGCTGTTGCGAGAGTTGG
PVM D14449.2       TTGTGTGCTCAGTGAGTAGAAGATTCGGCAGTGCCACGTGCGGGCTGTTACGGGAGTTGG
PVM NC_001361.2    TTGTGTGCTCAGTGAGTAGAAGATTCGGCAGTGCCACGTGCGGGCTGTTACGGGAGTTGG
PVM JX678982.1      TTGTGTGCTCAGTGAGTAGAAGATTCGGCAGTGCCACGTGCGGGCTGTTGCGAGAGTTGG
PVM EU604672.1      TCGTGTGCTCAGTGAGTAGAAGGTTCGGCAGTGCCACGTGCGGGCTGTTGCAAGAGTTGG
PVM MW582794.1      TCGTGTGCTCAGTGAGTAGAAGGTTCGGCAGTGCCACGTGCGGGCTGTTGCAAGAGTTGG
PVM MT114149.1      TTGTGTGCTCGACCAGTAAGAGATTCGGTAGCACTACGTGCGCCTTCTTGAGAGATCTCG
PVM MG356508.1      TTGTGTGCTCGTTTAGTAGGAGATTTGGCAGCGCTACAGGGTCTTTGCTAAGGGAACTAG
PVM MG356509.1      TTGTGTGCTCGTTTAGTAGGAGATTTGGCAGCGCTACAGGGTCTTTGCTAAGGGAACTAG
PVM MF133527.1      TTGTGTGCTCGTTTAGTAGGAGATTTGGCAGTGCTACAGGGTCTTTACTAAGAGAACTAG
PVM MF133530.1      TTGTGTGCTCGTTTAGTAGGAGATTTGGCAGTGCTACAGGATCGTTGCTAAGAGAACTGG
PVM MW307264.1      TCGTGTGCACTTTCAGCAGAAGATTTGGTAGCGCAACGGGGTCTCTATTGAAGGAGCTAG
PVM MF133528.1      TCGTGTGCTCATTCAGCAGAAGATTTGGCAGTGCTACAGGGTCTCTACTGAGAGAGCTAG
PVM MF133529.1      TCGTGTGCTCATTCAGCAGAAGATTTGGTAGTGCTACAGGGTCTCTACTGAGAGAGCTAG
PVM KY364848.1      TCGTGTGTTCATTCAGCAAAAGGTTTGGCAGTGCTACAGGGTCTCTACTGAGAGAGCTAG
PVM MT114148.1      TCGTATGCTCATTCAGCAGGAGGTTTGGCAGTGCTACGGGGTCTCTACTGAGAGAGCTAG
                    *  *     ** ****     * ** ****     **       ** *** **** ****
```

```
PVS MK442089.1        GTTGGGACGTACAGGCAGAAGGTCAAGATTCAGTTCAAATTGCAGATATCTTCACAGTTG
PVM HM854296.1        GTTGGGACGTTCGAAGTGAAAAGGCTGACCTGRTGCAAGTATCTGATATATACACAAAAG
PVM AJ437481.1        GTTGGGACGTTCGAAGTGAAAAGGCAGACCTGGTGCAAGTATCTGATATATACACAAAAG
PVM JN835299.1        GTTGGGACGTTCGAAGTGAAAAGGCAGACCTGGTGCAAGTATCTGATATATACACAAAAG
PVM AY311394.1        GTTGGGACGTTCGAAGTGAAAAGGCTGACCTGGTGCAAGTATCTGATATATATACAAAAG
PVM AY311395.1        GTTGGGACGTTCGAAGTGAGAAGGCTGACCTGGTGCAAGTATCTGATATATATACAAAAG
PVM MH558036.1        GTTGGAACGTTCGAAGTGAAAAGGCCGACTTAGTGCAAGTATCTGATATATACACAAAAG
PVM MH558037.1        GTTGGAACGTTCGAAGTGAAAAGGCTGACTTAGTGCAAGTATCTGATATATACACAAAAG
PVM D14449.2          GCTGGAACGTTCGAAGTGAAAAGGCTGACCTGGTGCAAGTATCTGATATATACACAAAAG
PVM NC_001361.2       GCTGGAACGTTCGAAGTGAAAAGGCTGACCTGGTGCAAGTATCTGATATATACACAAAAG
PVM JX678982.1        GCTGGAACGTTCGAAGTGAAAAGGCTGACCTGGTGCAAGTATCTGACATATACACAAAAG
PVM EU604672.1        GCTGGAACGTTCAAAGTGAAAAGGCTGACCTGGTGCAAGTATCTGATATATACACAAAAG
PVM MW582794.1        GCTGGAACGTTCAAAGTGAAAAAGCTGACCTGGTGCAAGTATCTGATATATACACAAAAG
PVM MT114149.1        GTTGGGACATTTGGAGTTCGGCTCTCGATCTCGTGCAGGTATCTGACATCTACGTGAAGG
PVM MG356508.1        GCTGGGAAATTTTGAGTGAAGGTCCCGATTTGGTGCAAGTATCAGATATCTTTGTCAAGG
PVM MG356509.1        GCTGGGAAATTTTGAGTGAAGGTCCCGATTTGGTGCAAGTATCAGATATCTTTGTCAAGG
PVM MF133527.1        GCTGGGAAATTTTGAGTGAAGGTCCCGATTTGGTGCAAATATCAGATATCTTTGTCAAGG
PVM MF133530.1        GCTGGGAAATTTTGAGTGAAGGCCCCGATTTGGTGCAAATATCAGATATTTTTGTCAAGG
PVM MW307264.1        GCTGGGACATTCAAAGTGAGGGTTCGGACTTGGTGCAGGTGTCCGACATATTCGTGAAGG
PVM MF133528.1        GTTGGGACATTCGGAGCGAGGGACCTGACCTGGTGCAAGTTTCAGACGTCTTTGTTAAAG
PVM MF133529.1        GCTGGGACATTCGGAGCGAGGGACCTGACCTGGTGCAAGTTTCAGACATCTTCGTAAAAG
PVM KY364848.1        GTTGGGACATTCAGAGCGAGGGACCTGATCTGGTGCAGGTATCTGACATCTTCGTGAAAG
PVM MT114148.1        GTTGGGATATCCGGAGCGAGGGGCCTGACTTGGTGCAAGTTTCAGACATCTTCGCGAAAG
                      * ******** *     ***      **    ** *** *  * ***** * ****   *

PVS MK442089.1        ACCCTAGGGATACTATTGTGTACTTCGAGCCGGAAGTTGGAGAGTTGCTGAGGAGCCACG
PVM HM854296.1        AACCTCTGGGTAAAGTTGTGTTTTTCGAGGAGGAAGTGGGTTGCTTGCTGAGATCACACG
PVM AJ437481.1        AACCTTTGGGTAAAGTTGTGTTTTTCGAGGAGGAAGTGGGTTGCTTGCTGAGATCACACG
PVM JN835299.1        AACCTTTGGGTAAAGTTGTGTTTTTCGAGGAGGAAGTGGGTTGCTTGCTGAGATCACACG
PVM AY311394.1        AACCTTTGGGTAAAGTTGTGTTTTTCGAGGAGGAAGTGGGTTGCTTGCTGAGATCACACG
PVM AY311395.1        AACCTTTGGGTAAAGTTGTGTTTTTCGAGGAGGAAGTGGGTTGCTTGCTGAGATCACACG
PVM MH558036.1        AACCTCAGGGTAAAGTCGTGTTCTTCGAGGAGGAAGTGGGTTGCTTGCTGAGATCACACG
PVM MH558037.1        AACCTCAGGGTAAAGTCGTGTTCTTCGAGGAGGAAGTGGGTTGCTTGCTGAGATCACACG
PVM D14449.2          ACCCCCTGGGCAAAGTTGTGTTCTCAGAGGAGGAAGTGGGTTGCTTGCTGAGATCACACG
PVM NC_001361.2       ACCCCCTGGGCAAAGTTGTGTTCTCAGAGGAGGAAGTGGGTTGCTTGCTGAGATCACACG
PVM JX678982.1        AACCCCTGGGCAAAGTTGTGTTCTTCGAGGAGGAAGTGGGTTGCTTGCTGAGATCACACG
PVM EU604672.1        AACCCCTGGGTAAAGTTGTGTTCTTCGAGGAGGAAGTGGGTTGCTTGCTGAGATCACACG
PVM MW582794.1        AACCCCTGGGTAAAGTTGTGTTCTTCGAGGAGGAAGTGGGTTGCTTGCTGAGATCACACG
PVM MT114149.1        ATCCTGAGGGGGTGGTGGTGTACTTTGAAGAGGAGGTTGGTTGCCTGTTGAGGTCACACG
PVM MG356508.1        AACCAGAGGGCGTGGTTGTGTATTTTGAGGAAGAAGTGGGTTGCTTACTAAGAGCACATG
PVM MG356509.1        AACCAGAGGGCGTGGTTGTGTATTTTGAGGAAGAAGTGGGTTGCTTACTAAGAGCACATG
PVM MF133527.1        AACCAGAAGGCGTGGTTGTGTATTTTGAGGAAGAAGTGGGTTGCTTACTAAGAGCACATA
PVM MF133530.1        AACCAGAAGGCGTGGTTGTGTATTTTGAGGAAGAAGTGGGTTGCTTACTAAGAGCACATA
PVM MW307264.1        AACCAGAGGGAGTGGTAGTATACTTTGAGGAAGAAGTGGGCTGTTTACTCAGAGCGCATA
PVM MF133528.1        AGCCAGAGGGAGTGGTCGTATATTTCGAGGAGGAAGTAGGTTGTCTGCTTAAGGCGCATA
PVM MF133529.1        AACCAGAGGGAGTGGTCGTATATTTCGAGGAGGAAGTAGGTTGTCTGCTTAAGACACATA
PVM KY364848.1        AGCCAGAGGGAGTGGTTGTATATTTCGAGGAAGAAGTAGGTTGCCTGCTTAAGGCGCATA
PVM MT114148.1        AACCAGAGGGAGTGGTTGTATATTTCGAGGAGGAGGTAGGTTGTCTGCTTAAGGCGCATA
                      * **   **      ******  ******  ****** **    ********    ****

PVS MK442089.1        GCGTCGAGGCGAGCTGCATTGGTGAGGTGCGCGGGGCCACTTTCGAACACGTAACCTTCG
PVM HM854296.1        GTGTGGAAGCATTGAGCTTGCAGGAAATAACAGGCCAAACTTTTGAAGTGGTAACGTTTG
PVM AJ437481.1        GGGTGGAAGCATTGAGCTTGCAGGAAATAACGGGCCAGACTTTCGAAGTTGTAACGTTCG
PVM JN835299.1        GGGTGGAAGCATTGAGCTTGCAGGAAATAACGGGCCAGACTTTCGAAGTTGTAACGTTCG
PVM AY311394.1        GGGTGGAAGCATTGAGCTTGCAGGAAATAACAGGCCAAACTTTCGAAGTTGTGACGTTCG
PVM AY311395.1        GGGTGGAAGCATTGAGCTTGCAGGAAATAACGGGCCAAACTTTTGAAGTTGTAACGTTCG
PVM MH558036.1        GGGTGGAAGCGTTGAGCTTGCAGGAAATAACAGGCCAAACTTTTGAAGTGGTAACGTTTG
PVM MH558037.1        GGGTGGAAGCGTTGAGCTTGCAGGAAATAACAGGCCAAACTTTTGAAGTGGTAACGTTTG
PVM D14449.2          GGGTGGAAGCATTGAGCTTGCAGGAAATAACAGGCCAAACTTTCGAGGTGGTAACGTTCG
PVM NC_001361.2       GGGTGGAAGCATTGAGCTTGCAGGAAATAACAGGCCAAACTTTCGAGGTGGTAACGTTCG
PVM JX678982.1        GGGTGGAAGCATTGAGCTTGCAGGAAATAACAGGCCAAACTTTCGAGGTGGTAACGTTCG
PVM EU604672.1        GGGTGGAAGCCTTGAGCTTGCAGGAAATAACAGGCCAAACTTTCGAGGTGGTAACTTTCG
PVM MW582794.1        GGGTGGAAGCCTTGAGCTTGCAGGAAATAACAGGCCAAACTTTCGAGGTGGTAACTTTCG
PVM MT114149.1        GAGTAGAAGCCTTTAACTTGAGCGAAATCGTGGGAAAAACGTTCGAGGTGGTTACTTTCG
PVM MG356508.1        GCGTGGAAGCTTTCAGCCTGAGCGAAATTGTGGGACAGACGTTTGAAGTGGTCACATTTG
PVM MG356509.1        GCGTGGAAGCTTTCAGCCTGAGCGAAATTGTGGGACAGACGTTTGAAGTGGTCACATTTG
PVM MF133527.1        GCGTGGAAGCTTTCAGCCTGAGCGAAATTGTGGGACAGACGTTTGAAGTGGTCACATTTG
PVM MF133530.1        GCGTGGAAGCTTTCAGCCTGAGCGAAATTGTGGGACAGACGTTTGAAGTGGTCACATTTG
PVM MW307264.1        GTGTGGAAGCTTTCAATCTGAGCGAAATTGTGGGTCAGACATTCGAAGTAGTTACTTTTG
PVM MF133528.1        GTGTGGAGGCATTTAGCTTGAGTGAAATCGTGGGCCAAACGTTTGAAGTTGTCACATTTG
PVM MF133529.1        GTGTGGAGGCATTCAGCTTGAGTGAAATCGTGGGCCAAACGTTTGAAGTTGTCACATTTG
PVM KY364848.1        GTGTGGAGGCATTCAGCTTGAGTGAAATCGTGGGCCAAACATTTGAAGTTGTCACGTTTG
PVM MT114148.1        GTGTGGAGGCATTCAGCTTGAGTGAAATCGTGGGCCAAACGTTTGAAGTTGTTACATTTG
                      * ** ** **     ** *    **  *    **    ***** ***   ***** ****
```

```
PVS MK442089.1        TCACTTCTGAGAACAGCCCACTGGTTGATAAGGCTGCTGCTTTTCAGTGCTTGACGAGGC
PVM HM854296.1        TGACTTCAGAGAGTTCTCCAGTGGTTAATCGGGCGGCTGCCTATCAGTGCATGACCAGGC
PVM AJ437481.1        TGACTTCAGAGAATTCTCCGGTGGTTAATCGAGCTGCTGCCTATCAGTGCATGACAGGGC
PVM JN835299.1        TGACTTCAGAGAATTCTCCGGTGGTTAATCGAGCTGCTGCCTATCAGTGCATGACAAGGC
PVM AY311394.1        TGACTTCAGAGAATTCTCCGGTGATTAATCGAGCTGCTGCCTATCAGTGCATGACAAGGC
PVM AY311395.1        TAACTTCAGAGAATTCTCCGGTGATTAATCGAGCTGCTGCCTATCAGTGCATGACAAGGC
PVM MH558036.1        TGACTTCAGAGAATTCACCAGTGATTAATCGAGCGGCTGCTTATCAGTGCATGACAAGGC
PVM MH558037.1        TGACTTCAGAGAATTCTCCAGTGATTAATCGGGCGGCTGCTTACCAGTGCATGACAAGGC
PVM D14449.2          TGACTTCAGAGAATTCTCCAGTGATCAATCGAGCGGCTGCCTATCAGTGCATGACAAGGC
PVM NC_001361.2       TGACTTCAGAGAATTCTCCAGTGATCAATCGAGCGGCTGCCTATCAGTGCATGACAAGGC
PVM JX678982.1        TGACTTCAGAGAATTCTCCAGTGATCAATCGAGCGGCTGCCTATCAGTGCATGACAAGGC
PVM EU604672.1        TGACTTCAGAGAATTCTCCAGTGATCAATCGAGCGGCTGCCTATCAGTGCATGACAAGGC
PVM MW582794.1        TGACTTCAGAGAATTCTCCAGTGATCAATCGAGCGGCTGCCTATCAGTGCATGACAAGGC
PVM MT114149.1        TGACTTCCGAGAATTCACCGATAATCAATAAGGCTGCTGCATATCAGTGCATGACTAGGC
PVM MG356508.1        TGACTTCGGAAAACTCCCCCCAGATCAATCGCGCCGCGGCTTACCAGTGCATGACAAGAC
PVM MG356509.1        TGACTTCGGAAAACTCCCCCCAGATCAATCGCGCCGCGGCTTACCAGTGCATGACAAGAC
PVM MF133527.1        TGACTTCGGAAAACTCCCCCCAGATCAATCGCGCCGCGGCTTACCAGTGCATGACAAGAC
PVM MF133530.1        TGACTTCGGAAAACTCCCCCCAGATCAATCGCGCCGCGGCTTACCAGTGCATGACAAGAC
PVM MW307264.1        TAACGTCGGAGAACTCTCCCCGGATCAATCGCGCCGCTGCTTACCAGTGCATGACAAGGC
PVM MF133528.1        TGACTTCTGAAAACTCGCCAGTAATCAATCGTGCAGCTGCTTACCAGTGCATGACAAGAC
PVM MF133529.1        TGACTTCTGAAAACTCGCCAGTAATTAATCGTGCAGCTGCTTATCAGTGTATGACAAGGC
PVM KY364848.1        TGACCTCAGAAAACTCGCCAGTAATCAACCGTGCAGCTGCTTATCAGTGTATGACAAGGC
PVM MT114148.1        TAACCTCAGAAAACTCGCCAGTAATCAACCGTGCAGCTGCTTATCAGTGTATGACAAGGC
                      * ***** ****     *** ** *  **   ** ******* ******* **** ****
```

```
PVS MK442089.1        ACACTAAGAGCTTGCTCATATTGTGCCCTGATGCCACTTACACCGCCGCCTAACTACACA
PVM HM854296.1        ATCGAACGGCTCTGTACATTTTGTGTCCTGATGCCACTTACACCTCCGCCTGACTTCACA
PVM AJ437481.1        ATCGAACGGCTCTGTACATCTTGTGTCCTGATGCCACTTGCACCGCCGCCTGACTTCACA
PVM JN835299.1        ATCGAACGGCTCTGTACATCTTGTGTCCTGATGCCACTTACACCGCCGCCTGACTTCACA
PVM AY311394.1        ACCGAACAGCTCTGCACATCTTGTGTCCTGATGCCACTTACACCGCCGCCTGACTTCACA
PVM AY311395.1        ATCGAACAGCTCTGCACATCTTGTGTCCTGATGCCACTTACACCGCCGCCTGACTTCACA
PVM MH558036.1        ATCGAACGGCTCTGCACATCTTGTGTCCTGATGCCACTTACACCGCCGCCTGACTTCACA
PVM MH558037.1        ATCGAACGGCTCTGCACATCTTATGTCCTGATGCCACTTACACCGCCGCCTGACTTCACA
PVM D14449.2          ATCGAACGGCTCTGCACATCTTGTGTCCTGATGCCACTTACACCGCCGCCTGACTTCACA
PVM NC_001361.2       ATCGAACGGCTCTGCACATCTTGTGTCCTGATGCCACTTACACCGCCGCCTGACTTCACA
PVM JX678982.1        ATCGAACGGCTCTGCACATCTTGTGTCCTGATGCCACTTACACCGCCGCCTGATTTCACA
PVM EU604672.1        ATCGAACGGCTCTGTACATCTTGTGTCCTGATGCCACTTACACCGCCGCCTGACTTCACA
PVM MW582794.1        ATCGAACGGCTCTGTACATCTTGTGTCCTGATGCCACTTACACCGCCGCCTGACTTCACA
PVM MT114149.1        ACAGAGTGGCACTTCACATATTGTGTCCTAATGCCACTTACACCTCCGCCTGATTTCACC
PVM MG356508.1        ATCGAGTGGCGCTCCATATCCTATGTCCTGATGCCACTTACACCGCCGCCTGATTTCTCA
PVM MG356509.1        ATCGAGTGGCGCTCCATATCCTATGTCCTGATGCCACTTACACCGCCGCCTGATTTCTCA
PVM MF133527.1        ATCGAGTAGCGCTCCATATCCTATGTCCTGATGCCACTTACTCCGCCGCCTGATTTCTCA
PVM MF133530.1        ATCGAGTAGCGCTCCATATCCTATGTCCTGATGCCACTTACTCCGCCGCCTGATTTCTCA
PVM MW307264.1        ATAGAGTTGCTCTTCACATCTTGTGCCCTGATGCCACTTACACCGCCGCCTGATTTTTCA
PVM MF133528.1        ATCGAGTGGCTCTTCACATCTTGTGCCCTAATGCCACTTACTCCGCCGCCTGACTTCTCA
PVM MF133529.1        ATCGAGTGGCTCTTCACATCTTGTGCCCTAATGCCACTTACTCCGCCGCCTGACTTCTCA
PVM KY364848.1        ATCGAGTAGCTCTTCACATTTTGTGCCCTAATGCCACTTACTCCGCCGCCTGATTTCTCA
PVM MT114148.1        ATCGAGTAGCCCTTCACATCTTGTGCCCTAATGCCACTTACTCCGCCGCCTGATTTCTCA
                      *    * *    *** *** ***** ************************* * *   *
```

CP ORF

```
PVS MK442089.1        GCACCTTTAGGTTCACAGGT--------AAGAGTTCGAAGAAACTGTCCCACAGAGAAAA
PVM HM854296.1        GTTCCTTTAGGTCATAAGGTAAATCTGAAATAGTGAGTATGGGAGATTCAACGAGGAAA-
PVM AJ437481.1        GTTCCTTTAGGTCATAAGGTAAATCTGAAATAGTGAGTATGGGTGATTCAACAAAGAAA-
PVM JN835299.1        GTTCCTTTAGGTCATAAGGTAAATCTGAAATAGTGAGTATGGGTGATTCAACAAAGAAA-
PVM AY311394.1        GTTCCTTTAGGTCGTAAGGTAAATCTGAAATAGTGAGTATGGGTGATTCAACGAAGAAA-
PVM AY311395.1        GTTCCTTTAGGTCATAAGGTAAATCTGAAATAGTGAGTATGGGTGATTCAACGAAGAAA-
PVM MH558036.1        GTTCCTTTAGGTCATAAGGTGAATCTGAAATAGTGAGTATGGGAGATTCAACGAAGAAA-
PVM MH558037.1        GTTCCTTTAGGTCATAAGGTGAATCTGAAATAGTGAGTATGGGAGATTCAACGAAGAAA-
PVM D14449.2          GTTCCTTTAGGTCATAAGGTGAATCTGAAATAGTGAGTATGGGAGATTCAACGAAGAAA-
PVM NC_001361.2       GTTCCTTTAGGTCATAAGGTGAATCTGAAATAGTGAGTATGGGAGATTCAACGAAGAAA-
PVM JX678982.1        GTTCCTTTAGGTCATAAGGTGAATCTGAAATAGTGAGTATGGGAGATTCAACGAAGAAA-
PVM EU604672.1        GTTCCTTTAGGTCATAAGGTGAATCTGAAATAGTGAGTATGGGAGATTCAACGAAGAAA-
PVM MW582794.1        GTTCCTTTAGGTCATAAGGTGAATCTGAAATAGTGAGTATGGGAGATTCAACGAAGAAA-
PVM MT114149.1        CTTCCTTTAGGTCATAAGGTTAATCCGATATAGTGAGCATGGGAGATTCAACAAAGAAA-
PVM MG356508.1        CTTCCTTTAGGTCTTAAGGGTATTCTGATATAGTGAGTATGGGCGATCCAACGAAGAAA-
PVM MG356509.1        CTTCCTTTAGGTCTTAAGGGTATTCTGATATAGTGAGTATGGGCGATCCAACGAAGAAA-
PVM MF133527.1        CTTCCTTTAGGTCTTAAGGGTAATCCGATATAGTGAGTATGGGCGATCCAACGAAGAAA-
PVM MF133530.1        CTTCCTTTAGGTCTTAAGGGTAATCCGATATAGTGAGTATGGGCGATCCGACGAAGAAA-
PVM MW307264.1        CTTCCTTTAGGTCTTGAGGGTGATTTGAAATAGTGAGTATGGGCGATCCATTGAAGAAA-
PVM MF133528.1        CTTCCTTTAGGTCTAGAGGGTGATTTGAAATAGTGAGTATGGGAGATTCAATGGAGAAA-
PVM MF133529.1        CTTCCTTTAGGTCTAGAGGGTGATTTGAAATAGTGAGTATGGGAGATCCAATGGAGAAA-
PVM KY364848.1        CTTCCTTTAGGTCTAGAGGGTGATTCGAAATAGTGAGTATGGGGGATCCTATGGAGAAA-
PVM MT114148.1        CTTCCTTTAGGTCTAGAGGGTGATTTGAAATAGTGAGTATGGGGGATCCAATGGAAAAA-
                         *********    ***          * ***  * *       * *       ***
```

```
PVS MK442089.1        TGCCGCCCAAACCGGATCCAACAAGCTCAGGAGAGACACCACAAGCAATACCGCTTGCGC
PVM HM854296.1        ----GCTGAAACTG---CCAAAGAC----GTGGGCACTTCGCAAGAAAAGAGAGAAGCGC
PVM AJ437481.1        ----GCTGAAACTG---CCAAAGAT----GTGGGCACTTCGCATGAGAAGAGAGAAGTGC
PVM JN835299.1        ----GCTGAAACTG---CCAAAGAT----GTGGGCACTTCGCATGAGAAGAGAGAAGCGC
PVM AY311394.1        ----GCTGAAACTG---CCAAAGAC----GTGGGCACTTCGCATGAGAAGAGAGAAGCGC
PVM AY311395.1        ----GCTGAAACTG---CCAAAGAT----GTGGGCACTTCGCATGAGAAGAGAGAAGCGC
PVM MH558036.1        ----GCTGAAACTG---CCAAAGAT----GCGAGCACTTCGCAAGAGAGGAGAGAAGCGC
PVM MH558037.1        ----GCTGAAACTG---CCAAAGAT----GCGAGCACTTCGCATGAGAAGAGAGAAGCGC
PVM D14449.2          ----GCTGAAACTG---CCAAAGAT----GAGGGCACTTCGCAAGAAAGGAGAGAAGCGC
PVM NC_001361.2       ----GCTGAAACTG---CCAAAGAT----GAGGGCACTTCGCAAGAAAGGAGAGAAGCGC
PVM JX678982.1        ----GCTGAAACTG---CCAAAGAT----GTGGGCACTTCGCAAGAAAAGAGAGAAGCGC
PVM EU604672.1        ----GCTGAAACTG---CCAAAGAT----GTGGGCACTTCGCAAGAAAAGAGAGAAGCGC
PVM MW582794.1        ----GCTGAAACTG---CCAAAGAT----GTGGGCACTTCGCAAGAAAGGAGAGAAGCGC
PVM MT114149.1        ----ATTGAAGTCG---CCAAAGAG----GCAGGAACATCGCAAGCGGCCAAAGGAAACA
PVM PVM MG356508.1    ----GCTGAAGCAA---GCAAGGAG----GCGAGCGCTTCCCAAGTAATTCTGGGTCAGA
PVM MG356509.1        ----GCTGAAGCAA---GCAAGGAG----GCGAGCGCTTCCCAAGTAATTCTGGGTCAGA
PVM MF133527.1        ----ACTGAAGCAA---GCAAAGAG----GCGAGCGCTTCCCAAGTAATTCTGGGTCAGA
PVM MF133530.1        ----GCTGAAGCAA---GCGGAGAG----GCGAGCGCTTCCCAAGTAATTCTGGGTCAGA
PVM MW307264.1        ----GCTGAGGCTA---ACAAAGAG----GTGGGCACATCCCAACCAATCCAGGGATCGA
PVM MF133528.1        ----GTTGAAGCTA---GCAAGAAA----GCGAGCACGTCGCAGCCGGCTCAGGGCGCTA
PVM MF133529.1        ----GTCGAAGCTA---GCAAGAAA----GCGAGCACATCGCAGCCGGCTCAGGGTGCTC
PVM KY364848.1        ----GTTGAAGCTA---GCAAGAAG----GCGAGTACATCGCAGCCGGCTCAGGGCGCAA
PVM MT114148.1        ----GTTGAAGCTA---GCAAGAAA----GCGAGCACAGCGCAGCCGGCTCAGGGTGCTA
                          **  **** *   ****  *     *  *  **  * **** **        ****

PVS MK442089.1        CGCCGCCCCGGAACGTAGAGGAGCATA--GAGTTGGCCCAAGTCAAGGGCACGGGCAG--
PVM HM854296.1        GCCCACTGCCAACTGCTGCTGACTTCGAGGGGAAGGACATATCGGAAAGCACTGATGGGC
PVM AJ437481.1        GACCACTACCAACTGCAGCTGATTTCGAGGGGAGGGACACATCGGAGAACACTGATGGAA
PVM JN835299.1        GACCACTGCCAACTGCAGCTGATTTCGAGGGGAGGGACACATCGGAGAACACTGATGGAA
PVM AY311394.1        GACCACTGCCAACTGCAGCTGATTTCGAGGGGAGGGACACATCGGAGAACACTGATGGGA
PVM AY311395.1        GACCACTGCCAACTGCAGCTGATTTCGAGGGGAGGGACACATCGGAGAACACTGATGGGA
PVM MH558036.1        GACCACTGCCAACTGCTGCTGACTTCGAGGGGAGGGACACATCGGAGGACACTGATGGGC
PVM MH558037.1        GACCACTGCCTACTGCTGCTGACTTCGAGGGGAGGAACACATCGGAGGACACTGATGGGC
PVM D14449.2          GACCACTGCCGACTGCTGCTGACTTTGAGGGGAAGGACACATCGGAGAACACTGATGGGC
PVM NC_001361.2       GACCACTGCCGACTGCTGCTGACTTTGAGGGGAAGGACACATCGGAGAACACTGATGGGC
PVM JX678982.1        GACCACTGCCGACTGCTGCTGACTTTGAGGGGAAGGACACATCGGAGAACACTGATGGGC
PVM EU604672.1        GACCATTGCCGACTGCTGCCGACTTTGAGGGGAAGGACACATCGGAGAACACTGATGGGC
PVM MW582794.1        GACCATTGCCGACTGCTGCCGACTTTGAGGGGAAGGACACATCGGCGAACACTGATGGGC
PVM MT114149.1        GACCGCTACCTACAGCGGCGGAATTTGAGGGTGATGATAACTCTGGAGATGCTAGTGTCC
PVM MG356508.1        GGCCACTGCCGAAAGTTACTGACTTTGATGAGGTCGATGATCGAGAAGGAGCCAACGTAC
PVM MG356509.1        GGCCACTGCCGAAAGTTACTGACTTTGATGAGGTCGATGATCGAGAAGGAGCCAACGTAC
PVM MF133527.1        GGCCACTGCCGAAAGCTACTGACTTTGATGAGGTCGATGATCGAGAAGGAGCCAATGTAC
PVM MF133530.1        GGCCAATGCCGAAAGCTACTGACTTTGATGAGGTCGATGATCGAGAAAGAGCCAATGTGC
PVM MW307264.1        GGCCGCTGCCGCGAGCAGCAGATTTCGATGTAACAGAGGATCCGGGGGACCCCAACGCTC
PVM MF133528.1        GGCCGCTGCCAAAGGCCGCGGATTTTGATGTGACTGAAGATCGCGATGGCGCCAATGCTC
PVM MF133529.1        GACCGTTGCCAAAGGCCACGGATTTTGATGTGACCGAAGATCGCGATGGTGCCAATGCTC
PVM KY364848.1        GACCGCTGCCAAAGGCCACGGATTTTGATGTGACTGAAGATCGCGATGGCGCAGATGCTC
PVM MT114148.1        GGCCACTGCCAAAGGCCACGGATTTTGATGCAATTGAGGATAGCGATGGTGCTGATGCTC
                        ** *  *  *  *  *  **   *   * *   * *  *    *** *** *

PVS MK442089.1        --------------AATGAAGAAGCTATGCTGGAGCAGAGGCTCATCAGATTGATTGAAC
PVM HM854296.1        GTGCCACGGATGCTGATGGAGAGATGTCATTGGAGCGGAGGCTTGACAGCCTCCGCGAAT
PVM AJ437481.1        GTACTGCAGATGCTGATGGAGAGATGTCACTGGAGCGGAGGCTTGATAGTCTCCGAGAAT
PVM JN835299.1        GTACTGCAGATGCTGATGGAGAGATGTCACTGGAGCGGAGGCTTGATAGTCTCCGAGAAT
PVM AY311394.1        GTACTGCAGATGCTGATGGAGAGATGTCATTGGAGCGGAGGCTTGATAGTCTCCGAGAAT
PVM AY311395.1        GTGCTGCAGATGCTGATGGAGAGATGTCATTGGAGCGGAGGCTTGATAGTCTTCGAGAAT
PVM MH558036.1        GTGCTGCAGATGCTGATGGAGAGATGTCATTGGAACGGAGGCTTGACAGTCTCCGAGAAT
PVM MH558037.1        GTGCTGCAGATGCTGATGGAGAGATGTCATTGGAACGGAGGCTTGACAGCCTCCGAGAAT
PVM D14449.2          GTGCTGCAGATGCTGATGGAGAAATGTCATTGGAGCGGAGGCTTGACAGCCTCCGAGAAT
PVM NC_001361.2       GTGCTGCAGATGCTGATGGAGAAATGTCATTGGAGCGGAGGCTTGACAGCCTCCGAGAAT
PVM JX678982.1        GTGCTGCAGATGCTGATGGAGAGATGTCATTGGAGCGGAGGCTTGACAGCCTCCGGGAAT
PVM EU604672.1        GTGCTGCAGATGTTGATGGAGAGATGTCATTGGAGCGGAGGCTTGACAGCCTCCGAGAAT
PVM MW582794.1        GTGCTGCAGATGTTGATGGAGAGATGTCATTGGAGCGGAGGCTTGACAGCCTCCGAGAAT
PVM MT114149.1        GCGACGCTGAGGTGAATGAGGAAGCGTCGCTGGAGCGCAGATTGAACAGCTTGCGTGAAT
PVM MG356508.1        GCAATGCTACAAATGAAGAAGAGTCATCTTTGGAACGTAGGCTCGACAGCCTACGCGACT
PVM MG356509.1        GCAATGCTACAAATGAAGAAGAGTCATCTTTGGAACGTAGGCTCGACAGCCTACGCGACT
PVM MF133527.1        GCAATGCTACAAATGAAGAAGAGTCATCTTTGGAACGTAGGCTAGACAGCCTACGCGACT
PVM MF133530.1        GCAATGCTACAAATGAAGAAGAGTCATCTTTGGAACGTAGGCTGGACAGCCTACGCGACT
PVM MW307264.1        GCAATGCTGCAAATGATGAAGAAGCGTCCTTGGAGCGTAGGTTGGACAGTTTGCGTGATT
PVM MF133528.1        ACAATGCTGCAAACGAGGAAGAATCCTCCCTGGAGCGTAGGTTGGACAGCTTGCGTGACT
PVM MF133529.1        ACAATGCTGCGAACGAGGAGGAATCCTCCCTGGAGCGTAGGTTAGACAGCTTGCGTGACT
PVM KY364848.1        ACAATGCTGCGGACGAGGAAGAATCCTCCCTGGAACGTAGGTTGGACAGCTTACGTGACT
PVM MT114148.1        ACAATGCTGCGAACGAGGAAGAATCCTCCCTGGAACGACGGTTGGACAGCTTACGTGACT
                                     *** ***        ****** ******   ***  *    ***
```

```
PVS MK442089.1        TCAT--GGCCTCGAAAAGGCACAATTCAACATTGAGCAACATCTCTTTTGAGATAGGTAG
PVM HM854296.1        TCCTGCGGGAGCGGAGGGGTGCAATTCG--CGTGACAAACCCAGGGTTAGAGACTGGCAG
PVM AJ437481.1        TCCTGCGAGAGCGGAGGGGCGCAATCCG--AGTGACAAACCCAGGGCTAGAGACTGGCAG
PVM JN835299.1        TCCTGCGAGAGCGGAGGGGCGCAATCCG--AGTGACAAACCCAGGGTTAGAAACTGGCAG
PVM AY311394.1        TCCTGCGAGAGCGGAGGGGCGCAATCCG--AGTGACAAACCCCGGGCTAGAGACTGGCAG
PVM AY311395.1        TCCTGCGAGAGCGGAGGGGCGCAATCCG--AGTGACAAACCCGGGGCTAGAGACTGGCAG
PVM MH558036.1        TCCTGCGAGAGCGGAGGGGCGCAATTCG--AGTAACAAACCCGGGGTTAGAGACTGGCAG
PVM MH558037.1        TCCTGCGGGAGCGGAGGGGCGCAATTCG--AGTAACAAACCCGGGGTTAGAGACTGGCAG
PVM D14449.2          TCCTGCGAGAGCGGAGGGGCGCAATTCG--AGTGACAAACCCAGGGTTAGAGACTGGCAG
PVM NC_001361.2       TCCTGCGAGAGCGGAGGGGCGCAATTCG--AGTGACAAACCCAGGGTTAGAGACTGGCAG
PVM JX678982.1        TCCTGCGAGAGCGGAGGGGTGCAATTCG--AGTCACAAACCCGGGGTTAGAGACTGGCAG
PVM EU604672.1        TCCTGCGAGAGCGGAGGGGCGCAATTCG--AGTGACAAACCCGGGGTTAGAGACTGGCAG
PVM MW582794.1        TCCTGCGAGAGCGGAGGGGCGCAATTCG--AGTGACAAACCCGGGGTTAGAGACTGGCAG
PVM MT114149.1        TTCTACGTGAGCGAAGAGGCGCCATACG--GGTTACTAATCCTGGTTTGGAGACTGGCCG
PVM MG356508.1        TTCTGCGCGAAAGACGTGGTGCAATCCG--CGTGACAAACCCAGGTCTAGAGACTGGCAG
PVM MG356509.1        TTCTGCGCGAAAGACGTGGTGCAATCCG--CGTGACAAACCCAGGTCTAGAGACTGGCAG
PVM MF133527.1        TTCTGCGCGAAAGACGTGGCGCAATCCG--CGTGACAAACCCAGGTCTAGAGACTGGCAG
PVM MF133530.1        TTCTGCGCGAAAGACGTGGCGCAATCCG--CGTGACAAACCCAGGTCTAGAGACTGGCAG
PVM MW307264.1        TCTTGCGTGAGAGGCGCGGTGCAATACG--CGTGACAAACCCTGGCCTGGAAACAGGCAA
PVM MF133528.1        TTCTGCGTGAGAGGCGTGGAGCAATTCG--AGTGACTAATCCAGGTCTGGAAACAGGCAG
PVM MF133529.1        TTCTGCGTGAGAGGCGTGGTGCAATTCG--AGTGACTAATCCAGGTTTGGAAACAGGCAG
PVM KY364848.1        TTCTGCGTGAAAGGCGTGGTGCAATTCG--GGTGACTAATCCTGGTTTGGAAACAGGCAG
PVM MT114148.1        TTCTGCGTGAAAGGCGTGGCGCAATTCG--GGTGACAAATCCTGGTTTGGAAACAGGTAG
                      ** *  *    ** *  *** ******   * ***  ***      ** ****  ** **

PVS MK442089.1        GCCCTCGCTTGAGCCGACCCCTGAAATGCGGAGGAATCCGGAGAATCCATACTCGCGGTT
PVM HM854296.1        GCCAAGGTTGCAGCTCGCYGAAAATATGCGCCCYGATCCCACTAATCCGTACAACAGGCC
PVM AJ437481.1        GCCAAGGTTGCAGCTAGCTGAAAATATGCGCCCAGATCCTACGAATCCGTACAACAGGCC
PVM JN835299.1        GCCAAGGTTGCTGCTAGCTGAAAATATGCGCCCAGATCCTACGAATCCGTACAACAGGCC
PVM AY311394.1        GCCGAGGTTGCAGCTAGCTGAAAATATGCGCCCAGATCCTACGAATCCGTACAACAGGCC
PVM AY311395.1        GCCAAGGTTGCAGCTAGCTGAAAATATGCGCCCAGATCCTACGAATCCGTACAACAGGCC
PVM MH558036.1        GCCAAGGTTGCAGCTAGCTGAGAATATGCGCCCTGATCCCACGAATCCGTACAACAGGCC
PVM MH558037.1        GCCAAGGTTGCAGCTAGCTGAGAATATGCGCCCTGATCCCACGAATCCGTACAACAGGCC
PVM D14449.2          GCCAAGGTTGCAGCTAGCTGAAAATATGCGCCCTGATCCCACGAATCCGTACAACAGGCC
PVM NC_001361.2       GCCAAGGTTGCAGCTAGCTGAAAATATGCGCCCTGATCCCACGAATCCGTACAACAGGCC
PVM JX678982.1        GCCAAGGTTGCAGCTAGCTGAAAATATGCGCCCAGATCCCACGAATCCGTACAACAGGCC
PVM EU604672.1        GCCAAGGTTGCAGCTAGCTGAAAACATGCGCCCTGATCCCACGAATCCGTACAACAGGCC
PVM MW582794.1        GCCAAGGTTGCAGCTAGCTGAAAACATGCGCCCTGATCCCACGAATCCGTACAACAGGCC
PVM MT114149.1        TCCACGACTCAAGCTAGCAGACGATATGCGCCCGGATCCGACTAATCCTTACAACAGGCC
PVM MG356508.1        GCCGAAACTGAGATTAGCAGAGGACATGCGTCCAGATCCAACGAATCCTTACAACAGACC
PVM MG356509.1        GCCGAAACTGAGATTAGCAGAGGACATGCGTCCAGATCCAACGAATCCTTACAACAGACC
PVM MF133527.1        GCCGAAACTGAGACTAGCAGAGGACATGCGCCCAGATCCAACGAATCCTTACAACAGACC
PVM MF133530.1        GCCGAAACTGAGACTAGCAGAGGACATGCGTCCAGATCCAACGAATCCTTACAACAGACC
PVM MW307264.1        GCCAAAGTTAAAGCTGGCGGAGGACATGCGTCCCGACCCGACTAATCCCTACAACAGGCC
PVM MF133528.1        GCCAAGATTGAATCTAGCTGAGAATATGCGTCCTGATCCGACAAACCCGTACAACAGGCC
PVM MF133529.1        GCCAAGATTGAATCTAGCTGAGGATATGCGTCCTGATCCGACAAACCCGTACAACAGGCC
PVM KY364848.1        GCCAAAATTGAAGCTAGCTGAGGATATGCGTCCTGACCCGACAAACCCGTACAACAGGCC
PVM MT114148.1        GCCAAAATTGAAGCTAGTTGAGGATATGCGTCCTGACCCGACAAACCCGTACAACAGGCC
                      ***   * *  ***   *     * *****     ****   ****** ***    **

PVS MK442089.1        TTCAATCGATGAGCTGTTCAAGATGGAAATCCGAT--CTGTGTCCAACAACATGGCGAAC
PVM HM854296.1        GTCCATAGAAGCTCTCAGCCGGAT--CAAGCCAATCGCGATCTCAAACAATATGGCTACA
PVM AJ437481.1        GTCCATAGAAGCCCTCAGCCGAAT--CAAGCCAATCGCGATCTCAAACAATATGGCTACG
PVM JN835299.1        GTCCATAGAAGCTCTCAGCCGAAT--CAAACCAATCGCGATCTCGAACAATATGGCTACG
PVM AY311394.1        GTCCATAGAAGCTCTCAGCCGGAT--CAAGCCAATCGCGATCTCGAACAATATGGCTACG
PVM AY311395.1        GTCCATAGAAGCTCTCAGCCGGAT--CAAGCCAATCGCGATCTCAAACAATATGGCTACG
PVM MH558036.1        GTCCATAGAAGCTCTCAGCCGGAT--CAAGCCGATCGCGATCTCAAACAATATGGCTACA
PVM MH558037.1        GTCCATAGAAGCTCTCAGCCGGAT--CAAGCCGATCGCGATCTCAAACAATATGGCTACA
PVM D14449.2          GTCCATAGAAGCTCTCAGCCGGAT--CAAGCCAATCGCGATCTCAAACAATATGGCCACA
PVM NC_001361.2       GTCCATAGAAGCTCTCAGCCGGAT--CAAGCCAATCGCGATCTCAAACAATATGGCCACA
PVM JX678982.1        GTCCATAGAAGCTCTCAGCCGGAT--CAAGCCAATCGCGATCTCGAACAATATGGCCACA
PVM EU604672.1        ATCCATAGAAGCTCTCAGCCGGAT--CAAGCCAATCGCGATCTCAAACAATATGGCCACA
PVM MW582794.1        ATCCATAGAAGCTCTCAGCCGGAT--CAAGCCAATCGCGATCTCAAACAATATGGCCACA
PVM MT114149.1        ATCGCTTGAGGCGCTGAGCAGAAT--TAAACCCATAGCTGTATCGAACAACATGGCCACA
PVM MG356508.1        TTCTATAGAGGCTTTAAGCAGGAT--CAAGCCGATAGCTGTGTCGAACAACATGGCCACA
PVM MG356509.1        TTCTATAGAGGCTTTAAGCAGGAT--CAAGCCGATAGCTGTGTCGAACAACATGGCCACA
PVM MF133527.1        TTCTATAGAGGCTTTAAGCAGAAT--CAAGCCGATAGCCGTGTCGAACAACATGGCCACA
PVM MF133530.1        TTCTATAGAGGCTTTAAGCAGAAT--CAAGCCAATAGCCGTGTCGAACAACATGGCTACA
PVM MW307264.1        TTCCATTGAAGCACTCAGCAGAAT--CAAGCCGATTGCCATATCCAATAACATGGCTACA
PVM MF133528.1        TTCACTAGAAGCGCTAAGCAGGAT--AAAACCGATAGCTATATCGAACAACATGGCCACA
PVM MF133529.1        TTCACTAGAAGCGCTAAGCAGGAT--AAAACCGATAGCTATATCGAACAATATGGCCACG
PVM KY364848.1        TTCAATAGAAGCGCTAAGCAGGAT--CAAACCGATAGCCATATCGAACAATATGGCCACA
PVM MT114148.1        TTCAATAGAGGCGCTAAGCAGGAT--CAAACCGATAGCTATATCGAACAATATGGCCACA
                       ** ** ** *  **   *  ***   ** ** **  *  * **  ***** ***** *
```

```
PVS MK442089.1       ACTGAGCAAATGGCACAAATCACTGCTGATATCGCTGGGCTTGGGGTCCCCACTGAACAC
PVM HM854296.1       TCTGAGGATATGATGCGCATATATGTGAACCTGGAGGGGCTAGGTGTGCCGACTGAGCAC
PVM AJ437481.1       TCTGAGGATATGATGCGCATATATGTGAACCTGGAGGGGCTAGGTGTGCCGACTGAGCAC
PVM JN835299.1       TCTGAGGATATGATGCGCATATATGTGAACCTGGAGGGGCTAGGTGTGCCGACTGAGCAC
PVM AY311394.1       TCTGAGGATATGATGCGCATATATGTGAACCTGGAGGGGCTAGGTGTGCCGACTGAGCAC
PVM AY311395.1       TCTGAGGATATGATGCGCATATATGTGAACCTGGAAGGGCTAGGTGTGCCGACTGAGCAC
PVM MH558036.1       TCTGAGGACATGATGCGCATATATGTGAACCTGGAGGGGCTAGGTGTGCCGACTGAGCAC
PVM MH558037.1       TCTGAGGACATGATGCGCATATATGTGAACCTGGAGGGGCTAGGTGTACCGACTGAGCAC
PVM D14449.2         TCTGAGGATATGATGCGCATATATGTGAACCTGGAGGGGCTAGGGGTGCCGACTGAGCAC
PVM NC_001361.2      TCTGAGGATATGATGCGCATATATGTGAACCTGGAGGGGCTAGGGGTGCCGACTGAGCAC
PVM JX678982.1       TCTGAGGACATGATGCGCATATATGTGAACCTGGAGGGGCTAGGGGTGCCGACTGAGCAC
PVM EU604672.1       TCTGAGGATATGATGCGCATATATGTGAACCTGGAGGGGCTAGGGGTGCCGACTGAGCAC
PVM MW582794.1       TCTGAGGATATGATGCGCATATATGTGAACCTGGAGGGGCTAGGGGTGCCGACTGAGCAC
PVM MT114149.1       TCTGAGGACATGATGCGCATCTACGTCAATCTTGAAGGCTTGGGAGTGCCGACTGAGTAC
PVM MG356508.1       TCTGAGGACATGATGCGCATTTATGTAAACCTCGAAGGGTTGGGCGTACCTACAGAGCAT
PVM MG356509.1       TCTGAGGACATGATGCGCATTTATGTAAACCTCGAAGGGTTGGGCGTACCTACAGAGCAT
PVM MF133527.1       TCTGAGGACATGATGCGCATTTATGTGAACCTCGAAGGGTTGGGCGTACCTACAGAGCAC
PVM MF133530.1       TCTGAAGACATGATGCGCATTTATGTGAACCTCGAAGGGTTGGGCGTACCTACAGAGCAT
PVM MW307264.1       TCGGAGGACATGATGCGCATATATGTGAACCTTGAGGGGCTGGGCGTGCCGACAGAGCAT
PVM MF133528.1       TCTGAGGACATGATGCGCATATATGTAAACCTGGAAGGTCTGGGTGTGCCTACTGAACAT
PVM MF133529.1       TCTGAGGACATGATGCGCATATATGTAAACCTGGAAGGTCTGGGTGTGCCTACTGAACAT
PVM KY364848.1       TCTGAGGACATGATGCGCATATATGTAAATCTGGAAGGCCTGGGTGTGCCTACTGAACAC
PVM MT114148.1       TCTGAAGACATGATGCGCATATATGTAAACCTGGAAGGTCTGGGTGTGCCTACTGAACAC
                      ***** * ***   *  **   **   *  * *  ***** ** ** ** ***** ***

PVS MK442089.1       GTTGCAG-GGGTAATACTGAAAGTGGTGATCATGTGTGCAAGCGTGAGTAGCTCTGTTTA
PVM HM854296.1       G-TGCAGCAGGTAGTGATTCAGGCAGTGCTATTTTGCAAGGACGCAAGCAGCTCCGTATT
PVM AJ437481.1       G-TGCAGCAGGTAGTGATTCAGGCAGTGCTATTTTGCAAGGACGCAAGCAGCTCCGTATT
PVM JN835299.1       G-TGCAACAGGTAGTGATTCAGGCAGTGCTATTTTGCAAGGACGCAAGCAGCTCCGTATT
PVM AY311394.1       G-TGCAGCAGGTAGTGATCCAGGCAGTGCCATTTTGCAAGGACGCAAGCAGCTCCGTATT
PVM AY311395.1       G-TGCAGCAGGTAGTGATCCAGGCAGTGCTATTTTGCAAGGACGCAAGCAGCTCCGTATT
PVM MH558036.1       G-TGCAGCAGGTAGTGATTCAGGCAGTGCTATTTTGCAAGGATGCGAGCAGCTCCGTATT
PVM MH558037.1       G-TGCAGCAGGTAGTGATTCAGGCAGTGCTATTTTGCAAGGATGCGAGCAGCTCCGTATT
PVM D14449.2         G-TGCAGCAGGTAGTGATTCAGGCTGTGCTATTTTGCAAGGACGCAAGCAGCTCCGTATT
PVM NC_001361.2      G-TGCAGCAGGTAGTGATTCAGGCTGTGCTATTTTGCAAGGACGCAAGCAGCTCCGTATT
PVM JX678982.1       G-TGCAGCAGGTAGTGATTCAGGCTGTGCTATTTTGCAAGGATGCAAGCAGCTCCGTATT
PVM EU604672.1       G-TGCAGCAGGTAGTGATTCAGGCTGTGCTATTTTGCAAGGACGCAAGCAGCTCCGTATT
PVM MW582794.1       G-TGCAGCAGGTAGTGATTCAGGCTGTGCTATTTTGCAAGGACGCAAGCAGCTCCGTATT
PVM MT114149.1       G-TGCAGCAAGTGGTGATCCAAGCGGTGCTTTTCTGCAAAGATGCAAGTAGCTCGGTGTA
PVM MG356508.1       G-TGCAGCAAGTGGTTATTCAGGCCGTGTTATTCTGTAAGGATGCGAGCAGTTCTGTGTA
PVM MG356509.1       G-TGCAGCAAGTGGTTATTCAGGCCGTGTTATTCTGTAAGGATGCGAGCAGTTCTGTGTA
PVM MF133527.1       G-TGCAGCAAGTGGTGATTCAGGCCGTGTTATTCTGCAAGGATGCGAGCAGTTCTGTGTA
PVM MF133530.1       G-TGCAGCAAGTGGTGATTCAGGCCGTGTTATTCTGCAAGGATGCGAGCAGTTCTGTGTA
PVM MW307264.1       G-TGCAACAAGTTGTGATACAGGCCGTGCTGTTTTGCAAGGATGCTAGTAGCTCTGTGTA
PVM MF133528.1       G-TGCAGCAGGTGGTTATTCAAGCTGTGTTGTTCTGCAAAGATGCCAGCAGTTCTGTGTA
PVM MF133529.1       G-TGCAGCAGGTGGTTATTCAAGCTGTGTTGTTCTGCAAAGATGCTAGCAGCTCTGTGTA
PVM KY364848.1       G-TGCAGCAGGTGGTCATTCAAGCTGTGTTGTTCTGCAAAGATGCTAGCAGCTCTGTGTA
PVM MT114148.1       G-TGCAGCAAGTGGTCATACAAGCTGTGTTGTTCTGCAAAGATGCTAGCAGCTCTGTGTA
                     * *****  **** *  *  * *  *** *  * **       *  ** ***** ** *

PVS MK442089.1       TCTAGATCCAGCAGGGACTGTGGAGTTCCCAACAGGCGCAGTGCCCTTGGACTCAATCAT
PVM HM854296.1       CCTGGATCCGCGAGGTTCGTTTGAGTGGCCCCGAGGTGCTATAACTGCAGATGCCGTCTT
PVM AJ437481.1       CCTGGATCCGCGAGGCTCGTTCGAATGGCCCAGAGGTGCTATAACCGCAGATGCCGTCTT
PVM JN835299.1       CCTGGATCCGCGAGGTTCGTTCGAATGGCCCAGAGGTGCTATAACCGCAGATGCCGTCTT
PVM AY311394.1       CCTGGATCCGCGAGGCTCGTTCGAATGGCCCAGAGGTGCTATAACTGCAGATGCCGTCTT
PVM AY311395.1       CCTGGATCCGCGAGGCTCGTTTGAATGGCCCAGAGGTGCTATAACTGCAGACGCCGTCTT
PVM MH558036.1       CCTGGACCCGCGAGGCTCATTCGAGTGGCCCAGAGGCGCTATAACTGCAGATGCCGTCTT
PVM MH558037.1       CCTGGACCCGCGAGGCTCATTCGAGTGGCCCAGAGGTGCTATAACTGCAGATGCCGTCTT
PVM D14449.2         CCTGGATCCGCGAGGCTCGTTCGAGTGGCCAAGAGGTGCTATAACTGCAGATGCCGTCTT
PVM NC_001361.2      CCTGGATCCGCGAGGCTCGTTCGAGTGGCCAAGAGGTGCTATAACTGCAGATGCCGTCTT
PVM JX678982.1       CCTGGATCCGCGAGGCTCGTTCGAGTGGCCAAGAGGTGCTATAACTGCAGATGCCGTCTT
PVM EU604672.1       CCTGGATCCGCGAGGCTCGTTCGAGTGGCCAAGAGGTGCTATAACTGCGGATGCCGTCTT
PVM MW582794.1       CCTAGATCCGCGAGGCTCGTTCGAGTGGCCAAGAGGTGCTATAACTGCGGATGCCGTCTT
PVM MT114149.1       TCTCGATCCACGCGGCTCCTTCGAATGGCCAGGGGGTGCGATCACAGCAGATGCGGTGTT
PVM MG356508.1       CTTGGATCCGCGCGGGTCCTTTGAGTGGCCCGGGGGCGCGATCACTGCGGATGCTGTCCT
PVM MG356509.1       CTTGGATCCGCGCGGGTCCTTTGAGTGGCCCGGGGGCGCGATCACTGCGGATGCTGTCCT
PVM MF133527.1       CTTGGATCCGCGCGGGTCCTTTGAGTGGCCCGGGGGCGCGATCACAGCGGATGCTGTCCT
PVM MF133530.1       CTTGGATCCGCGCGGGTCCTTTGAGTGGCCCGGGGGTGCGATCACTGCGGATGCTGTCCT
PVM MW307264.1       TCTAGATCCGAGGGGATCATTCGAGTGGCCTAGGGGTGCCATCACGGCGGACGCTGTGCT
PVM MF133528.1       CTTGGACCCACGTGGGTCGTTCGAATGGCCTAGAGGTGCCATCACAGCGGATGCTGTTCT
PVM MF133529.1       CCTGGACCCACGCGGGTCGTTCGAATGGCCTAGAGGTGCCATCACAGCGGATGCTGTTCT
PVM KY364848.1       CTTGGACCCGCGTGGTTCGTTCGAGTGGCCTAGAGGTGCCATCACAGCGGATGCAGTTCT
PVM MT114148.1       CCTGGACCCGCGCGGGTCGTTTGAGTGGCCTAGGGGTGCCATCACAGCGGATGCTGTTCT
                      ** *****   ***  *  * ****  ** * *** **  *  *    **  *  ** *
```

```
PVS MK442089.1        TGCAATTATGAAGAATCGCGCGGGA---TTGAGAAAAGTGTGCAGGCTGTATGCTCCAGT
PVM HM854296.1        GGCTGTGTTGAAGAAGGATGCAGAAACACTACGAAGGGTGTGTAGGCTGTATGCCCCGGT
PVM AJ437481.1        GGCTGTGCTGAAGAAGGATGCGGAAACACTGCGAAGGGTGTGTAGGCTGTATGCCCCGGT
PVM JN835299.1        GGCTGTGCTGAAGAAGGATGCGGAAACACTGCGAAGGGTGTGTAGGCTGTATGCCCCGGT
PVM AY311394.1        GGCTGTGCTGAAGAAGGATGCGGAAACACTGCGAAGGGTGTGTAGGCTGTATGCCCCGGT
PVM AY311395.1        GGCTGTGCTGAAGAAGGATGCGGAAACACTGCGAAGGGTGTGTAGGCTGTATGCCCCGGT
PVM MH558036.1        GGCCGTGCTGAAGAAGGATGCAGAGACACTACGAAGGGTGTGTAGGCTGTATGCCCCGGT
PVM MH558037.1        GGCCGTGCTGAAGAAGGATGCAGAGACACTACGAAGGGTGTGTAGGCTGTATGCCCCGGT
PVM D14449.2          GGCTGTGCTGAAGAAGGATGCAGAAACACTGCGAAGGGTGTGTAGGCTGTATGCCCCGGT
PVM NC_001361.2       GGCTGTGCTGAAGAAGGATGCAGAAACACTGCGAAGGGTGTGTAGGCTGTATGCCCCGGT
PVM JX678982.1        GGCTGTGCTGAAGAAGGATGCAGAAACACTACGAAGGGTGTGTAGGCTGTATGCCCCGGT
PVM EU604672.1        GGCTGTGCTGAAGAAGGATGCTGAAACACTACGAAGGGTGTGTAGGCTGTATGCCCCGGT
PVM MW582794.1        GGCTGTGCTGAAGAAGGATGCTGAAACACTACGAAGGGTGTGTAGGCTGTATGCCCCGGT
PVM MT114149.1        GGCGGTGATGAAGAAAGACGCAGAGACCCTGCGCAGAGTCTGTAGGCTGTACGCGCCCGT
PVM MG356508.1        CGCAGTAATGAAGAAGGATGCGGAAACTCTACGCAGGGTTTGTAGGCTGTATGCACCTGT
PVM MG356509.1        CGCAGTAATGAAGAAGGATGCGGAAACTCTACGCAGGGTTTGTAGGCTGTATGCACCTGT
PVM MF133527.1        CGCAGTAATGAAGAAGGATGCGGAAACTCTACGCAGGGTCTGCAGGCTGTATGCACCAGT
PVM MF133530.1        CGCAGTAATGAAGAAGGATGCGGAAACTCTACGCAGGGTTTGTAGGCTGTATGCACCTGT
PVM MW307264.1        CGCTGTGATGAAGAAGGATGCTGAAACTTTGCGCAGAGTCTGCAGGTTGTACGCTCCTGT
PVM MF133528.1        TGCCGTGATGAAGAAGGATGCGGAGACTCTAAGGAGGGTTTGTAGGCTATACGCACCTGT
PVM MF133529.1        TGCCGTGATGAAGAAGGATGCGGAGACTCTAAGGAGGGTTTGTAGGCTATACGCCCCTGT
PVM KY364848.1        TGCCGTGATGAAGAAGGACGCGGAGACTCTAAGGAGGGTTTGTAGGCTATACGCACCTGT
PVM MT114148.1        TGCCGTGATGAAGAAGGACGCAGAGACTCTAAGGAGGGTTTGTAGGTTATATGCACCTGT
                       **  *  *******    ** * *    *  ***  ***** *********** ** **

PVS MK442089.1        CGTGTGGAATTACATGCTAGTCCAGAATAGGCCACCTTCGGATTGGCAGGCCATGGGATT
PVM HM854296.1        GACATGGAATCAYATGCTGACGCATAACGCGCCTCCAGCCGACTGGGCAGCCATGGGGTT
PVM AJ437481.1        AACATGGAATCACATGCTGACGCACAACGCGCCTCCAGCCGACTGGGCTGCCATGGGGTT
PVM JN835299.1        AACATGGAATCACATGCTGACGCACAATGCGCCCCCAGCTGACTGGGCTGCCATGGGGTT
PVM AY311394.1        GACATGGAATCATATGCTGACGCACAACGCGCCTCCAGCCGACTGGGCTGCCATGGGGTT
PVM AY311395.1        GACATGGAATCATATGCTGACGCACAACGCGCCTCCAGCCGACTGGGCTGCCATGGGGTT
PVM MH558036.1        GACATGGAATCATATGCTGACGCACAACGCGCCTCCAGCCGACTGGGCTGCCATGGGGTT
PVM MH558037.1        GACATGGAATCATATGCTGACGCACAACGCGCCTCCAGCCGACTGGGCTGCCATGGGGTT
PVM D14449.2          GACATGGAATCATATGCTGACGCACAACGCGCCTCCGGCCGATTGGGCTGCCATGGGGTT
PVM NC_001361.2       GACATGGAATCATATGCTGACGCACAACGCGCCTCCGGCCGATTGGGCTGCCATGGGGTT
PVM JX678982.1        GACATGGAATCATATGCTGACGCATAACGCGCCTCCGGCCGACTGGGCTGCCATGGGGTT
PVM EU604672.1        GACATGGAATCATATGCTGACGCACAACGCGCCTCCAGCCGACTGGGCTGCCATGGGGTT
PVM MW582794.1        GACATGGAATCATATGCTGACGCACAACGCGCCTCCAGCCGACTGGGCTGCCATGGGGTT
PVM MT114149.1        CACGTGGAATCATATGCTGACGCACAATTCTCCACCAGCAGACTGGGCGGCTATGGGTTT
PVM MG356508.1        CACCTGGAATCACATGTTGACTCACAACTCACCCCCAGCAGATTGGGCTGCCATGGGCTT
PVM MG356509.1        CACCTGGAATCACATGTTGACTCACAACTCACCCCCAGCAGATTGGGCTGCCATGGGCTT
PVM MF133527.1        CACCTGGAATCACATGTTGACTCACAACTCACCCCCAGCAGATTGGGCTGCCATGGGCTT
PVM MF133530.1        CACCTGGAATCACATGTTGACTCACAACTCACCCCCAGCAGATTGGGCTGCCATGGGCTT
PVM MW307264.1        GACTTGGAATCACATGCTGACCCACAACTCGCCTCCCGCTGATTGGGCCGCTATGGGCTT
PVM MF133528.1        GACCTGGAATCATATGCTGACTCACAACTCACCTCCGGCTGATTGGGCAGCCATGGGTTT
PVM MF133529.1        GACCTGGAATCATATGTTGACTCACAACTCGCCTCCGGCTGATTGGGCAGCCATGGGATT
PVM KY364848.1        GACCTGGAATCATATGTTGACTCACAACTCACCTCCGGCTGATTGGGCAGCCATGGGTTT
PVM MT114148.1        GACCTGGAATCACATGTTGACTCACAACTCTCCCCCGGCTGACTGGGCAGCCATGGGTTT
                          ****** * *****    ** **   *** **  * ** ***   ******** **

PVS MK442089.1        CCAGTGGAACGCACGCTTCGCCGCATTTGACACATTCGATTATGTGACTAATGGGGCTGC
PVM HM854296.1        TCAGTACGAGGATCGCTTCGCTGCTTTCGACTGCTTTGATTACGTTGAGAACACAGCTGC
PVM AJ437481.1        TCAGTACGAGGATCGCTTCGCTGCCTTCGACTGCTTTGATTACGTTGAGAATACTGCAGC
PVM JN835299.1        TCAGTACGAGGACCGCTTCGCTGCCTTCGACTGCTTTGATTACGTTGAGAATACTGCAGC
PVM AY311394.1        TCAGTACGAGGATCGCTTCGCTGCCTTCGACTGCTTTGATTACGTTGAGAATACTGCAGC
PVM AY311395.1        TCAGTACGAGGATCGCTTCGCTGCCTTCGACTGCTTTGATTACGTTGAGAATACTGCAGC
PVM MH558036.1        TCAGTACGAAGATCGCTTCGCTGCTTTCGACTGTTTCGATTACGTTGAGAATACTGCTGC
PVM MH558037.1        TCAGTACGAAGATCGCTTCGCTGCTTTCGACTGTTTTGATTACGTTGAGAATACTGCTGC
PVM D14449.2          TCAGTATGAGGATCGCTTCGCTGCTTTCGACTGCTTTGATTACGTTGAGAATACTGCTGC
PVM NC_001361.2       TCAGTATGAGGATCGCTTCGCTGCTTTCGACTGCTTTGATTACGTTGAGAATACTGCTGC
PVM JX678982.1        TCAGTATGAGGATCGCTTCGCTGCCTTCGACTGCTTTGATTACGTTGAGAATACTGCTGC
PVM EU604672.1        TCAGTATGAGGATCGCTTCGCTGCTTTCGACTGCTTTGATTACGTTGAGAATACTGCTGC
PVM MW582794.1        TCAGTATGAGGATCGCTTCGCTGCTTTCGACTGCTTTGATTACGTTGAGAATACTGCTGC
PVM MT114149.1        CCAGTATGAAGACAGATTTGCGGCCTTTGATTGCTTTGACTACGTGGAGAATACAGCTGC
PVM MG356508.1        CCAGTACGAAGACCGATTTGCGGCTTTTGACTGCTTCGACTATGTGGAGAATACGGCAGC
PVM MG356509.1        CCAGTACGAAGACCGATTTGCGGCTTTTGACTGCTTCGACTATGTGGAGAATACGGCAGC
PVM MF133527.1        CCAGTACGAAGACCGATTTGCTGCTTTTGACTGCTTCGACTATGTGGAGAATACGGCGGC
PVM MF133530.1        CCAGTACGAAGACCGATTTGCTGCTTTTGACTGCTTCGACTATGTGGAGAACACGGCTGC
PVM MW307264.1        TCAATATGAGGATCGGTTCGCCGCTTTCGACTGCTTTGATTACGTGGAGAACACTGCTGC
PVM MF133528.1        CCAATACGAGGACCGCTTTGCTGCGTTCGACTGCTTCGACTACGTCGAGAACACAGCGGC
PVM MF133529.1        CCAATACGAGGACCGCTTTGCTGCATTCGACTGCTTCGACTACGTCGAGAACACAGCGGC
PVM KY364848.1        CCAATACGAGGAACGCTTTGCTGCATTCGACTGCTTCGACTACGTCGAGAACACAGCGGC
PVM MT114148.1        CCAATACGAGGACCGTTTTGCTGCATTCGACTGCTTCGACTACGTCGAGAACACAGCGGC
                       ****   * *  ******** ** ** ***   ** ***** **    ***   *****
```

```
PVS MK442089.1      AATTCAGCCCGTAGAGGGGCTCATACGCAGGCCCACACCTGAGGAAACAATAGCTCACAA
PVM HM854296.1      AGTCCAACCCCTAGAGGGATTGATCAGGCGACCTACCCCAAGGGAAAAGATAGCTCACAA
PVM AJ437481.1      AGTTCAGCCCCTAGAGGGATTGATCAGGCGACCTACCCCAAGGGAGAAGATAGCTCACAA
PVM JN835299.1      AGTTCAGCCCCTAGAGGGATTGATCAGGCGACCTACCCCAAGGGAGAAGATAGCTCACAA
PVM AY311394.1      AGTTCAGCCCCTAGAGGGATTGATCAGGCGACCTACCCCAAGGGAGAAGATAGCTCACAA
PVM AY311395.1      AGTTCAGCCCCTAGAGGGATTGATCAGGCGACCTACCCCAAGGGAGAAGATAGCTCACAA
PVM MH558036.1      AGTTCAACCCCTAGAGGGATTGATCAGGCGACCTACTCCAAGGGAAAAGATAGCTCACAA
PVM MH558037.1      AGTTCAACCCCTAGAGGGATTGATCAGGCGGCCTACTCCAAGGGAAAAGATAGCTCACAA
PVM D14449.2        AGTCCAACCCCTAGAGGGATTGATCAGGCGACCTACCCCAAGGGAAAAGGTAGCTCACAA
PVM NC_001361.2     AGTCCAACCCCTAGAGGGATTGATCAGGCGACCTACCCCAAGGGAAAAGGTAGCTCACAA
PVM JX678982.1      AGTCCAACCCCTAGAGGGATTGATCAGGCGACCTACCCCGAGGGAAAAGGTAGCTCATAA
PVM EU604672.1      AGTCCAACCCCTAGAGGGATTGATCAGGCGACCTACCCCAAGGGAAAAGATAGCTCACAA
PVM MW582794.1      AGTCCAACCCCTAGAGGGATTGATCAGGCGACCTACCCCAAGGGAAAAGATAGCTCACAA
PVM MT114149.1      AGTTCAGCCGCTGGAAGGGCTAATACGCCGACCCACTCCAAGAGAGAAGGTTGCGCACAA
PVM MG356508.1      GGTTCAACCTTTGGAGGGCTTGATAAGGCGACCCACCCCAAGAGAGAAGGTGGCTCACAA
PVM MG356509.1      GGTTCAACCTTTGGAGGGCTTGATAAGGCGACCCACCCCAAGAGAGAAGGTGGCTCACAA
PVM MF133527.1      GGTTCAACCTTTGGAGGGCTTGATAAGGCGACCCACCCCAAGAGAGAAGGTGGCTCATAA
PVM MF133530.1      GGTTCAACCTTTGGAGGGCTTGATAAGGCGACCCACCCCAAGAGAGAAGGTGGCTCACAA
PVM MW307264.1      AGTTCAACCACTGGAGGGCCTGATAAGGCGACCAACCCCGAGAGAGAAGGTTGCTCACAA
PVM MF133528.1      TGTTCAACCATTGGAAGGATTGATAAGGAGGCCCACTCCGAGAGAGAAGGTTGCTCACAA
PVM MF133529.1      CGTTCAACCACTGGAAGGATTAATAAGGAGGCCGACTCCGAGAGAGAAGGTCGCTCACAA
PVM KY364848.1      CGTGCAGCCACTGGAAGGATTGATAAGGAGGCCCACTCCGAGAGAGAAGGTTGCTCACAA
PVM MT114148.1      CGTCCAGCCACTGGAAGGATTGATAAGGAGGCCCACTCCGAGAGAGAAGGTTGCTCACAA
                    * **** *** *******  * **  *  * ** ** **   *** *   **********

PVS MK442089.1      TGCCCACAAGAGTATGGCGATTGACAAGTCGAACAGAAATGAGCGATTGGCCAACACGAA
PVM HM854296.1      TACGCACAAAGACGTCGCACTGCGTGGAGCAAATCGCAATCAGGTGTTCAGCTCTCTCAA
PVM AJ437481.1      TACGCACAAAGACATCGCACTGCGTGGGGCAAATCGCAATCAAGCGTTCAGCTCTCTCAA
PVM JN835299.1      TACGCACAAAGACATCGCGCTGCGTGGAGCAAATCGCAATCAAGCGTTCAGCTCTCTCAA
PVM AY311394.1      TACGCACAAAGACATCGCGCTGCGTGGAGCAAATCGCAATCAAGCTTTCAGCTCTCTCAA
PVM AY311395.1      TACGCACAAAGACATCGCGCTGCGTGGAGCAAATCGCAATCAAGCGTTCAGCTCTCTCAA
PVM MH558036.1      CACGCACAAAGACATTGCACTGCGTGGAGCAAATCGCAATCAGGTGTTCAGCTCTCTCAA
PVM MH558037.1      CACGCACAAAGACATTGCACTGCGTGGAGCAAATCGCAATCAGGTGTTCAGCTCTCTCAA
PVM D14449.2        TACGCACAAAGACATCGCAGTGCGTGGAGCAAATCGCAATCAGGTGTTCAGCTCTCTCAA
PVM NC_001361.2     TACGCACAAAGACATCGCAGTGCGTGGAGCAAATCGCAATCAGGTGTTCAGCTCTCTCAA
PVM JX678982.1      TACGCACAAAGACATCGCACTGCGTGGAGCAAATCGCAATCAGGTGTTCAGCTCTCTCAA
PVM EU604672.1      TACGCACAAAGACATCGCACTGCGTGGAGCAAATCGCAATCAGGTGTTCAGCTCTCTCAA
PVM MW582794.1      TACGCACAAAGACATCGCACTGCGTGGAGCAAATCGCAATCAGGTGTTCAGCTCTCTCAA
PVM MT114149.1      TACCCATAAGGATATAGCTCTGAGAGGGGCCAATCGCAACCAGGTGTTCAGTTCGCTCAA
PVM MG356508.1      CACCCACAAGGACATAGCTTTACGCGGTGCTAACCGCAATCAGGTGTTCAGCTCTCTAAA
PVM MG356509.1      CACCCACAAGGACATAGCTTTACGCGGTGCTAACCGCAATCAGGTGTTCAGCTCTCTAAA
PVM MF133527.1      CACTCACAAGGACATAGCTTTACGCGGTGCTAACCGCAACCAGGTGTTCAGCTCTCTAAA
PVM MF133530.1      CACTCACAAGGACATAGCTTTACGCGGTGCTAACCGCAATCAGGTGTTCAGCTCTCTAAA
PVM MW307264.1      CACCCATAAGGACATAGCTTTACGCGGTGCAAATCGCAATCAGGTGTTTAGCTCTCTTAA
PVM MF133528.1      TGCTCACAAGGATATAGCTTTGCGCGGTGCTAACCGCAATCAGGTGTTCAGCTCTCTCAG
PVM MF133529.1      TGCTCATAAGGATATAGCTCTGCGCGGTGCTAACCGCAATCAGGTGTTCAGCTCTCTCAG
PVM KY364848.1      TACTCACAAGGATATAGCCTTGCGCAGTGCTAACCGCAATCAGGTGTTCAGCTCTCTTAG
PVM MT114148.1      TACCCACAAGGATATGGCTTTGCGCGGTGCTAACCGCAATCAGGTGTTCAGCTCTCTTAG
                    * * *****    ** **  *        * **  * *** **   **   *      **

PVS MK442089.1      TGTTGAGTACACTGGAGGGATGCTTGGCGCCGAGATTGTGCGCAATCACCGTAATGCGAT
PVM HM854296.1      TGCCGAGGTCACTGGTGGCATGAATGGTCCGGAGCTCACTAGGGATTATGGAAAGTCGAA
PVM AJ437481.1      TGCCGAGGTCACTGGTGGCATGAATGGTCCGGAGCTCACTAGGGATTATGGAAAGTCGAA
PVM JN835299.1      TGCCGAGGTCACTGGTGGCATGAATGGCCCGGAGCTCACTAGGGATTATGGAAAGTCGAA
PVM AY311394.1      TGCCGAGGTCACTGGCGGCATGAATGGTCCAGAGCTCACTAGGGATTATGGAAAGTCGAA
PVM AY311395.1      TGCCGAGGTCACTGGTGGCATGAATGGTCCGGAGCTCACTAGGGATTATGGAAAGTCGAA
PVM MH558036.1      TGCCGAGGTCACTGGTGGTATGAATGGTCCGGAGCTCACTAGGGATTATGGAAAGTCGAA
PVM MH558037.1      TGCCGAGGTCACTGGTGGTATGAATGGTCCGGAGCTCACTAGGGATTATGGAAAGTCGAA
PVM D14449.2        TGCCGAGGTCACTGGTGGTATGAATGGTCCGGAGCTCACTAGAGATTATGTAAAGTCTAA
PVM NC_001361.2     TGCCGAGGTCACTGGTGGTATGAATGGTCCGGAGCTCACTAGAGATTATGTAAAGTCTAA
PVM JX678982.1      TGCCGAGGTCACTGGTGGTATGAATGGTCCGGAGCTCACTAGGGATTATGGAAAGTCGAA
PVM EU604672.1      TGCCGAGGTCACTGGTGGTATGAATGGTCCGGAACTCACTAGGGATTATGGAAAGTCGAA
PVM MW582794.1      TGCCGAGGTCACTGGTGGTATGAATGGTCCGGAACTCACTAGGGATTATGGAAAGTCGAA
PVM MT114149.1      CGCTGAGGTAACTGGAGGTATGAATGGTCCCGAGCTCACTAGGGATTTCAGCAAATCGAA
PVM MG356508.1      TGCCGAGGTGACGGGTGGCATGAATGGTCCAGAACTCACTAGGGACTACGGGAAATCGAA
PVM MG356509.1      TGCCGAGGTGACGGGTGGCATGAATGGTCCAGAACTCACTAGGGACTACGGGAAATCGAA
PVM MF133527.1      TGCCGAGGTGACGGGTGGCATGAATGGTCCAGAACTCACTAGGGACTACGGGAAATCGAA
PVM MF133530.1      TGCCGAGGTGACGGGTGGCATGAATGGTCCAGAACTCACTAGGGACTACGGAAAATCGAA
PVM MW307264.1      TGCTGAGGTGACCGGCGGCATGAATGGCCCTGAACTCACTAGAGATTACGGGAAATCGAA
PVM MF133528.1      TGCCGAGGTGACCGGTGGCATGAATGGTCCCGAACTCACTAGAGATTTCGGGAAATCGAA
PVM MF133529.1      TGCCGAGGTAACCGGCGGCATGAATGGTCCCGAACTCACTAGAGACTTCGGGAAATCGAA
PVM KY364848.1      CGCCGAGGTGACCGGTGGTATGAATGGCCCCGAACTCACTAGGGATTTCGGGAAATCGAA
PVM MT114148.1      TGCTGAGGTGACTGGTGGCATGAATGGCCCCGAACTCACTAGAGATTTCGGGAAATCGAA
                    **  ***  ****** ** ***  ***  * *** *     *  ** *  * **  ***
```

```
PVS MK442089.1        CAACCAATGAAG---GCAGACCGTTTAGCCATGTTATTA-------TTGTGTGTCCATCG
PVM HM854296.1        TAGGAAATGAAGCACGTAACCAGGGTGGCTTTACTTATAGCGAGAGCTATGTGTACTTCT
PVM AJ437481.1        TAGAAAATGAAGCACGTAACCAGGGTGGCTTTAATTATAGCGAGAGCTATGTGTACTTCT
PVM JN835299.1        TAGAAAATGAAGCACGTAACCAGGGTGGCTTTAATTATAGCAAGAGCTATGTGTACTTCT
PVM AY311394.1        TAGAAAATGAAGCACGTAACCAGGGTGGCTTTAATTATAGCGAGAGCTATGTATACTTCT
PVM AY311395.1        TAGAAAATGAAGCACGTAACCAGGGTGGCCTTAATAATAGCGAGAGCTATGTGTACTTCT
PVM MH558036.1        TAGAAAATGAAGCACGTAACCAGGGTGGCTTTACTTATAGCGAGAGCTATGTGTATTTCT
PVM MH558037.1        TAGAAAATGAAGCACGTAACCAGGGTGGCTTTACTTATAGCGAGAGCTATGTGCATTTCT
PVM D14449.2          TAGAAAATGAAGGACGTAACCAAGGTGGCTTTACTTATAGCGAGAGCTATGTGTGCCTCT
PVM NC_001361.2       TAGAAAATGAAGGACGTAACCAAGGTGGCTTTACTTATAGCGAGAGCTATGTGTGCCTCT
PVM JX678982.1        TAGAAAATGAAGCACGTAACCAAGGTGGCTTTACTTATAGCGAGAGCTATGTGTACTTCT
PVM EU604672.1        TAGAAAATGAAGCACGTAACCAAGGTGGCTTTACTTATAGCGAGAGCTATGTGTACTTCT
PVM MW582794.1        TAGAAAATGAAGCACGTAACCAAGGTGGCTTTACTTATAGCGAGAGCTATGTGTACTTCT
PVM MT114149.1        TAATAAATGAAGTGTGTGACCCAGACTGCGTTGCTTGTAGCTAGGGCTTTGTTTTTGCAT
PVM MG356508.1        CAATAAATGAAGTGGGTAACTCGGGCAGCTTTACTTGTAGCAAGGGCAATGTACTTACAT
PVM MG356509.1        CAATAAATGAAGTGGGTAACTCGGGCAGCTTTACTTGTAGCAAGGGCAATGTACTTACAT
PVM MF133527.1        CAATAAATGAAGTGGGTAACTCGTGCTGCTTTACTTGTAGCAAGGGCAATGTACTTACGT
PVM MF133530.1        CAATAAATGAAGTGGGTAACTCGGGCTGCTTTACTTGTAGCAAGGGCAATGTACTTACAT
PVM MW307264.1        CAATAAATGAAGTGTGTGTCTAGAACGTCTTTGTTAGTAGCTAGGGCAATGTATTTACAT
PVM MF133528.1        TAACAAATGAAGTGTGTGACTCAAACTGCTTTACTTGTAGCTAGGGCTATGTATTTACAT
PVM MF133529.1        TAACAAATGAAGTGTGTGACTCAAACTGCTTTACTTGTAGCTAGGGCTATATATTTACAT
PVM KY364848.1        TAACAAATGAAGTGTGTGACTCGAACTGCTTTACTTGTAGCTAGGGCTATGTATTTACAT
PVM MT114148.1        TAACAAATGAAGTGTGTGACTCGAACCGCTTTACTTGTAGCTAGGGCTATGTATTTACAT
                       *   *******   *            *  *  *  **          * *
```

PVS

25K ORF

```
MF418029.1      --------GTGTATGAGGAGGTTTGACAGCTTAGGTAATCAGCTTAGTAGTATTGA--AT
KR152654.1      --------GTGTATGAGGATGTTTGATAGCTTAGGTAATCAGCTTAGTAGTATTGA--AT
MF418035.1      --------GTGTATGAGGATGTTTGATAGCTTAGGTAATCAGCTTAGTAGTATTGA--AT
MF418032.1      --------GTTTATGAGGAGGTTTGATAGCTTAGGTAATCAGCTTGGTAGTATTGA--AT
MF496659.1      --------GTGTATGAGGAGGTTTGATAGCTTAGGTAATCAGCTTAGTAGTATTGA--AT
MF418028.1      --------GTGTATGAGGAGGTTTGACAGCTTAGGTAATCAGCTTAGTAGTATTGA--AT
MH282867.1      --------GTGTATGAGGAGGTTTGACAGCTTAGGTAATCAGCTTAGTAGTATTGA--AT
MK563994.1      --------GTGTATGAGGAGGTTTGACAGCTTAGGTAATCAGCTTAGTAGTATTGA--AT
JQ647830.1      --------GTGTATGAGGAGGTTTGACAGCTTAGGTAATCAGCTTAGTAGTATTGA--AT
MN095414.1      --------GTGTATGAGGAGGTTTGACAGCTTAGGTAATCAGCTTAGTAGTATTGA--AT
KU058657.1      --------GTGTATGAGGAGGTTTGACAGCTTAGGTAATCAGCTTAGTAGTATTGA--AT
KU896946.1      --------GTGTATGAGGAGGTTTGACAGCTTAGGTAATCAGCTTAGTAGTATTGA--AT
MF033144.1      --------GTGTATGAGGAGGTTTGACAGCTTAGGTAATCAGCTTAGTAGTATTGA--AT
MF418030.1      --------GTGTATGAGGATGTATGATAGCTTAGGTAATCAGCTTAGTAGTATTGA--AT
MF418031.1      --------GTGTATGAGGATGTTTGATAGCTTAGGTAATCAGCTTAGTAGTATTGA--AT
KP089978.1      --------GTGTATGAGGATGTTTGATAGCTTAGGTAATCAGCTTAGTAGTATTGA--AT
KU896945.1      --------GTGTATGAGGATGTTTGATAGCTTAGGTAATCAGCTTAGTAGTATTGA--AT
LC375228.1      --------GTGTATGAGGATGTTTGATAGCTTAGGTAATCAGCTTAGTAGTATTGA--AT
KC430335.1      --------GTGTATGAGGATATTTGATAGCTTAGGTAATCAGCTTAGTAGTATTGA--AT
MF418033.1      --------GTGTATGAGGATGTTTGATAGCTTAGGTAATCAGCTTAGTAGTATTGA--AT
MW331321.1      --------GTGTATGAGGATGTTTGATAGCTTAGGTAATCAGCTTAGTAGTATTGA--AT
LC375227.1      --------GTGTATGAGGATGTTTGATAGCTTAGGTAATCAGCTTAGTAGTATTGA--AT
MF375506.1      --------GTGTATGAGGATGTTTGATAGCTTAGGTAATCAGCTTAGTAGTATTGA--AT
MK442089.1      ATGTTTGAGTGTATGAGGATGTTTGATAGCTTAGGTAATCAGCTTAGTAGTATTGA--AT
AJ863510.1      --------GTGTATGAGGATGTTTGATAGCTTAGGTAATCAGCTTAGTAGTATTGA--AT
D00461.1        --------GTGTATGAGGAGGTTTGATAGCTTAGGTAATCAGCTTAGTAGTATTGAATAT
                        ** ******** ************************************  **

MF418029.1      ATATGGATGTGTTTTTGCAAGTTTTGAATAAATATAAGTTTGAGCGTGTTAGTAGTACGT
KR152654.1      ATATGGATGTGTTTTTGCAAATTCTGAATAAATATAAGTTTGAGCGTGTTAGTAGTACTT
MF418035.1      ATATGGATGTGTTTTTGCAAATTCTGAATAAATATAAGTTTGAGCGTGTTAGTAGTACTT
MF418032.1      ATATGGATGTGTTTTTGCAAGTTTTGAATAAGTACAAGTTTGAGCGTGTTAGCAGTACCT
MF496659.1      ATATGGATGTGTTTTTGCAAGTATTGAATAAGTATAAGTTTGAGCGTGTTAGTAGTACTT
MF418028.1      ATATGGATGTGTTTTTGCAAGTTTTGAATAAATATAAGTTTGAGCGTGTTAGTAGTGCTT
MH282867.1      ATATGGATGTGTTTTTGCAAGTTTTGAATAAATATAAGTTTGAGCGTGTTAGCAGTGCTT
MK563994.1      ATATGGATGTGTTTTTGCAAGTTTTGAATAAATATAAGTTTGAGCGTGTTAGTAGTGCTT
JQ647830.1      ATATGGATGTGTTTTTGCAAGTTTTGAATAAATATAAGTTTGAGCGTGTTAGTAGTGCTT
MN095414.1      ATATGGATGTGTTTTTGCAAGTTTTGAATAAATACAAGTTTGAGCGTGTTAGTAGTGCTT
KU058657.1      AAATGGATGTGTTTTTGCAAGTTTTGAATAAATACAAGTTTGAGCGTGTTAGTAGTGCTT
KU896946.1      ATATGGATGTGTTTTTGCAAGTTTTGAATAAATACAAGTTTGAGCGTGTTAGTAGTGCTT
MF033144.1      ATATGGATGTGTTTTTGCAAGTTTTGAATAAATACAAGTTTGAGCGTGTTAGTAGTGCTT
MF418030.1      ATATGGATGTGTTTTTGCAAATTTTGAATAAATATAAGTTTGAGCGTGTTAGTAGTACTT
MF418031.1      ATATGGATGTGTTTTTGCAAATTTTGAATAAATATAAGTTTGAGCGTGTTAGTAGTACTT
KP089978.1      ATATGGATGTGTTTTTGCAAATTTTGAATAAATATAAGTTTGAGCGTGTTAGTAGTACTT
KU896945.1      ATATGGATGTGTTTTTGCAAATTCTGAATAAATATAAGTTTGAGCGTGTTAGTAGTACTT
LC375228.1      ATATGGATGTGTTTTTGCAAGTTTTGAATAAATATAAGTTTGAGCGTGTTAGTAGTACTT
KC430335.1      ATATGGATGTGTTTTTGCAAATTTTGAATAAATATAAATTTGAGCGTGTTAGTAGTACTT
MF418033.1      ATATGGATGTGTTTTTGCAAATTTTGAATAAATATAAGTTTGAGCGTGTTAGTAGTACTT
MW331321.1      ATATGGATGTGTTTTTGCAAATTTTGAATAAATATAAGTTTGAGCGTGTTAGTAGTACTT
LC375227.1      ATATGGATGTGTTTTTGCAAGTTTTGAATAAATATAAGTTTGAGCGTGTTAGTAGTACTT
MF375506.1      ATATGGATGTGTTTTTGCAAATTTTGAATAAATATAAGTTTGAGCGTGTTAGTAGTACTT
MK442089.1      ATATGGATGTGTTTTTGCAAATTTTGAATAAATATAAGTTTGAGCGTGTTAGTAGCACTT
AJ863510.1      ATATGGATGTGTTTTTGCAAATTTTGAATAAATATAAGTTTGAGCGTGTTAGTAGTACTT
D00461.1        ATATGGATGTGTTTTTGCAAGTTTTGAATAAATATAAGTTTGAGCGTGTTAGTAGTACTC
                ******************** ********************************** ****

MF418029.1      TAAATAAACCAATAGTTGTTCATAGTGTACCCGGTGCTGGTAAGAGTTCAGCAATTAGGG
KR152654.1      TAAATAAACCAATAGTTGTTCATAGTGTACCTGGAGCTGGTAAGAGTTCCGCCATTAGGG
MF418035.1      TAAATAAACCAATAGTTGTTCATAGTGTACCTGGAGCTGGTAAGAGTTCTGCTATTAGGG
MF418032.1      TAAATAAACCAATAGTTGTGCATAGTGTTCCAGGAGCTGGTAAAAGTTCCGCCATTAGGG
MF496659.1      TACATAAGCCGATTGTTGTGCATAGTGTCCCAGGAGCTGGTAAAAGCTCCGCGATCAGGG
MF418028.1      TGAATAAACCAATAGTTGTGCATAGTGTTCCAGGTGCCGGTAAAAGTTCCGCTATCAGGG
MH282867.1      TGAATAAACCAATAGTTGTGCATAGTGTTCCAGGTGCTGGTAAAAGTTCTGCTATCAGGG
MK563994.1      TGAATAAACCAATAGTTGTGCATAGCGTTCCAGGTGCTGGTAAAAGTTCTGCTATCAGGG
JQ647830.1      TGAATAAACCAATAGTTGTGCATAGTGTTCCAGGTGCTGGTAAAAGTTCCGCAATCAGGG
MN095414.1      TGAATAAACCAATAGTCGTACATAGCGTTCCAGGCGCTGGTAAAAGTTCCGCAATCAGGG
KU058657.1      TGAATAAACCAATAGTTGTACATAGCGTTCCAGGTGCTGGTAAAAGTTCCGCAATCAGGG
KU896946.1      TGAATAAACCAATAGTTGTACATAGCGTTCCAGGTGCTGGTAAAAGTTCCGCAATCAGGG
MF033144.1      TGAATAAACCAATAGTTGTACATAGCGTTCCAGGTGCTGGTAAAAGTTCCGCAATCAGGG
MF418030.1      TAAATAAACCGATAGTTGTTCATAGTGTCCCGGGAGCTGGTAAAAGTTCTGCAATTCGGG
MF418031.1      TAAATAAACCGATAGTTGTTCATAGCGTCCCGGGGGCTGGTAAAAGTTCTGCAATTCGGG
KP089978.1      TAAATAAACCAATAGTTGTTCATAGTGTCCCGGGAGCTGGCAAAAGTTCTGCAATTCGGG
KU896945.1      TAAATAAACCAATAGTTGTTCATAGTGTCCCGGGAGCTGGTAAAAGTTCCGCAATTCGGG
LC375228.1      TAAATAAACCAATAGTTATTCATAGTGTCCCGGGAGCTGGTAAAAGTTCCGCTATTCGGG
KC430335.1      TAAATAAACCAATAGTTGTTCATAGTGTCCCGGGAGCTGGTAAAAGTTCTGCAATCCGGG
MF418033.1      TAAATAAACCAATAGTTGTTCATAGTGTCCCGGGAGCTGGTAAAAGTTCTGCAATCCGGG
MW331321.1      TAAATAAACCAATAGTTGTTCATAGTGTCCCGGGGGCTGGTAAAAGTTCTGCAATTCGGG
LC375227.1      TAGATAAACCAATAGTTGTTCATAGTGTCCCGGGAGCTGGTAAAAGCTCCGCAATTAGGG
MF375506.1      TAAATAAACCAATAGTTGTTCATAGTGTCCCGGGAGCTGGTAAAAGTTCTGCCATCCGGG
MK442089.1      TAAATAAACCAATAGTTGTTCATAGTGTCCCAGGAGCTGGTAAAAGTTCTGCAATTCGGG
AJ863510.1      TAAATAAACCTATAGTTGTTCATAGTGTCCCGGGTGCTGGTAAGAGTTCCGCGATCAGGG
D00461.1        TAAATAAACCAATAGTTGTTCATAGTGTTCCAGGTGCTGGTAAGAGTTCCGCGATCAGGG
                ******************* ******** ** ** ************** ** **  ***
```

```
MF418029.1      AGCTGTTGAAGTTAGATAGTAGGTTTGAGTGCATTACCCGCGGTCGTCCAGACATTCCGA
KR152654.1      AATTGTTGAAGTTAGATAGTAGGTTTGAGTGTATCACTCGTGGTCGCCCAGACATTCCAA
MF418035.1      AATTGTTGAAGTTAGATAGTAGGTTTGAGTGTATCACCCGTGGTCGCCCAGACATTCCAA
MF418032.1      AGTTACTGAAGTTAGATAGTAGGTTTGAGTGCATCACCCGTGGTAGGCCAGACATACCCA
MF496659.1      AATTACTGAAGTTAGATAGTAGGTTTGAGTGCATTACCCGTGGTCGGCCGGATATACCCA
MF418028.1      AGTTACTGAAGTTAGATAGTAGGTTTGAGTGCATCACCCGCGGCCGGCCAGATATTCCCA
MH282867.1      AGTTGCTGAAGTTAGATAGTAGGTTTGAGTGCATTACCCGCGGCCGGCCAGATATTCCCA
MK563994.1      AGTTACTGAAGTTAGATAGTAGGTTTGAGTGCATAACCCGCGGCCGGCCAGATATTCCCA
JQ647830.1      AGCTACTGAAGTTAGATAGTAGGTTTGAGTGCATCACCCGCGGCCGGCCAGATATTCCCA
MN095414.1      AGCTACTGAAGTTAGATAGTAGGTTTGAGTGCATCACCCGCGGCCGGCCAGATATTCCCA
KU058657.1      AGCTACTGAAGTTAGATAGTAGGTTTGAGTGCATCACCCGCGGCCGGCCAGATATTCCCA
KU896946.1      AGCTACTGAAGTTAGATAGTAGGTTTGAGTGCATCACCCGCGGCCGGCCAGATATTCCCA
MF033144.1      AGCTACTGAAGTTAGATAGTAGGTTTGAGTGCATCACCCGCGGCCGGCCAGATATTCCCA
MF418030.1      AGTTGCTTAAGTTAGATAGTAGATTTGAGTGCATTACTCGCGGTCGGCCAGACATCCCGA
MF418031.1      AGTTGCTTAAGTTAGATAGTAGATTTGAGTGCATTACCCGCGGTCGGCCAGACATCCCGA
KP089978.1      AGTTGCTTAAGTTAGATAGTAGGTTTGAGTGCATCACCCGTGGCCGGCCAGACATCCCGA
KU896945.1      AGTTGCTTAAGTTAGATAGTAGGTTTGAGTGCATTACCCGTGGCCGGCCAGACATCCCGA
LC375228.1      AGTTGCTTAAGTTAGATAGTAGGTTTGAGTGCATTACCCGTGGCCGGCCAGACATCCCGA
KC430335.1      AGTTACTTAAGTTAGATAGTAGGTTTGAGTGCATTACCCGTGGCCGGCCAGACATCCCGA
MF418033.1      AATTGCTTAAGTTAGATAGTAGGTTTGAGTGCATTACCCGTGGCCGGCCAGACATCCCGA
MW331321.1      AGTTGCTTAAGTTAGATAATAGGTTTGAGTGCATTACCCGTGGCCGGCCAGACATCCCGA
LC375227.1      AGTTGCTTAAGTTAGATAGTAGGTTTGAGTGCATTACCCGTGGCCGGCCAGACATCCCGA
MF375506.1      AGTTGCTTAAGTTAGATAGTAGGTTTGAGTGCATTACCCGTGGTCGGCCAGACATCCCAA
MK442089.1      AGTTGCTTAAGTTAGATAGTAGGTTTGAGTGCATTACCCGTGGCCGGCCAGACATCCCGA
AJ863510.1      AATTACTGAAGTTAGATAGTAGGTTTGAGTGCATTACCCGTGGCCGGCCAGACATTCCCA
D00461.1        AATTACTGAAGTTAGATAGTAGGTTTGAGTGCATTACCCGTGGCCGGCCAGACATTCCCA
                **** ** ******************************** ************** ** *

MF418029.1      ATTTGGAAGGGGCTTTTATTAAGGCAGAGCGTAGTGGTGAAAATAAGCTACTGCTGGTTG
KR152654.1      ATTTGGAGGGCGCTTTTATCAAAGCTGAACGTAGCGGTGAAAATAAGCTACTACTGGTTG
MF418035.1      ATTTGGAGGGTGCTTTTATCAAAGCTGAACGTAGCGGTGAAAATAAGCTACTACTGGTTG
MF418032.1      ATCTAGAGGGAGCTTTCATCAAGGCTGAACGCAGTGGTGAGGATAAGTTGCTGTTGGTGG
MF496659.1      ATCTAGAGGGGGCTTTCATTAAGGCCGAGCGTTGTGGTGAGAATAAGTTGTTGTTGGTCG
MF418028.1      ATCTAGAGGGGGCTTTCATCAAGAGCGAACGTAGTGGTGAGAATAAGTTGTTACTGGTTG
MH282867.1      ATCTAGAGGGGGCTTTCATCAAGGGCGAACGTAGTGGTGAGAATAAGTTGTTACTAGTTG
MK563994.1      ATCTAGAGGGGGCTTTCATCAAGGGCGAACGTAGTGGTGAGAATAAGTTGTTACTGGTTG
JQ647830.1      ATCTAGAGGGAGCTTTCATCAAGAGCGAACGTAGTGGTGAGGATAAGCTGTTACTGGTTG
MN095414.1      ATCTAGAGGGTGCTTTCATCAAGGGCGAACGTAGTGGTGAGGATAAGCTGTTGCTGGTTG
KU058657.1      ATCTAGAGGGTGCTTTCATCAAGGGCGAACGTAGTGGTGAGGATAAGCTGTTACTGGTTG
KU896946.1      ATCTAGAGGGTGCTTTCATCAAGGGCGAACGTAGTGGTGAGGATAAGCTGTTGCTGGTTG
MF033144.1      ATCTAGAGGGTGCTTTCATCAAGGGCGAACGTAGTGGTGAGGACAAGCTGTTGCTGGTTG
MF418030.1      ATCTAGAGGGGGCTTTCATCAAGGCTGAGCGCGGTGGGGAGAATAAATTGCTGCTGGTTG
MF418031.1      ATCTTGAGGGGGCTTTCATCAAGGCTGAGCGCGGTGGGGAGAATAAATTGCTGCTGGTCG
KP089978.1      ATCTAGAGGGAGCTTTCATCAAGGCTGAGCGCAGTGGGGAGAATAAATTGCTGCTGGTTG
KU896945.1      ATCTAGAAGGGGCTTTCATCAAGGCTGAGCGTAGTGGGGAGAATAAATTGCTGCTGGTCG
LC375228.1      ATCTAGAGGGGGCTTTCATCAAGGCTGAGCGTAGTGGGGAGAATAAATTGCTGCTGGTTG
KC430335.1      ATCTAGAGGGGGCTTTTATTAAGGCTGAGCGCAGTGGGGAGGATAAATTGCTGCTGGTTG
MF418033.1      ATCTAGAGGGGGCTTTCATTAAGGCTGAGCGCAGTGGGGAGAATAAATTACTGCTGGTTG
MW331321.1      ATCTAGAGGGGGCTTTCATCAAGGCCGAACGTAGTGGGGAGAATAAATTGCTGCTGGTCG
LC375227.1      ATCTAGAGGGGGCTTTCATCAAGGCTGAGCGCAGTGGGGAGAATAAATTGCTGCTGGTTG
MF375506.1      ATCTAGAGGGGGCTTTCATCAAGGCTGAGCGCAGTGGGGAGAATAAATTGCTACTGGTCG
MK442089.1      ATCTAGAGGGGGCTTTCATCAAGGCTGAGCGTGGTGGGGAGAATAAATTGCTGCTGGTTG
AJ863510.1      ATCTAGAGGGAGCTTTCATCAAGGCCGAACGTAGTGGTGAGAGTAAGCTGTTACTGGTAG
D00461.1        ATCTAGAGGGAGCTTTCATCAAGGCCGAACGTAGTGGTGAGAGTAAGCTGTTACTGGTAG
                ********** ************** ** ******** ******** *** * *******

MF418029.1      ATGAGTACATAGAGGGGCCCATTCCAGAGGACGCATTCGCAATCTTCGCAGATCCGCTAC
KR152654.1      ATGAGTACATAGAGGGACCTATCCCAGAGGACGCATTCGCGATCTTCGCAGACCCGCTAC
MF418035.1      ATGAGTACATAGAGGGACCTATTCCAGAGGACGCATTCGCGATCTTCGCAGATCCGCTAC
MF418032.1      ACGAGTATATAGAGGGACCCATTCCGGAGGATGCCTTTGCAATCTTTGCGGATCCACTAC
MF496659.1      ATGAGTACATAGAGGGGCCAATTCCAGAGGATGCCTTTGCGATCTTTGCGGATCCACTAC
MF418028.1      ATGAGTACATAGAGGGGCCCGTTCCGGAGGATGCTTTTGCAATCTTTGCAGATCCACTGC
MH282867.1      ATGAGTACATAGAGGGGCCCGTTCCGGAGGATGCTTTTGCTATCTTTGCAGATCCACTGC
MK563994.1      ATGAGTACATAGAGGGGCCCGTTCCGGAGGATGCTTTTGCTATCTTTGCTGATCCACTGC
JQ647830.1      ACGAGTACATAGAGGGGCCCGTTCCTGAAGACGCTTTTGCAATCTTTGCAGATCCGCTGC
MN095414.1      ACGAGTACATAGAGGGGCCCGTTCCAGAAGACGCTTTTGCAATCTTTGCAGATCCGCTAC
KU058657.1      ACGAGTACATAGAGGGGCCCGTTCCAGAAGACGCTTTTGCAATCTTTGCAGATCCGCTGC
KU896946.1      ACGAGTACATAGAGGGGCCCGTTCCAGAAGACGCTTTTGCAATCTTTGCAGATCCGCTGC
MF033144.1      ACGAGTACATAGAGGGGCCCGTTCCAGAAGACGCTTTCGCAATCTTTGCAGATCCGCTGC
MF418030.1      ACGAGTACATAGAAGGGCCGGTCCCAGAGGACGCCTTTGCAATCTTTGCGGATCCACTTC
MF418031.1      ATGAGTACATAGAAGGGCCGGTCCCAGAGGACGCCTTTGCAATCTTTGCGGATCCACTTC
KP089978.1      ATGAGTACATAGAAGGGCCGGTCCCAGAGGACGCCTTTGCAATCTTTGCAGATCCACTTC
KU896945.1      ATGAGTACATAGAGGGGCCGATCCCAGAGGACGCCTTTGCAATCTTTGCAGATCCACTTC
LC375228.1      ATGAGTACATAGAAGGGCCGGTCCCAGAGGACGCCTTTGCAATCTTTGCAGATCCACTTC
KC430335.1      ATGAGTACATAGAAGGGCCGGTCCCAGAGGACGCCTTTGCAATCTTTGCAGATCCACTTC
MF418033.1      ATGAGTACATAGAAGGGCCAGTCCCAGAGGACGCCTTTGCAATCTTTGCAGATCCACTTC
MW331321.1      ATGAGTACATAGAAGGGCCGGTTCCAGAGGACGCCTTTGCTATCTTCGCTGATCCACTTC
LC375227.1      ATGAGTACATAGAAGGGCCGGTCCCAGAGGACGCCTTTGCAATCTTTGCAGATCCACTTC
MF375506.1      ATGAGTACATAGAAGGGCCGGTCCCAGAGGACGCCTTTGCAATCTTTGCTGATCCACTTC
MK442089.1      ATGAGTACATAGAAGGGCCGGTCCCAGAGGATGCCTTTGCAATCTTTGCTGATCCACTTC
AJ863510.1      ATGAGTACATAGAAGGGCCCATTCCAGAGGACGCCTTTGCAATCTTCGCAGATCCGCTTC
D00461.1        ATGAGTACATAGAAGGGCCCATTCCAGAGGACGCCTTTGCAATCTTCGCAGATCCGCTTC
                ************* ***** ** ******** ** ************** ***** ** *
```

```
MF418029.1      AGAGCACTGCCGTGAGCCCACACAGGGCGCACTTCATCAAAACACTAAGCCATCGCTTTG
KR152654.1      AGAGCACAGCTGTGAGTCCGCATAGGGCGCACTTCATTAAGACGCTAAGCCATCGCTTTG
MF418035.1      AGAGCACTGCTGTGAGTCCGCACAGGGCGCACTTCATCAAGACACTAAGCCATCGCTTTG
MF418032.1      AGAGCACTGCCGTAAGTCCACATAGAGCGCATTTCATCAAGACACTAAGTCATCGCTTTG
MF496659.1      AAAGTACCGCTGTGAGTCCACATAGAGCACACTTCATTAAAACACTAAGCCATCGCTTTG
MF418028.1      AGAGCACAGCTGTTAGCCCATATAGAGCGCATTTCATCAAAACACTAAGTCATCGCTTTG
MH282867.1      AGAGCACAGCTGTTAGTCCATATAGAGCGCATTTCATCAAAACACTAAGTCATCGCTTTG
MK563994.1      AGAGCACAGCTGTTAGCCCATATAGAGCGCATTTCATCAAAACACTAAGTCATCGCTTTG
JQ647830.1      AGAGCACAGCAGTTAGCCCATATAGAGCGCATTTCATCAAAACACTAAGCCATCGCTTTG
MN095414.1      AGAGCACAGCTGTTAGCCCATATAGAGCGCATTTCATCAAAACACTAAGCCATCGCTTTG
KU058657.1      AGAGCACAGCTGTTAGCCCATATAGAGCGCATTTCATCAAAACACTAAGCCATCGCTTTG
KU896946.1      AGAGCACAGCTGTTAGCCCATATAGAGCGCATTTCATCAAAACACTAAGCCATCGCTTTG
MF033144.1      AGAGCACAGCTGTTAGCCCATATAGAGCGCATTTCATCAAAACACTAAGCCATCGCTTTG
MF418030.1      AAAGTACCGCTGTGAGCCCATACCGAGCACACTTCATCAAGACATTGAGCCATCGTTTTG
MF418031.1      AAAGCACCGCTGTGAGCCCATACCGAGCACACTTCATCAAGACATTGAGCCATCGTTTTG
KP089978.1      AGAGTACCGCTGTGAGCCCATACCGGGCACACTTCATCAAAACATTGAGCCATCGCTTTG
KU896945.1      AGAGTGCCGCTGTGAGTCCATACCGGGCGCACTTCATCAAAACATTGAGCCATCGCTTTG
LC375228.1      AGAGTACCGCTGTGAGCCAATACCGGGCGCACTTCATCAAAACATTGAGCCATCGCTTCG
KC430335.1      AGAGCACCGCTGCGAGCCCATACCGAGCGCACTTCATCAAAACATTGAGCCATCGTTTCG
MF418033.1      AGAGTACCGCTGTGAGCCCATACCGAGCACACTTCATCAAAACATTGAGCCATCGCTTCG
MW331321.1      AAAGTACCGCTGTGAGCCCATACCGGGCACACTTCATCAAAACATTGAGCCATCGCTTTG
LC375227.1      AGAGTACTGCTGTGAGCCCGTACCGGGCACACTTCATCAAAACATTGAGCCATCGCTTTG
MF375506.1      AGAGTACCGCTGTGAGCCCATACCGGGCAAACTTCATCAAAACATTGAGCCATCGCTTTG
MK442089.1      AGAGTACCGCTGTGAGCCCATACCGGGCACACTTCATCAAAACATTGAGCCATCGCTTTG
AJ863510.1      AGAGCACAGCCGTCAGTCCACACAGAGCGCATTTCATCAAAACACTAAGCCATCGCTTTG
D00461.1        AGAGCACAGCCGTCAGTCCACACAGAGCGCATTTCATCAAAACACTAAGCCATCGCTTTG
                **** ** ***** ********  * ** ** ************ * *************

MF418029.1      GCAAGTGCACAGCTGAGCTATTGAGGGATTTGGGTTGGGACGTGCAGGCGGAGGGTCAAG
KR152654.1      GCAAGAGTACAGCTGGGTTATTGAGGGATTTAGGTTGGGACGTGCAAGCAGAGGGTCAGG
MF418035.1      GCAAGTGTACAGCTGAGTTATTGAGGGATTTGGGTTGGGACGTGCAGGCAGAGGGTCAGG
MF418032.1      GTGAGTGTACGGCCACACTCTTAAGGGATTTGGGTTGGGATGTGCAGGCTGAAGGTCAGG
MF496659.1      GCAAGTGTACTGCTACACTCTTAAGGGATTTGGGTTGGGACGTGCAGGCTGAAGGTCAGG
MF418028.1      GCAAGTGTACTGCTTCGCTCTTGAGGGATTTGGGTTGGGACGTGCAAGCTGAAGGTCAGG
MH282867.1      GCAAGTGTACTGCTTCGCTCTTGAGAGATTTGGGTTGGGACGTGCAAGCTGAAGGTCAGG
MK563994.1      GCAAGTGTACTGCTTCGCTCTTGAGAGATTTGGGTTGGGACGTGCAAGCTGAAGGTCAGG
JQ647830.1      GCAAGTGTACTGCTTCGCTCTTGAGGGATTTGGGTTGGGACGTGCAAGCTGAAGGTCAGG
MN095414.1      GCAAGTGTACTGCTTCGCTCTTAAGGGACTTGGGTTGGGACGTGCAAGCTGAGGGTCAGG
KU058657.1      GCAAGTGTACTGCTTCGYTCTTAAGGGATTTGGGTTGGGACGTGCAAGCTGAAGGTCAGG
KU896946.1      GCAAGTGTACTGCTTCGCTCTTAAGGGATTTGGGTTGGGACGTGCAAGCTGAGGGTCAGG
MF033144.1      GCAAGTGCACTGCTTCGCTCTTAAGGGATTTGGGTTGGGACGTGCAAGCTGAGGGTCAGG
MF418030.1      GCAAGTGTACCGCTTCCCTTTTGAGAGATTTGGGTTGGGACGTACAGGCAGAAGGTCAAG
MF418031.1      GCAAGTGTACCGCTTCCCTTTTGAGAGATTTGGGTTGGGACGTACAGGCAGAGGGTCAAG
KP089978.1      GCAAGTGTACCGCTTCCCTTTTGAGAGATTTGGGTTGGGACGTGCAGGCAGAAGGTCAAG
KU896945.1      GCAAGTGTACCGCTTCCCTTTTGAGAGATTTGGGTTGGGACGTGCAGGCAGAAGGTCAAG
LC375228.1      GCAAGTGTACCGCTTCCCTTTTGAGAGATTTGGGTTGGGACGTGCAGGCAGAAGGTCAAG
KC430335.1      GCGAGTGTACCGCTTCCCTTTTGAGAGATTTGGGCTGGGACGTACAGGCAGAAGGTCAAG
MF418033.1      GCAAGTGTACTGCTTCCCTTTTGAGAGATTTGGGTTGGGACGTACAGGCAGAGGGTCAAG
MW331321.1      GCAAGTGTACCGCTACCCTTTTGAGAGATTTGGGTTGGGACGTACAGGCAGAAGGTCAAG
LC375227.1      GCAAGTGTACCGCTTCCCTTTTGGTAGATTTGGGTTGGGACGTACAGGCAGAAGGTCAAG
MF375506.1      GCAAGTGTACCGCTTCCCTTTTGAGAGATTTGGGTTGGGACGTACAGGCAGAAGGTCAAG
MK442089.1      GCAAGTGTACCGCTTCCCTTTTGAGAGATTTGGGTTGGGACGTACAGGCAGAAGGTCAAG
AJ863510.1      GCAAGTGTACTGATTCACTCTTGAGAGATTTGGGTTGGGACGTGCAAGCTGAAGGTCAGG
D00461.1        GCAAGTGTACTGATTCACTCTTGAGAGATTTGGGTTGGGACGTGCAAGCTGAAGGTCAGG
                ********** ******** ***** ***************** ** ** ******** *

MF418029.1      ATTCAGTTCAGATTGCAGATATTTTCACGGTCGATCCTAGGGAGACCATAATCTACTTTG
KR152654.1      ATTTGGTTCAGGTTGCAGATATCTTCACGGTTGATCCTAGAGAGACCGTAGTCTATTTTG
MF418035.1      ATTTGGTTCAGGTTGCAGATATCTTCACGGTTGATCCTAGAGAGACCGTAGTCTATTTTG
MF418032.1      ATTCAGTCCAAATAGCCGACATTTTCACAGTTGACCCTAGAGAAAAGGTTGTCTACTTTG
MF496659.1      ACTCAGTCCAAATTGCTGATATTTTCACAGTTGACCCTAGGGAAACGATTGTCTACTTTG
MF418028.1      ATTCAGTACAAATTGCCGATATCTTCACGGTTGACCCTAAAGAAACAGTTGTCTACTTTG
MH282867.1      ATTCAGTGCAAATTGCCGATATCTTCACGGTTGACCCTAAAGAAACAGTTGTTTACTTTG
MK563994.1      ATTCAGTACAAATTGCCGATATCTTCACGGTTGACCCTAAAGAAACAGTTGTTTACTTTG
JQ647830.1      ATTCAGTACAAATTGCCGATATCTTCACGGTTGACCCTAAAGAAACAGTTGTTTACTTTG
MN095414.1      ATTCAGTACAAATTGCCGATATCTTCACGGTTGACCCTAAAGAAACAGTTGTTTACTTTG
KU058657.1      ATTCAGTACAAATTGCCGATATCTTCACGGTTGACCCTAAAGAAACAGTTGTTTACTTTG
KU896946.1      ATTCAGTACAAATTGCCGATATCTTCACGGTTGACCCTAAAGAAACAGTTGTTTACTTTG
MF033144.1      ATTCAGTACAAATTGCCGATATCTTCACGGTTGACCCTAAAGAAACAGTTGTTTACTTTG
MF418030.1      ATTCAGTTCAAATCGCCGATATCTTCACAGTTGACCCCAGGGATACTATTGTGTACTTCG
MF418031.1      ATTCAGTTCAAATCGCCGATATCTTCACAGTTGACCCTAGGGATACTATTGTGTACTTCG
KP089978.1      ATTCAGTTCAAATCGCGGATATCTTCACAGTCGACCCTAGGGACACTATTGTGTACTTCG
KU896945.1      ATTCAGTCCAAATTGCAGATATCTTCACAGTTGACCCTAGGGATACTATTGTGTACTTCG
LC375228.1      ATTCAGTTCAAATTGCAGATATCTTCACAATTGATCCTAGGAATACTATTGTGTACTTCG
KC430335.1      ATTCAGTTCAAATCGCAGATATCTTCACAGTTGACCCTAGGGATACTATTGTGTACTTCG
MF418033.1      ATTCAGTTCAAATCGCAGATATATTCACAGTTGACCCTAGGGATACTATTGTGTACTTCG
MW331321.1      ATTCAGTTCAAATCGCAGATATCTTCACAGTTGACCCTAGGGATACTATTGTGTACTTCG
LC375227.1      ATTCAGTTCAAATCGCAGATATCTTCACAGTTGACCCTAGGGATACTATTGTGTACTTCG
MF375506.1      ATTCAGTTCAAATTGCAGATATCTTCACAGTTGACCCTAGGGATACTATTGTGTACTTTG
MK442089.1      ATTCAGTTCAAATTGCAGATATCTTCACAGTTGACCCTAGGGATACTATTGTGTACTTCG
AJ863510.1      ATTCAGTTCAAATCGCTGATATCTTCACGGTCGACCCCAGAGAAACAATTGTTTACTTTG
D00461.1        ATTCAGTTCAAATCGCTGATATCTTCACGGTCGACCCCAGAGAAACAATTGTTTACTTTG
                ******* ***** ** *********** *********** ** **  ********** *
```

```
MF418029.1      AGCCAGAAGTTGGAGAGTTGTTGAGGAACCACGGCGTTGAGTCGAGCTGCATTGGCGAGG
KR152654.1      AGCCAGAGGTTGGAGAATTGTTGAGGAGCCACGGGGTTGAGGCGAGCTGCATTGGAGAGG
MF418035.1      AGCCAGAGGTTGGAGAATTGTTGAGGAGCCACGGTGTTGAGGCAAGCTGCATTGGAGAGG
MF418032.1      AGCCAGAGGTTGGAGAGTTGCTAAGGAGCCACGGTGTGGAAGCAAGCTGCATCGGCGAAG
MF496659.1      AGCCGGAAGTTGGAGAGTTACTGAGGAACCACGGTGTTGAAGCAAGTTGCATTGGTGAAG
MF418028.1      AGCCGGAAGTCGGAGAGTTGTTGAGGAACCACGGCGTTGAGGCAAGTTGCATTGGTGAGG
MH282867.1      AGCCGGAAGTCGGAGAGTTGTTGAGGAACCACGGCGTTGAGGCAAGTTGCATTGGTGAGG
MK563994.1      AGCCGGAAGTCGGAGAGTTGTTGAGGAACCACGGCGTTGAGGCAAGTTGCATTGGTGAGG
JQ647830.1      AGCCGGAAGTCGGAGAGTTGTTGAGGAATCACGGCGTTGAGGCAAGTTGCATTGGTGAGG
MN095414.1      AGCCGGAAGTCGGAGAGTTGCTGAGGAACCACGGCGTTGAGGCGAGTTGCATTGGTGAGG
KU058657.1      AGCCGGAAGTCGGAGAGTTGTTGAGGAACCACGGCGTTGAGGCGAATTGCATTGGTGAGG
KU896946.1      AGCCGGAAGTCGGAGAGTTGTTGAGGAACCACGGCGTTGAGGCGAGTTGCATTGGTGAGG
MF033144.1      AGCCGGAAGTCGGAGAGTTGTTGAGGAACCACGGCGTTGAGGCGAGTTGCATTGGTGAGG
MF418030.1      AGCCAGAAGTTGGAGAGTTACTAAGGAGCCACGGTGTCGAGGCGAGCTGCATTGGTGAGG
MF418031.1      AGCCAGAAGTTGGAGAGTTACTGAGGAGCCACGGTGTCGAGGCGAGCTGCATTGGTGAGG
KP089978.1      AGCCAGAGGTTGGAGAGTTACTGAGGAGCCACGGTGTCGAGGCGAGCTGCATTGGTGAGG
KU896945.1      AGCCGGAAGTTGGAGAGTTACTGAGGAGCCACGGTGTCGAGGCAAGCTGCATTGGTGAGG
LC375228.1      AGCCGGAAGTTGGAGAGTTACTGAGGAGCCACGGTGTCGAGGCGAGCTGCATTGGTGAGG
KC430335.1      AACCAGAAGTTGGGGAGTTACTGAGGAGCCACGGAGTCGAGGCGAGCTGCATTGGTGAGG
MF418033.1      AGCCAGAAGTTGGAGAGTTACTGAGGAGCCACGGTGTCGAGGCGAGCTGCATTGGTGAAG
MW331321.1      AGCCAGAAGTTGGAGAGTTACTGAGGAGCCACGGTGTCGAAGCGAGCTGCATTGGTGAGG
LC375227.1      AGCCAGAAGTTGGAGAGTTACTGAGGAGCCACGGTGTCGAGGCGAGCTGCATTGGTGAGG
MF375506.1      AGCCAGAAGTTGGAGAATTACTGAGGAGCCACGGTGTCGAGGCGAGTTGCATTGGTGAGG
MK442089.1      AGCCGGAAGTTGGAGAGTTGCTGAGGAGCCACGGCGTCGAGGCGAGCTGCATTGGTGAGG
AJ863510.1      AGCCGGAAGTTGGTGAGTTGCTGAGGAGTCACGGAGTCGAGGCAAGCTGCATCGGTGAGG
D00461.1        AGCCGGAAGTTGGTGAGTTGCTGAGGAGTCACGGAGTCGAGGCAAGCTGCATCGGTGAGG
                **** ******** ** **  ************* ** ***** ** *************

MF418029.1      TGCGAGGGGCTACTTTTGAACACGTAACTTTTGTCACATCTGAGAACAGCCCTCTAATTG
KR152654.1      TGCGAGGCGCCACTTTTGAGCACGTAACATTTGTTACATCTGAGAACAGCCCTCTAATTG
MF418035.1      TGCGAGGCGCCACTTTTGAGCACGTAACATTTGTTACTTCTGAGAACAGCCCTCTAATTG
MF418032.1      TGCGCGGGGCTACTTTTGAGCACGTAACATTCGTCACTTCTGAGAACAGTCCTCAAATTG
MF496659.1      TGCGTGGAGCTACCTTTGAGCACGTAACATTTGTAACATCTGAAAACAGCCCTCAAATTG
MF418028.1      TGCGAGGAGCTACTTTTGAGCACGTAACGTTCGTCACTTCTGAAAACAGCCCACTAGTTG
MH282867.1      TGCGAGGAGCTACTTTTGAGCACGTAACGTTCGTCACTTCTGAAAACAGCCCACTAGTTG
MK563994.1      TGCGAGGAGCTACTTTTGAGCACGTGACGTTCGTCACTTCTGAAAACAGCCCACTAGTTG
JQ647830.1      TGCGAGGAGCTACCTTTGAGCACGTGACGTTCGTCACTTCTGAAAACAGCCCACTAGTTG
MN095414.1      TGCGAGGAGCTACTTTTGAGCACGTGACGTTCGTCACTTCTGAAAACAGCCCACTAGTTG
KU058657.1      TGCGAGGAGCTACTTTTGAGCACGTGACGTTCGTCACTTCTGAAAACAGCCCACTAGTTG
KU896946.1      TGCGAGGAGCTACTTTTGAGCACGTGACGTTCGTCACTTCTGAAAACCGCCCACTAGTTG
MF033144.1      TGCGAGGAGCTACTTTTGAGCACGTGACGTTCGTCACTTCTGAAAACAGCCCACTAGTTG
MF418030.1      TGCGCGGGGCCACTTTTGAACACGTAACCTTCGTCACCTCTGAGAACGGCCCACTGGTTG
MF418031.1      TGCGCGGGGCCACTTTTGAACACGTGACCTTCGTCACTTCTGAGAACGGCCCACTGGTTG
KP089978.1      TGCGCGGGGCTACTTTTGAACACGTAACTTTCGTCACTTCTGAGAGCGGCCCACTGATTG
KU896945.1      TGCGCGGGGCCACTTTCGAACACGTGACCTTCGTCACTTCTGAGAACGGTCCGCTGGTTG
LC375228.1      TGCGCGGGGCCACTTTCGAACACGTAACCTTCGTCACTTCTGAGAACGGTCCGCTGGTTG
KC430335.1      TGCGCGGGGCTACTTTCGAACACGTGACCTTCGTCACTTCTGAGAACGGCCCACTGGTTG
MF418033.1      TGCGCGGGGCTACTTTCGAACACGTGACTTTCGTCACTTCTGAGAACGGCCCACTGGTTG
MW331321.1      TGCGCGGGGCTACTTTCGAACACGTGACCTTCGTCACTTCTGAGAACGGCCCACAGGTTG
LC375227.1      TGCGCGGGGCTACTTTCGAACACGTGACCTTCGTCACTTCTGAGAACGGCCCACTGATTG
MF375506.1      TGCGCGGGGCTACTTTCGAACACGTGACCTTCGTCACTTCTGAGAACGGCCCACTGGTTG
MK442089.1      TGCGCGGGGCCACTTTCGAACACGTAACCTTCGTCACTTCTGAGAACAGCCCACTGGTTG
AJ863510.1      TGCGTGGGGCCACTTTTGAGCACGTAACGTTTGTCACATCTGAAAATAGCCCATTGATTG
D00461.1        TGCGTGGGGCCACTTTTGAGCACGTAACGTTTGTCACATCTGAAAATAGCCCATTGATTG
                **** ** ** ***** ** ***** ** ************** *** ******* ****

MF418029.1      ATAAGGCCGCCGCATTCCAGTGTTTGACACGGCACACTAAGAGTTTGCTAATCTTGTGCC
KR152654.1      ATAAGGCCGCCGCATTTCAGTGTTTGACGCGGCACTCCAAGAGTCTGCTAATATTGTGCC
MF418035.1      ATAAGGCCGCCGCATTTCAGTGTTTGACGCGGCACACTAAGAGTCTGCTAATATTGTGCC
MF418032.1      ACAGGGCCGCAGCATTTCAGTGTTTGACTAGGCACACTAAGAGCTTGCTCGTACTGTGCC
MF496659.1      ATAAAGCTGCAGCTTTTCAGTGCTTAACTAGGCACTCTAAGAGTTTACTCATATTGTGCC
MF418028.1      ATAAGGCCGCCGCCTTTCAGTGCTTAACGAGGCACACCAAGAGCTTGCTCATACTGTGCC
MH282867.1      ATAAGGCCGCCGCCTTTCAGTGCTTAACGAGGCACACCAAGAGCTTGCTCATACTGTGCC
MK563994.1      ATAAGGCCGCCGCCTTTCAGTGCTTAACGAGGCACACCAAGAGCTTGCTCATACTGTGCC
JQ647830.1      ATAAGGCCGCCGCCTTCCAGTGCTTAACGAGGCACACCAAGAGCTTGCTCATACTGTGCC
MN095414.1      ATAAGGCCGCCGCCTTCCAGTGCTTAACGAGGCACACCAAGAGCTTGCTCATACTGTGCC
KU058657.1      ATAAGGCCGCCGCCTTCCAGTGCTTAACGAGGCACACCAAGAGCTTGCTCATACTGTGCC
KU896946.1      ATAAGGCCGCCGCCTTCCAGTGCTTAACGAGGCACACCAAGAGCCTGCTCATACTGTGCC
MF033144.1      ATAAGGCCGCCGCCTTCCAGTGCTTAACGAGGCACACCAAGAGCTTGCTCATACTGTGCC
MF418030.1      ATAAGGCTGCTGCTTTTCAGTGCTTAACGAGGCATACTAAGAGCTTGCTCATATTGTGCC
MF418031.1      ATAAGGCTGCTGCTTTTCAGTGCTTAACGAGGCACACTAAGAGCTTGCTCATTATGCC
KP089978.1      ATAAGGCAGCTGCTTTTCAGTGCTTGACGAGGCACACTAAGAGCTTGCTCATATTGTGCC
KU896945.1      ATAAGGCTGCTGCTTTTCAGTGCTTGACGAGGCACACCAAGAGCTTGCTCATATTGTGCC
LC375228.1      ATAAGGCTGCTGCTTTTCAGTGCTTGACGAGGCACACCAAGAGCTTGCTCATATTGTGCC
KC430335.1      ATAAAGCTGCCGCTTTTCAATGCTTAACGAGGCACACTAAGAGCTTGCTCATATTGTGCC
MF418033.1      ATAAGGCTGCTGCTTTTCAGTGCTTAACGAGGCACACTAAGAGCTTGCTCATATTGTGCC
MW331321.1      ATAAGGCTGCTGCTTTTCAGTGCTTGACGAGGCACACTAAGAGCTTGCTCATATTGTGCC
LC375227.1      ATAAGGCTGCTGCTTTTCAGTGCTTAACGAGGCACACTAAGAGCTTGCTCATCTTGTGCC
MF375506.1      ATAAGGCTGCTGCTTTTCAGTGCTTGACGAGGCACACTAAGAGCTTGCTCATATTGTGCC
MK442089.1      ATAAGGCTGCTGCTTTTCAGTGCTTGACGAGGCACACTAAGAGCTTGCTCATATTGTGCC
AJ863510.1      ATAAGGCCTCTGCATTTCAGTGCTTAACGAGGCACACCAAGAGCTTACTCATATTGTGCC
D00461.1        ATAAGGCCTCTGCATTTCAGTGCTTAACGAGGCACACCAAGAGCTTACTCATATTGTGCC
                ******* ** ** *********** *********** **********************
```

```
MF418029.1      CAGATGCCACTTACACCGCCGCCTAATTACACAGGGTTGTACACAGCAGCGGCTTTGGGG
KR152654.1      CAGATGCCACTTACACCGCCGCCTAATTACACAGGGTTATACACAGCAGCGGCCTTAGGG
MF418035.1      CAGATGCCACTTACACCGCCGCCTAATTACACAGGGTTATACACAGCAGCGGCCTTAGGG
MF418032.1      CAGATGCCACTTACGCCACCGCCTAATTACACAGGGTTATATATAGCGGCAGCGCTAGGT
MF496659.1      CAGATGCCACTTACACCGCCGCCTAATTACACAGGGTTATATATTGCTGCAGCGCTTGGC
MF418028.1      CTGATGCCACTTACACCGCCGCCTAATTACACAGGGTTATACATCGCGGCAGCGCTAGGT
MH282867.1      CTGATGCCACTTACACCGCCGCCTAATTACACAGGGTTATACATCGCGGCAGCACTAGGT
MK563994.1      CTGATGCCACTTACACCGCCGCCTAATTACACAGGGTTATACATCGCGGCAGCACTAGGT
JQ647830.1      CAGATGCCACTTACACCGCCGCCTAATTACACAGGGTTATACATCGCGGCGGCACTAGGT
MN095414.1      CAGATGCCACTTACACCACCGCCTAATTACACAGGGTTATACATCGCGGCGGCACTAGGT
KU058657.1      CAGATGCCACTTACACCACCGCCTAATTACACAGGGTTATACATCGCGGCGGCACTAGGT
KU896946.1      CAGATGCCACTTACACCACCGCCTAATTACACAGGGTTATACATCGCGGCGGCACTAGGT
MF033144.1      CAGATGCCACTTACACCACCGCCTAATTACACAGGGTTATACATCGCGGCGGCACTAGGT
MF418030.1      CTGATGCCACTTACACCGCCGCCTAACTACACAGGGTTATACATTGCAGCGGTTTTAGGA
MF418031.1      CTGATGCCACTTACACCGCCGCCTAACTACACAGGGTTATACATCGCAGCGGCTTTAGGA
KP089978.1      CTGATGCCACTTACACCGCCGCCTAACTACACAGGGTTATATATTGCAGCGGCTTTAGGA
KU896945.1      CTGATGCCACTTACACCGCCGCCTAACTACACAGGGTTATACATTGCTGCAGCTTTGGGA
LC375228.1      CTGATGCCACTTACACCGCCGCCTAACTACACAGGGTTATACATTGCTGCAGCTTTGGGA
KC430335.1      CTGATGCCACTTACACCGCCGCCTAACTACACAGGGTTATACATTGCAGCGGCTTTAGGA
MF418033.1      CTGATGCCACTTACACCGCCGCCTAACTACACAGGGTTATACATTGCAGCGGCTTTAGGA
MW331321.1      CTGATGCCACTTACACCGCCTCCTAACTACACAGGGTTATACATTGCAGCGGCTTTAGGA
LC375227.1      CTGATGCCACTTACACCGCCGCCTAACTACACAGGGTTATACATTGCAGCGGCTTTAGGA
MF375506.1      CTGATGCCACTTACACCGCCGCCTAACTACACAGGGTTATACATTGCAGCGGCTTTAGGA
MK442089.1      CTGATGCCACTTACACCGCCGCCTAACTACACAGGGTTATACATTGCAGCGGCTTTGGGA
AJ863510.1      CTGATGCCACTTACACCGCCGCCTAATTACACAGGGTTATACATTGCGGCAGCGCTTGGT
D00461.1        CTGATGCCACTTACACCGCCGCCTAATTACACAGGGTTATACATTGCGGCAGCGCTTGGT
                * ************************* *********** ** *   ** ** *    * **
```

PVS

CP ORF

```
MF418029.1      TAAAGCACCTTTAGGTTCACAGGTAAGAGTTCGAATAAACTACCTCACGAATAGAATGCC
KR152654.1      TAAAGCACCTTTAGGTTCACAGGTAAGAGTTCGAATAAACTACCTCACGAATAGAATGCC
MF418035.1      TAAAGCACCTTTAGGTTCACAGGTAAGAGTTCGAATAAACTACCTCACGAATAGAATGCC
MF418032.1      TGAAACACCTTTAGGTTCACAGGTAAAAGCTCGAATAAACTATCTCAAAGCAAGAATGCC
MF496659.1      TGAAACACCTTTAGGTTCACAGGTAAAGGCTCGAATTAACTATCTCACAGTAAGAATGCC
MF418028.1      TGAAACACCTTTAGGTTCACAGGTAAAAGCTCGAATAAACTGTTTCACAGCGAGAATGCC
MH282867.1      TGAAACACCTTTAGGTTCACAGGTAAAAGCTCGAACAAACTGTTTCACAGCGAGAATGCC
MK563994.1      TGAAACACCTTTAGGTTCACAGGTTAAAGCTCGAATAAACTGTTTCACAGCGAGAATGCC
JQ647830.1      TGAAACACCTTTAGGTTCACAGGTAAAAGCTCGAATAAACTGTCTCACAGCGAGAATGCC
MN095414.1      TGAAACACCTTTAGGTTCACAGGTAAAAGCTCGAATAAACTGCTTCACAGAGAGAATGCC
KU058657.1      TGAAACACCTTTAGGTTCACAGGTAAAAGCTCGAATAAACTGTTTCACAGAGAGAATGCC
KU896946.1      TGAAACACCTTTAGGTTCACAGGTAAAAGCTCGAATAAACTGTTTCACAGAGAGAATGCC
MF033144.1      TGAAACACCTTTAGGTTCACAGGTAAAAGCTCGAATAAACTGTTTCACAGAGAGAATGCC
MF418030.1      TGAAGCACCTTTAGGTTCACAGGTAAGAGTTCGAAGAAACTGTCCCACAGAGAAAATGCC
MF418031.1      TGAAGCACCTTTAGGTTCACAGGTAAGAGTTCGAAGAAACTGTCCCACAGAGAAAATGCC
KP089978.1      TGAAGCACCTTTAGGTTCACAGGTAAGAGTTCGAAGAAACTGTCCCACAGAGAAAATGCC
KU896945.1      TGAAGCACCTTTAGGTTCACAGGCAAGAGTTCGAAGAAACTGCCCCACAGAGAAAATGCC
LC375228.1      TGAAGCACCTTTAGGTTCACAGGTAAGAGTTCGAAGAAACTGTCCCACAGAGAAAATGCC
KC430335.1      TGAAGCACCTTTAGGTTCACAGGTAAGAGTTCGAAGTAACTGTCCCACAGAGAAAATGCC
MF418033.1      TGAAGCACCTTTAGGTTCACAGGTAAGAGTTCGAAGAAACTGTCCCACAGAGAAAATGCC
MW331321.1      TGAAGCACCTTTAGGTTCACAGGTAAGAGTTCGAAGAAACTGTCCCACAGAGAAAATGCC
LC375227.1      TGAAGCACCTTTAGGTTCACAGGTAAGAGTTCGAAGAAACTGTCCCACAGAGAAAATGCC
MF375506.1      TGAAGCACCTTTAGGTTCACAGGTAAGAGTTCGAAGAAACTGTCCCACAGAGAAAATGCC
MK442089.1      TGAAGCACCTTTAGGTTCACAGGTAAGAGTTCGAAGAAACTGTCCCACAGAGAAAATGCC
AJ863510.1      TGAAACACCTTTAGGTTCACAGGTAAAAGCTCGAATATACAGTCTCACAGCAAGAATGCC
D00461.1        TGAAACACCTTTAGGTTCACAGGTAAAAGCTCGAATATACAGTCTCACAGCAAGAATGCC
                * ** *****************   *   * *****    **      **      * ******

MF418029.1      GCCTAAACCAGATCCAAACAGCTCAGGCGAAGCGCCACCGGCTATGCCACCAGCACTACC
KR152654.1      GCCCAAACCAGATCCCTCCAGCTCAGGCGAGGCGCCGCCAGCTATGCCGCCAGCACCACC
MF418035.1      GCCCAAACCAGATCCCTCCAGCTCAGGCGAGGCGCCGCCAGCTATGCCGCCAGCACCACC
MF418032.1      GCCTAAACCAGATCCATCTAGTTCAGGGGAAGCACCACAAGCAATGCCATATGCGCCACC
MF496659.1      ACCTAAACCAGATCCATCCAGTTCAGGGGAATCCCCAATAGCAATGCCGCCCGCTCCTCC
MF418028.1      GCCTAAACCAGATCCTTCTAGTTCAGGGGAAGCGCCACAAGCTATGCCACCTGCACCACC
MH282867.1      GCCTAAACCAGATCCTTCTAGTTCAGGGGAAGCGCCACAAGCTACGCCACCTGCACCACC
MK563994.1      GCCTAAACCAGATCCTTCTAGTTCAGGGGAAGCGCCACAAGCTACGCCACCTGCACCACC
JQ647830.1      GCCTAAACCAGATCCTTCTAGTTCAGGGGAAGCGCCACAAGCTATGCCACCTGCACCACC
MN095414.1      GCCTAAACCAGATCCTTCTAGTTCAGGGGAAGTACCACAAGCTATGCCACCTGCACCACC
KU058657.1      GCCTAAACCAGATCCTTCTAGTTCAGGGGAAGTGCCACAAGCTATGCCACCTGCACCACC
KU896946.1      GCCTAAACCAGATCCTTCTAGTTCAGGGGAAGTGCCACAAGCTATGCCACCTGCACCACC
MF033144.1      GCCTAAACCAGATCCTTCTAGTTCAGGGGAAGTGCCACAAGCTATGCCACCTGCACCACC
MF418030.1      GCCCAAACCGGATCCAACAAGCTCAGGAGTGACACCACAAGCACCGCCGCTTGTGCCGCC
MF418031.1      GCCCAAACCGGATCCGACAAGCTCAGGAGAGACACCACAAGCGCTGCCGCTTGTGCCGCC
KP089978.1      GCCTAAACCAGATCCGACGAGCTCAGGAGAGACACCGCAAGCAATACCGCTTGCGCCGCC
KU896945.1      GCCCAAACCGGATCCGACAAGCTCAGGAGAGACACCACAAACTGTGCCGCTTGCGCCGCC
LC375228.1      GCCCAAACCGGATCCAACAAGCTCAGGAGAGACACCACAAACTATACCGCTTGTGCCGCC
KC430335.1      GCCCAAACCGGATCCGACGAGCTCAGGAGAGGCACCACAAGCTATACCACTTGCGCCACC
MF418033.1      GCCCAAACCGGATCCGACAAGCTCAGGAGAGACACCACAAGCCATACCGCTTGCACCGCC
MW331321.1      GCCCAAACCGGATCCGACGAGCTCAGGAGAGACACCACAAGCAATGCCGCCTGCGCCGCC
LC375227.1      GCCCAAACCGGATCCGACAAGCTCAGGAGAGACACCACAAACTATACCGCTTGTGCCGCC
MF375506.1      GCCCAAACCGGATCCAACAAGCTCAGGAGAGACACCACAAGCAATACCGCTTGCGCCGCC
MK442089.1      GCCCAAACCGGATCCAACAAGCTCAGGAGAGACACCACAAGCAATACCGCTTGCGCCGCC
AJ863510.1      GCCTAAACCAGATCCATCTAGCTCAGGGGAAGCACCACAAGCGATGCAACCTGCACCACC
D00461.1        GCCTAAACCAGATCCATCTAGCTCAGGGGAAGCACCACAAGCGATGCAACCTGCACCACC
                *** ***** *****   * ** ***** **   * *******  ** ** * *** ** **
```

```
MF418029.1      TCCGCGTCATGCGGAAGAGCACAAAGTCAGTTCGGCCCAAGGGCCAGGGCAAAATGAAGA
KR152654.1      TCCGCGTTTCGCGGAAGAGCACAAAGCAAGTTCGACTGAAGGGTCAGGGCAAAATGAGGA
MF418035.1      TCCGCGTTTTGCGGAAGAGCACAAAGTGAGTTCGACTCAAGGGCCAAGGCAAAATGAGGA
MF418032.1      GCCGAGAGCAGCGGAAGAACATAAGGTTGGACCATCTGTGGGTCCAGGGCCAAATGAAGA
MF496659.1      ACCGCGCGCAGCGGAGGAACACATACAGGGATCATCTGGAGGGTCAGGGCAAAATGAAGA
MF418028.1      TCCGCGCAATTCGGAAGGGCACAGATCCGCGCAATCGGAGGCGCCAGGGCAAAATGAAGA
MH282867.1      TCCGCGCAATTCGGAAGGGCACAGATCTGCGCAATCGGAGGCGCCAGGGCAAAATGAAGA
MK563994.1      TCCGCGCAATTCGGAAGGGCACAGATCTGCGCAATCGGAGGCGCCAGGGCAAAATGAAGA
JQ647830.1      TCCGCGCAATTCGGAAGGGCACAGATTCGCGCAATCGGAGGCGCCAGAGCAAAATGAAGA
MN095414.1      TCCGCGCAATTCGGAAGGGCACAGATCCGCGCAATCGGAGGCGCCAGGGCAAAATGAAGA
KU058657.1      TCCGCGCAATTCGGAAGGGCACAGATCCGCGCAATCGGAGGCGCCAGGGCAAAATGAAGA
KU896946.1      TCCGCGCAATTCGGAAGGGCACAGATCCGCGCAATCGGAGGTGCCAGGGCAAAATGAAGA
MF033144.1      TCCGCGCAATTCGGAAGGGCACAGATCCGCGCAATCGGAGGCGCCAGGGCAAAATGAAGA
MF418030.1      GCCCCGGAATGTAGAGGAGCATAGAGTTGGCCCAAATCAAGGGCACGGGCAGAATGAAGA
MF418031.1      GCCCCGGAATGTAGAGGAGCATAGAGTTGGCCCAAGTCAAGGGCACGGGCAGAATGAAGA
KP089978.1      GCCCCGGCACGTTGAGGAGCATAGAGTTGGCCCAAATCAAGGGCACGGGCAGAATGAAGA
KU896945.1      GCCCAGGAATGTAGAGGAGCATAGAGCTGGCCCAAATCAAGGGCACGGGCAGAATGAAGA
LC375228.1      GCCCAGGAACGTAGAGGAGCATAGAGTTGGCCCAAATCAAGGGCACGGGCAGAATGAAGA
KC430335.1      GCCCCGGAACGTAGAGGAGCATAGAGTTGGCCCAAGTCAAGGGCACGGGCAGAATGAAGA
MF418033.1      GCCCCGGAGTGTAGAGGAGCATAAAGTTGGCCCAAGTCAGGGGCACGGGCAGAATGAAGA
MW331321.1      ACCCCGGAACGTAGGGGAGCATAGAGTTGGCCCAAATCAAGGGCACGGGCAGAATGAAGA
LC375227.1      GCCCAGGAACGTAGAGGAGCATAGAGTTGGCCCAAATCAAGGGCACGGGCAGAATGAAGA
MF375506.1      GCCCCGGAACGTAGAGGAGCATAGAGTTGGCCCAAGTCAAGGGCACGGGCAGAATGAAGA
MK442089.1      GCCCCGGAACGTAGAGGAGCATAGAGTTGGCCCAAGTCAAGGGCACGGGCAGAATGAAGA
AJ863510.1      ACCGCG---CGCAGAAGGGCACATGTATGCGCAACCAGAAGGGCCAGGGCAAAACGAGGA
D00461.1        ACCGCG---CGCAGAAGGGCACATGTATGCGCAACCAGAAGGGCCAGGGCAAAACGAGGA
                 ** **    *  ** * *** ***   *  * *    ******  ***** ********

MF418029.1      AGCTATGCTAGAGCAAAGGCTTATCAGGCTGATTGAGCTTATGGCTGCAAAGAGGCACAA
KR152654.1      AGCAATGCTAGAGCAGCGGCTAATCAGACTAATTGGACTTATGGCTGCAAAGAGGCACAA
MF418035.1      AGCAATGCTAGAGCAGAGGTTAATCAGACTAATTGAGCTTATGGCTGCAAAGAGGCACAA
MF418032.1      AGCTATGTTAGAGCAAAGACTCGTCAGATTGATTGAGCTCATGGCCGCGAAGAGGCATAA
MF496659.1      GGCCTTGTTGGAGCAAAGGCTCATTAGATTGATCGAGCTCATGGCTGCAAAGAGGCATAA
MF418028.1      AGCCATGCTGGAACAAAGGCTCGTTCGATTGATTGAGCTTATGGCCACAAAGAGGCACAA
MH282867.1      AGCCATGCTGGAACAAAGGCTCGTTCGATTGATTGAGCTTATGGCCACAAAGAGGCACAA
MK563994.1      AGCCATGCTGGAACAAAGGCTCGTTCGATTGATTGAGCTTATGGCCACAAAGAGGCACAA
JQ647830.1      AGCCATGCTGGAACAAAGGCTTGTTAGATTGATTGAGCTTATGGCCAAAAAGAGGCACAA
MN095414.1      AGCCATGCTGGAACAAAGGCTCGTTCGATTGATTGAGCTTATGGCCAAAAAGAGGCACAA
KU058657.1      AGCCATGCTGGAACAAAGGCTCGTTCGATTGATTGAGCTTATGGCCAAGAAGAGGCACAA
KU896946.1      AGCCATGCTGGAACAAAGGCTCGTTCGATTGATTGAGCTTATGGCCAAAAAGAGGCACAA
MF033144.1      AGCCATGCTGGAACAAAGGCTCGTTCGATTGATTGAGCTTATGGCCAAAAAGAGGCACAA
MF418030.1      GGCTATGCTGGAGCAGAGGCTCATCAGGTTGATTGAACTCATGGCCTCAAAAAGGCACAA
MF418031.1      GGCTATGCTGGAGCAGAGGCTCATCAGGTTGATTGAACTCATGGCCTCAAAAAGGCACAA
KP089978.1      GGCTATGCTGGAGCAGAGGCTCATCAGGTTGATTGAACTCATGGCCTCGAAGAGGCACAA
KU896945.1      GGCTATGCTGGAGCAGAGGCTCATCAGATTGATTGAACTCATGGCCTCGAAAAGGCACAA
LC375228.1      GGCTATGCTGGAGCAGAGGCTCATCGGATTGATTGGACTCATGGCCTCGAAAAGGCACAA
KC430335.1      GGCTATGCTGGAGCAGAGGCTCATCAGATTGATTGAACTCATGGCCTCGAAAAGGCACAA
MF418033.1      AGCTATGCTGGAGCAGAGGCTCATCAGATTGATTGAACTCATGGCCTCGAAAAGGCACAA
MW331321.1      AGCTATGCTGGAGCAGAGGCTCATCAGACTGATTGAACTCATGGCCTCGAAAAGGCACAA
LC375227.1      GGCTATGCTGGAGCAGAGGCTCATCGGATTGATTGAACTCATGGCCTCGAAAAGGCACAA
MF375506.1      GGCTATGCTGGAGCAGAGGCTCATCAGATTGATTGAACTCATGGCCGCGAGAAGGCACAA
MK442089.1      AGCTATGCTGGAGCAGAGGCTCATCAGATTGATTGAACTCATGGCCTCGAAAAGGCACAA
AJ863510.1      AGCCATGCTGGAGCAAAGACTCATCAGGTTGATTGAGCTCATGGCTACGAAGAGGCACAA
D00461.1        AGCCATGCTGGAGCAAAGACTCATCAGGTTGATTGAGCTCATGGCTACGAAGAGGCACAA
                *** *********** ******************** ** ****** * ** ********

MF418029.1      TTCGACATTGAGCAATATCTCCTTCGAGATAGGAAGACCTGCGCTTGAGCCAACCCCTGA
KR152654.1      TTCAACACTGAGCAATATCTCCTTCGAGATAGGAAGACCTGCGCTCGAGCCAACTCCTGA
MF418035.1      TTCAACACTGAGCAATATCTCCTTCGAGATAGGGAGACCTGCACTCGAGCCGACTCCTGA
MF418032.1      CTCAACGTTGAGCAACATCTCCTTTGAGATAGGTAGGCCCGCTCTTGAGCCCACCCCTGA
MF496659.1      TTCAACATTGAGCAACATCTCATTTGAGATTGGCAGGCCCGCTCTGGAGCCTACGCCCGA
MF418028.1      CTCGACTTTGAGTAACATCTCCTTTGAGATAGGTAGGCCCAGTCTGGAACCGACACCCGA
MH282867.1      CTCAACATTGAGTAACATCTCCTTTGAGATAGGTAGGCCCAGTCTGGAACCAACACCCGA
MK563994.1      CTCGACATTGAGTAACATCTCCTTTGAGATAGGTAGGCCCAGTCTGGAACCAACACCCGA
JQ647830.1      CTCGACATTGAGTAACATCTCCTTTGAGATAGGTAGGCCCAGTTTGGAACCAACGCCCGA
MN095414.1      CTCGACGTTGAGTAACATCTCCTTTGAGATAGGTAGGCCCAGTTTGGAACCAACACCCGA
KU058657.1      CTCGACATTGAGTAACATCTCCTTTGAGATAGGTAGGCCCAGTTTGGAACCAACACCCGA
KU896946.1      CTCGACATTGAGTAACATCTCCTTTGAGATAGGTAGGCCCAGTTTGGAACCAACACCCGA
MF033144.1      CTCTACATTGAGTAACATCTCCTTTGAGATAGGTAGGCCCAGTTTGGAACCAACACCCGA
MF418030.1      TTCAACATTGAGCAACATCTCTTTTGAGATAGGTAGGCCCTCACTTGAGCCGACCCCTGA
MF418031.1      TTCAACATTGAGCAACATCTCTTTTGAGATTGGTAGGCCCTCACTTGAGCCGACCCCTGA
KP089978.1      TTCAACATTGAGCAACATCTCTTTTGAGATAGGTAGGCCCTCGCTTGAGCCGACCCCTGA
KU896945.1      TTCAACATTGAGCAACATCTCTTTTGAGATAGGTAGGCCCTCACTTGAGCCGACCCCTGA
LC375228.1      TTCAACATTGAGCAACATCTCTTTCGAGATAGGTAGGCCCTCGCTTGAGCCGACCCCTGA
KC430335.1      TTCAACATTGAGCAACATCTCTTTTGAGATAGGTCGGCCCTCGCTTGAGCCGACCCATGA
MF418033.1      TTCAACATTGAGCAACATCTCTTTTGAGATAGGTCGGCCCTCACTTGAGCCGACCCCTGA
MW331321.1      TTCAACATTGAGCAATATATCTTTTGAGATAGGTAGGCCCTCACTTGAGCCGACCCCTGA
LC375227.1      TTCAACATTGAGCAACATCTCTTTCGAGATAGGTAGGCCCTCGCTTGAGCCGACCCCTGA
MF375506.1      TTCAACATTGAGCAACATCTCTTTTGAGATAGGTAGGCCCTCGCTTGAGCCGACCCCTGA
MK442089.1      TTCAACATTGAGCAACATCTCTTTTGAGATAGGTAGGCCCTCGCTTGAGCCGACCCCTGA
AJ863510.1      CTCGACATTGAGCAACATCTCCTTTGAAATAGGTAGGCCCAGTCTAGAACCGACCCCTGA
D00461.1        CTCGACATTGAGCAACATCTCCTTTGAAATAGGTAGGCCCAGTCTAGAACCGACCCCTGA
                 ******************** ******************   ** ** ** ** *****
```

```
MF418029.1      GATGAGGAGGAACCCAGAGAACCCATACTCGCGATTTTCAATCGACGAGTTGTTCAAGAT
KR152654.1      GATGAGGAGGAACCCAGAGAACCCATACTCGCGATTTTCAATCGATGAGTTGTTCAAGAT
MF418035.1      GATGAGGAGGAACCCAGAGAACCCATACTCACGATTTTCAATCGATGAGTTGTTCAAGAT
MF418032.1      GATGAGGAGGAATCCGGAAAATCCCTACTCACGATTCTCGATCGATGAGCTGTTCAAAAT
MF496659.1      GATGAGGAGGAACCCGGAGAACCCATACTCGCGATTTTCGATCGACGAGTTGTTCAAGAT
MF418028.1      GATGAGGAGGAATCCGGAAAATCCATACTCTCGATTCTCAATTGACGAGCTGTTCAAAAT
MH282867.1      GATGAGAAGGAATCCGGAAAATCCATACTCTCGATTCTCAATTGACGAGCTGTTCAAAAT
MK563994.1      GATGAGAAGGAATCCGGAAAATCCATACTCTCGATTCTCAATTGACGAGCTGTTCAAAAT
JQ647830.1      GATGAGAAGGAATCCGGAAAACCCATACTCTCGATTTTCAATCGACGAGCTGTTCAAAAT
MN095414.1      GATGAGAAGGAATCCGGAAAATCCATACTCTCGATTCTCAATTGACGAGCTGTTCAAAAT
KU058657.1      GATGAGAAGGAATCCGGAAAATCCATACTCTCGATTCTCAATTGACGAGCTGTTCAAAAT
KU896946.1      GATGAGAAGGAATCCGGAAAATCCATACTCTCGATTCTCAATTGACGAGCTGTTCAAAAT
MF033144.1      GATGAGAAGGAATCCGGAAAATCCATACTCTCGATTCTCAATTGACGAGCTGTTCAAAAT
MF418030.1      AATGCGGAGGAATCCGGAGAACCCGTACTCGCGATTTTCAATCGATGAGCTGTTCAAGAT
MF418031.1      AATGCGGAGGAATCCGGAGAACCCGTACTCGCGGTTTTCAATCGATGAGCTGTTCAAGAT
KP089978.1      AATGCGAAGGAATCCGGAGAATCCATACTCGCGGTTTTCAATCGATGAGCTGTTCAAGAT
KU896945.1      AATGCGGAGGAATCCGGAGAACCCATACTCGCGGTTTTCAATAGATGAGCTGTTCAAGAT
LC375228.1      AATGCGGAGGAATCCGGAGAACCCATACTCGCGGTTTTCAATAGATGAGCTGTTCAAAAT
KC430335.1      AATGCGGAGGAATCCGGAGAACCCATACTCGCGGTTTTCAATCGATGAGCTGTTTAAGAT
MF418033.1      AATGCGGAGGAATCCAGAGAACCCATACTCGCGGTTTTCAATCGATGAGCTGTTCAAGAT
MW331321.1      AATGCGGAGGAATCCGGAGAACCCATACTCGCGTTTTTCAATCGACGAGCTGTTCAAGAT
LC375227.1      AATGCGGAGGAATCCGGAGAACCCATACTCGCGGTTTTCAATAGATGAGCTGTTCAAAAT
MF375506.1      AATGCGGAGGAATCCGGAGAACCCATACTCGCGGTTTTCAATCGATGAGCTGTTCAAGAT
MK442089.1      AATGCGGAGGAATCCGGAGAATCCATACTCGCGGTTTTCAATCGATGAGCTGTTCAAGAT
AJ863510.1      GATGAGGAGGAATCCGGAAAATCCGTACTCTCGGTTCTCAATCGACGAGCTGTTCAAGAT
D00461.1        GATGAGGAGGAATCCGGAAAATCCGTACTCTCGGTTCTCAATCGACGAGCTGTTCAAGAT
                 *** ************* ** ******** ** ** ***** ** *********** **

MF418029.1      GGAAATTCGGTCAGTGTCCAATAATATGGCGAATACTGAGCAGATGGCTCAAATTACTGC
KR152654.1      GGAGATCCGGTCAGTGTCCAATAACATGGCAAATACTGAGCAGATGGCTCAGATCACTGC
MF418035.1      GGAGATCCGATCAGTGTCCAATAACATGGCGAATACTGAGCAGATGGCTCAAATCACTTC
MF418032.1      GGAAATCCGGTCGGTGTCCAACAACATGGCGAATACTGAGCAGATGGCACAGATCACCGC
MF496659.1      GGATATCCGATCGGTGTCCAATAACATGGCGAACACTGAGCAAATGGCTCAGATCACGGC
MF418028.1      GGAAATCCGGTCGGTGTCGAACAACATGGCCAACACTGAACAGATGGCACAGATCACTGC
MH282867.1      GGAAATCCGGTCGGTGTCGAACAACATGGCCAATACTGAACAGATGGCACAGATCACTGC
MK563994.1      GGAAATCCGGTCGGTGTCGAACAACATGGCCAATACTGAACAGATGGCACAGATCACTGC
JQ647830.1      GGAAATCCGGTCGGTGTCGAACAACATGGCCAACACTGAACAAATGGCACAGATCACTGC
MN095414.1      GGAAATCCGGTCGGTGTCGAACAACATGGCCAATACTGAACAAATGGCACAGATCACTGC
KU058657.1      GGAAATCCGGTCGGTGTCGAACAACATGGCCAATACTGAACAAATGGCACAGATCACTGC
KU896946.1      GGAAATCCGGTCGGTGTCGAACAACATGGCCAATACTGAACAAATGGCACAGATCACTGC
MF033144.1      GGAAATCCGGTCGGTGTCGAACAACATGGCCAATACTGAACAAATGGCACAGATCACTGC
MF418030.1      GGAAATCCGATCTGTGTCCAACAACATGGCGAACACCGAGCAAATGGCACAAATCACTGC
MF418031.1      GGAAATCCGATCTGTGTCCAACAACATGGCGAACACCGAGCAAATGGCACAAATCACTGC
KP089978.1      GGAAATCCGGTCTGTGTCCAACAACATGGCGAACACCGAGCAAATGGCACAAATCACTGC
KU896945.1      GGAAATCCGATCTGTGTCCAACAACATGGCGAACACCGAGCAAATGGCACAAATCACTGC
LC375228.1      GGAAATCCGATCTGTGTCCAACAACATGGCGAACACCGAGCAAATGGCACAAATCACTGC
KC430335.1      GGAAATCCGATCTGTGTCCAACAACATGGCGAACACCGAGCAAATGGCACAAATCACTGC
MF418033.1      GGAAATCCGATCTGTGTCCAACAACATGGCGAACACCGAGCAAATGGCACAGATCACTGC
MW331321.1      GGAAATCCGATCTGTGTCCAACAACATGGCGAACACTGAGCAAATGGCACAAATCACTGC
LC375227.1      GGAAATCCGATCTGTGTCCAACAACATGGCGAACACCGAGCAAATGGCACAAATCACTGC
MF375506.1      GGAAATCCGATCAGTGTCCAACAACATGGCGAACACTGAGCAAATGGCACAAATCACTGC
MK442089.1      GGAAATCCGATCTGTGTCCAACAACATGGCGAACACTGAGCAAATGGCACAAATCACTGC
AJ863510.1      GGAAATCCGATCGGTCTCTAACAATATGGCCAACACTGAGCAGATGGCACAGATCACCGC
D00461.1        GGAAATCCGATCGGTCTCTAACAATATGGCCAACACTGAGCAGATGGCACAGATCACCGC
                ********* ** ***** *********** ******************** ********

MF418029.1      TGACATTGCCGGACTTGGCGTTCCCACTGAGCACGTTGCTGGAGTTATACTGAAGGTAGT
KR152654.1      TGACATTGCAGGACTTGGTGTCCCTACTGAGCATGTTGCTGGAGTCATACTGAAGGTGGT
MF418035.1      TGACATTGCAGGACTTGGAGTTCCTACAGAGCATGTTGCCGGAGTCATATTGAAGGTGGT
MF418032.1      AGATATCGCAGGGCTCGGTGTTCCCACTGAGCATGTTGCAGGAGTCATACTGAAGGTGGT
MF496659.1      GGATATCGCTGGTCTCGGTGTTCCCACCGAGCATGTTGCAGGAGTCATATTGAAGGTAGT
MF418028.1      AGATATTGCCGGGCTCGGTGTTCCCACTGAGCATGTTGCTGGCGTCATACTGAAGGTCGT
MH282867.1      AGATATTGCTGGGCTCGGTGTCCCCACTGAGCATGTTGCTGGCGTCATACTGAAGGTCGT
MK563994.1      AGACATTGCTGGGCTCGGTGTTCCCACTGAGCATGTTGCTGGCGTCATACTGAAGGTCGT
JQ647830.1      AGATATTGCCGGGCTCGGTGTTCCCACTGAGCATGTTGCTGGCGTCATACTGAAGGTCGT
MN095414.1      AGATATTGCCGGGCTCGGTGTTCCCACTGAGCATGTTGCTGGCGTCATACTGAAGGTCGT
KU058657.1      AGATATTGCCGGGCTCGGTGTTCCCACTGAGCATGTTGCTGGCGTCATACTGAAGGTCGT
KU896946.1      AGATATCGCCGGGCTCGGTGTTCCCACTGAGCATGTTGCTGGCGTCATACTGAAGGTCGT
MF033144.1      AGATATTGCCGGGCTCGGTGTTCCCACTGAGCATATTGCTGGCGTCATACTGAAGGTCGT
MF418030.1      TGACATCGCTGGACTTGGGGTCCCCACTGAACACGTTGCAGGTGTCATACTGAAAGTGGT
MF418031.1      GGACATCGCTGGACTTGGGGTCCCCACTGAACACGTTGCAGGTGTCATACTGAAAGTGGT
KP089978.1      TGACATCGCTGGACTTGGGGTCCCCACTGAACACGTTGCAGGGGTCATACTGAAAGTGGT
KU896945.1      TGACATCGCTGGACTTGGGGTCCCCACTGAACATGTTGCAGGGGTCATACTGAAAGTGGT
LC375228.1      TGACATCGCTGGACTTGGGGTACCCACTGAACATGTTGCAGGGGTCATACTGAAAGTGGT
KC430335.1      TGACATCGCTGGACTCGGGGTCCCCACTGAACACGTTGCAGGGGTCATACTGAAAGTGGT
MF418033.1      TGACATCGCTGGACTCGGGGTTCCCACTGAACACGTTGCAGGGGTCATACTGAAAGTGGT
MW331321.1      TGACATCGCTGGGCTTGGGGTCCCCACTGAACATGTTGCAGGAGTTATACTGAAAGTGGT
LC375227.1      TGACATCGCTGGACTTGGGGTACCCACTGAACATGTTGCAGGGGTCATACTGAAAGTGGT
MF375506.1      TGACATCGCTGGACTTGGGGTCCCCACTGAACACGTTGCAGGGGTCATACTGAAAGTTGT
MK442089.1      TGATATCGCTGGGCTTGGGGTCCCCACTGAACACGTTGCAGGGGTAATACTGAAAGTGGT
AJ863510.1      GGATATTGCAGGGCTCGGCGTCCCCACAGAGCATGTGGCGGGGGTTATACTGAAGGTCGT
D00461.1        GGATATTGCAGGGCTCGGCGTCCCCACAGAGCATGTGGCGGGGGTTATACTGAAGGTCGT
                 ** ** ** ** ** ** ** ******** ** ***** ** ** ******** ** **
```

```
MF418029.1      GATCATGTGTGCAAGCGTGAGCAGTTCTGTGTATCTGGATCCAGCTGGAACCGTTGAATT
KR152654.1      GATTATGTGTGCTAGCGTGAGCAGCTCGGTGTATCTGGATCCAGCTGGAACCGTTGAATT
MF418035.1      GATTATGTGTGCAAGCGTGAGCAGCTCGGTGTATCTGGATCCAGCTGGAACCGTTGAGTT
MF418032.1      GATCATGTGCGCGAGTGTGAGTAGCTCTGTTTACCTAGACCCTGCTGGAACTGTTGAGTT
MF496659.1      GATAATGTGCGCAAGTGTGAGCAGTTCTGTCTACTTGGATCCCGCAGGAACCGTTGAGTT
MF418028.1      GATCATGTGCGCGAGCGTGAGCAGTTCTGTCTACCTAGACCCTGCAGGAACTGTTGAGTT
MH282867.1      GATTATGTGTGCGAGCGTGAGCAGTTCTGTCTACCTAGACCCTGCAGGAACTGTTGAGTT
MK563994.1      GATTATGTGTGCGAGCGTGAGCAGTTCTGTCTACCTAGACCCTGCAGGAACTGTTGAGTT
JQ647830.1      AATCATGTGCGCAAGCGTGAGTAGTTCTGTCTACCTAGACCCTGCAGGAACTGTTGAGTT
MN095414.1      GATCATGTGCGCAAGCGTAAGCAGTTCTGTCTACCTAGACCCTGCAGGAACTGTTGAGTT
KU058657.1      GATCATGTGCGCAAGCGTGAGCAGTTCTGTCTACCTAGACCCTGCAGGGACTGTTGAATT
KU896946.1      GATCATGTGCGCAAGCGTGAGCAGTTCTGTCTACCTAGACCCTGCAGGGACTGTTGAGTT
MF033144.1      GATCATGTGCGCAAGCGTGAGCAGTTCTGTCTACCTAGACCCTGCAGGAACTGTTGAGTT
MF418030.1      GATCATGTGTGCAAGCGTGAGTAGCTCTGTTTATCTAGATCCAGCAGGGACTGTGGAGTT
MF418031.1      GATCATGTGTGCAAGCGTGAGTAGCTCTGTTTATCTAGATCCAGCAGGGACTGTGGAGTT
KP089978.1      GATCATGTGTGCAAGCGTGAGTAGCTCCGTTTATCTAGATCCAGCGGGGACTGTAGAGTT
KU896945.1      GATCATGTGTGCAAGCGTGAGTAGCTCAGTTTATCTAGATCCAGCAGGGACTGTGGAGTT
LC375228.1      GATCATGTGTGCAAGCGTGAGTAGTTCAGTTTATCTAGATCCAGCAGGGACTGTGGAGTT
KC430335.1      GATCATGTGTGCAAGCGTGAGTAGCTCTGTTTATCTGGACCCGGCAGGGACTGTGGAGTT
MF418033.1      GATCATGTGTGCAAGCGTGAGTAGCTCTGTTTATCTAGACCCGGCAGGGACTGTGGAGTT
MW331321.1      GATCATGTGTGCAAGTGTGAGTAGCTCTGTTTATCTAGATCCAGCAGGGACTGTGGAGTT
LC375227.1      GATCATGTGTGCAAGCGTGAGTAGTTCAGTTTATCTAGATCCAGCAGGGACTGTGGAGTT
MF375506.1      GATCATGTGTGCAAGTGTGAGTAGCTCTGTTTATCTAGATCCAGCTGGGACTGTGGAGTT
MK442089.1      GATCATGTGTGCAAGCGTGAGTAGCTCTGTTTATCTAGATCCAGCAGGGACTGTGGAGTT
AJ863510.1      AATTATGTGCGCAAGCGTGAGCAGTTCTGTCTACTTAGACCCTGCAGGGACTGTTGAGTT
D00461.1        AATTATGTGCGCAAGCGTGAGCAGTTCTGTCTACTTAGACCCTGCAGGGACTGTTGAGTT
                ********* *********** ** ***** ** ***** ** ***** ***** *****

MF418029.1      CCCCACTGGTGCAGTTCCCTTAGATTCCATCATCGCAATCATGAAAAATCGCGCGGGGCT
KR152654.1      TCCCACTGGTGCAGTTCCCTTGGATTCCATCATTGCAATCATGAAAAATCGCGCGGGGCT
MF418035.1      CCCTACCGGTGCAGTTCCCTTAGATTCCATCATTGCTATCATGAAAAATCGCGCGGGGCT
MF418032.1      CCCTTCCGGGGCGGTCCCCTTGGATTCAATAATTGCCATTATGAAAAATCGAGCAGGGTT
MF496659.1      CTCTTCTGGGGCAGTCCCCCTGGATTCCATTATTGCGATCATGAAGAATCGCGCAGGGTT
MF418028.1      CCCCACTGGAGCAGTCCCTTTGGATTCCATAATTGCAATCATGAAAAATCGTGCTGGACT
MH282867.1      CCCCACTGGCGCAGTCCCTTTGGATTCCATAATTGCAATCATGAAAAATCGTGCTGGACT
MK563994.1      CTCCACTGGCGCAGTCCCTTTGGATTCCATAATTGCAATCATGAAAAATCGTGCTGGACT
JQ647830.1      CCCCACTGGAGCAGTCCCTTTGGATTCCATAATTGCAATCATGAAAAATCGTGCTGGACT
MN095414.1      CCCCACTGGAGCAGTCCCTTTGGACTCCATAATTGCAATCATGAAAAATCGTGCTGGACT
KU058657.1      CCCCACTGGAGCAGTCCCTTTGGACTCCATAATTGCAATCATGAAAAATCGTGCTGGACT
KU896946.1      CCCCACTGGAGCGGTCCCTTTGGACTCCATAATTGCAATCATGAAAAATCGTGCTGGACT
MF033144.1      CCCCACTGGAGCAGTCCCTTTGGACTCCATAATTGCAATCATGAAAAATCGTGCTGGACT
MF418030.1      CCCAACAGGCGCAGTGCCCTTGGACTCGATCATTGCAATCATGAAGAATCGCGCGGGATT
MF418031.1      CCCAACAGGCGCAGTGCCCTTGGACTCGATCATTGCAATCATGAAGAATCGCGCGGGATT
KP089978.1      CCCAACTGGCGCAGTGCCTTTGGACTCGATCATCGCAATTATGAAGAATCGCGCGGGTTT
KU896945.1      CCCAACAGGCGCAGTGCCCTTGGACTCGATCATTGCAATTATGAAGAATCGCGCGGGATT
LC375228.1      CCCAACAGGCGCAGTGCCCTTGGACTCGATCATTGCAATTATGAAGAATCGCGCGGGATT
KC430335.1      CCCAACAGGCGCAGTGCCCCTGGACTCGATCATTGCAATCATGAAGAATCGCGCAGGATT
MF418033.1      CTCAACAGGCGCAGTGCCCTTGGACTCGATCATTGCAATCATGAAGAATCGCGCGGGATT
MW331321.1      CCCAACAGGCGCAGTGCCCTTAGACTCGATCATTGCAATTATGAAGAATCGCGCGGGATT
LC375227.1      CCCAACAGGCGCAGTGCCCTTGGACTCGATCATTGCAATTATGAAGAATCGCGCGGGATT
MF375506.1      CCCAACAGGCGCAGTGCCCTTGGACTCGATCATTGCAATTATGAAGAATCGCGCGGGATT
MK442089.1      CCCAACAGGCGCAGTGCCCTTGGACTCAATCATTGCAATTATGAAGAATCGCGCGGGATT
AJ863510.1      CCCTACTGGCGCAGTGCCACTGGATTCCATAATCGCAATCATGAAAAACCGTGCTGGGTT
D00461.1        CCCTACTGGCGCAGTGCCACTGGATTCCATAATCGCAATCATGAAAAACCGTGCTGGGTT
                *** ** ******** ** ***** ** ** ******** ***** ** ** ** *** *

MF418029.1      GAGGAAGGTGTGTAGGTTATATGCTCCAGTCGTTTGGAATTACATGCTCGTACAGAACAG
KR152654.1      AAGGAAGGTGTGTAGGTTGTATGCTCCAGTCGTCTGGAATTACATGCTCGTTCAAAATAG
MF418035.1      AAGGAAGGTGTGTAGGTTGTATGCTCCAGTCGTCTGGAATTACATGCTCGTTCAAAATAG
MF418032.1      GAGGAAGGTGTGTAGGTTGTATGCTCCAGTTGTTTGGAACTACATGCTTGTTCAGAACAG
MF496659.1      GAGGAAGGTGTGTAGGTTGTATGCTCCAGTTGTTTGGAACTATATGCTTGTCCAGAATAG
MF418028.1      GAGGAAGGTGTGCAGGCTGTATGCTCCGGTCGTCTGGAATTACATGCTCGTTCAGAACAG
MH282867.1      GAGGAAGGTGTGCAGGCTGTATGCTCCGGTCGTCTGGAATTACATGCTCGTTCAGAACAG
MK563994.1      GAGGAAGGTGTGCAGGCTGTATGCTCCGGTCGTCTGGAATTACATGCTCGTTCAGAACAG
JQ647830.1      GAGGAAAGTGCGCAGGCTGTATGCCCCGGTCGTCTGGAATTACATGCTCGTTCAGAACAG
MN095414.1      GAGGAAGGTGTGCAGGCTGTATGCCCCGGTCGTCTGGAATTACATGCTCGTTCAGAACAG
KU058657.1      GAGGAAGGTGTGCAGGCTGTATGCCCCGGTCGTCTGGAATTACATGCTCGTTCAGAACAG
KU896946.1      GAGGAAGGTGTGCAGGCTGTATGCCCCGGTCGTCTGGAATTACATGCTCGTTCAGAACAG
MF033144.1      GAGGAAGGTGTGCAGGCTGTATGCCCCGGTCGTCTGGAATTACATGCTCGTTCAGAACAG
MF418030.1      GAGAAAAGTGTGCAGGCTGTATGCTCCAGTCGTGTGGAATTACATGCTAGTCCAGAATAG
MF418031.1      GAGAAAAGTGTGCAGGCTGTATGCTCCAGTCGTGTGGAATTACATGCTAGTCCAGAATAG
KP089978.1      GAGAAAAGTGTGCAGGCTGTATGCTCCAGTTGTGTGGAATTACATGCTAGTCCAGAATAG
KU896945.1      GAGGAAAGTGTGCAGGCTGTATGCTCCAGTCGTGTGGAATTACATGCTAGTCCAGAATAG
LC375228.1      GAGGAAAGTGTGCAGGCTGTATGCTCCAGTTGTGTGGAATTACATGCTAGTCCAGAATAG
KC430335.1      GAGAAAAGTGTGCAGGCTGTACGCTCCAGTCGTGTGGAATTACATGCTAGTACAGAATAG
MF418033.1      GAGAAAAGTGTGCAGGTTGTACGCTCCAGTCGTGTGGAATTACATGCTAGTCCAGAATAG
MW331321.1      GAGAAAAGTGTGCAGGCTGTATGCCCCAGTCGTGTGGAATTACATGCTAGTCCAGAATAG
LC375227.1      GAGGAAAGTGTGCAGGCTGTATGCTCCAGTCGTGTGGAATTACATGCTAGTCCAGAATAG
MF375506.1      GAGAAAAGTGTGCAGGCTGTATGCTCCAGTTGTGTGGAATTACATGCTAGTCCAGAATAG
MK442089.1      GAGAAAAGTGTGCAGGCTGTATGCTCCAGTCGTGTGGAATTACATGCTAGTCCAGAATAG
AJ863510.1      GAGGAAGGTGTGTAGGTTGTATGCTCCGGTCGTTTGGAATTATATGCTTGTTCAGAATAG
D00461.1        GAGGAAGGTGTGTAGGTTGTATGCTCCGGTCGTTTGGAATTATATGCTTGTTCAGAATAG
                *** ** ******************** ***** ************** ** ***** **
```

```
MF418029.1      GCCACCGTCAGACTGGCAGGCTATGGGCTTTCAATGGGGTGCTCGCTTTGCCGCTTTCGA
KR152654.1      GCCACCATCAGACTGGCAGGCTATGGGTTTTCAATGGAACGCTCGCTTCGCCGCTTTTGA
MF418035.1      GCCACCATCAGACTGGCAGGCTATGGGTTTCCAATGGAACGCTCGCTTCGCCGCTTTTGA
MF418032.1      ACCCCCGTCAGACTGGCAAGCCATGGGGTTTCAATGGAATGCACGTTTCGCCGCTTTTGA
MF496659.1      GCCCCCTTCTGATTGGCAGGCCATGGGGTTTCAATGGAATGCGCGTTTCGCCGCTTTTGA
MF418028.1      GCCCCCTTCGGATTGGCAGGCAATGGGGTTTCAATGGAATGCACGCTTCGCCGCTTTTGA
MH282867.1      GCCCCCTTCGGATTGGCAGGCAATGGGGTTTCAATGGAATGCACGCTTCGCCGCTTTTGA
MK563994.1      GCCCCCTTCGGATTGGCAGGCAATGGGGTTTCAATGGAATGCACGCTTCGCCGCTTTTGA
JQ647830.1      GCCTCCATCGGATTGGCAGGCAATGGGGTTTCAATGGAATGCACGTTTCGCCGCTTTTGA
MN095414.1      GCCTCCTTCGGACTGGCAGGCAATGGGGTTTCAATGGAATGCACGCTTTGCCGCTTTTGA
KU058657.1      GCCTCCTTCGGACTGGCAGGCAATGGGGTTTCAATGGAATGCACGCTTCGCCGCTTTTGA
KU896946.1      GCCTCCTTCGGACTGGCAGGCAATGGGGTTTCAATGGAATGCACGCTTCGCCGCTTTTGA
MF033144.1      GCCTCCTTCGGACTGGCAGGCAATGGGGTTTCAATGGAATGCACGCTTCGCCGCTTTTGA
MF418030.1      GCCACCTTCGGATTGGCAGGCCATGGGATTTCAGTGGAACGCACGCTTCGCCGCATTTGA
MF418031.1      GCCACCTTCGGATTGGCAGGCAATGGGATTTCAGTGGAACGCACGCTTCGCCGCATTCGA
KP089978.1      GCCACCTTCGGATTGGCAGGCCATGGGATTCCAATGGAATGCACGCTTCGCCGCATTCGA
KU896945.1      GCCACCTTCGGATTGGCAGGCTATGGGATTCCAATGGAATGCACGCTTCGCCGCATTTGA
LC375228.1      GCCACCTTCGGATTGGCAGGCTATGGGATTCCAGTGGAATGCACGTTTCGCCGCATTTGA
KC430335.1      GCCACCTTCGGATTGGCAGGCCATGGGATTTCAATGGAATGCACGCTTCACCGCATTTGA
MF418033.1      GCCACCTTCGGATTGGCAGGCCATGGGATTTCAATGGAATGCACGCTTCGCCGCATTTGA
MW331321.1      GCCACCTTCGGATTGGCAGGCCATGGGATTTCAATGGAATGCACGCTTCGCCGCATTTGA
LC375227.1      GCCACCTTCGGATTGGCAGGCTATGGGATTCCAGTGGAATGCACGTTTCGCCGCATTTGA
MF375506.1      GCCACCTTCAGATTGGCAGGCCATGGGATTCCAATGGAATGCACGCTTCGCCGCATTTGA
MK442089.1      GCCACCTTCGGATTGGCAGGCCATGGGATTCCAGTGGAACGCACGCTTCGCCGCATTTGA
AJ863510.1      GCCACCTTCAGATTGGCAGGCCATGGGGTTTCAATGGAATGCACGTTTCGCCGCTTTTGA
D00461.1        GCCACCTTCAGATTGGCAGGCCATGGGGTTTCAATGGAATGCACGTTTCGCCGCTTTTGA
                *** ***************** ***** ** ** ***** ************** *****

MF418029.1      CACATTTGATTATGTGACAAATGGCGCAGCAATTCAACCGGTCGAAGGGCTTATCCGAAG
KR152654.1      CACATTTGATTATGTGACTAATGGCGCGGCTATTCAACCGGTCGAGGGGCTGATCCGAAG
MF418035.1      CACATTTGATTATGTGACCAACGGCGCGGCTATTCAACCGGTCGAGGGGCTGATCCGAAG
MF418032.1      CACATTTGACTATGTGACTAACGGTGCAGCAATCCAGCCTGTAGAAGGGCTCATTCGTAG
MF496659.1      CACATTTGATTATGTGACTAATGGTGCCGCGATCCAACCCGTAGAGGGGCTCATTCGTAG
MF418028.1      CACATTTGATTATGTGACTAACGGCGCCGCGATCCAGCCAGTCGAGGGGCTGATCCGTAG
MH282867.1      CACATTTGATTATGTGACAAACGGCGCCGCGATCCAGCCAGTCGAGGGGCTGATCCGTAG
MK563994.1      CACATTTGATTATGTGACTAACGGCGCCGCGATCCAGCCAGTCGAGGGGCTGATCCGTAG
JQ647830.1      CACATTTGATTATGTGACTAACGGCGCCGCGATCCAGCCTGTCGAGGGGCTGATCCGTAG
MN095414.1      CACATTTGATTATGTGACTAACGGCGCCGCGATCCAGCCTGTCGAGGGGCTAATCCGTAG
KU058657.1      CACATTTGATTATGTGACCAACGGCGCCGCGATCCAGCCTGTCGAGGGGCTAATCCGTAG
KU896946.1      CACATTTGATTATGTGACTAACGGCGCCGCGATCCAGCCTGTCGAGGGGCTAATCCGTAG
MF033144.1      CACATTTGATTATGTGACTAACGGCGCCGCGATCCAGCCTGTCGAGGGGCTAATCCGTAG
MF418030.1      CACATTCGATTATGTGACTAATGGGGCTGCAGTCCAACCCGTAGAGGGGCTCATACGAAG
MF418031.1      CACATTCGATTATGTGACTAATGGGGCTGCAGTCCAACCCGTAGAGGGGCTCATACGCAG
KP089978.1      CACATTCGATTATGTGACCAATGAGGCTGCGATCCAGCCCGTAGAGGGGCTCATACGCAG
KU896945.1      CACATTCGATTATGTGACTAATGGGGCTGCGATCCAGCCCGTAGAGGGGCTCATACGTAG
LC375228.1      CACATTCGATTATGTGACTAATGGGGCTGCAATCCAGCCCGTAGAGGGGCTCATACGTAG
KC430335.1      CACATTCGATTATGTGACTAATGGGGCTGCAATCCAGCCCGTAGAGGGGCTCATACGCAG
MF418033.1      CACATTCGATTATGTGACTAATGGGGCTGCAATCCAGCCCGTAGAGGGGCTCATACGCAG
MW331321.1      CACATTCGATTATGTGACTAATGAGGCTGCAATCCAGCCCGTAGAGGGGCTCATACGCAG
LC375227.1      CACATTCGATTATGTGACTAATGGGGCTGCAATCCAGCCCGTAGAGGGGCTCATACGTAG
MF375506.1      CACATTTGATTATGTGACCAATGGGGCTGCAGTTCAGCCCGTAGAGGGGCTCATACGCAG
MK442089.1      CACATTCGATTATGTGACTAATGGGGCTGCAATTCAGCCCGTAGAGGGGCTCATACGCAG
AJ863510.1      CACATTTGATTATGTGACTAACGGCGCTGCAATCCAGCCTGTTGAGGGGCTCATCCGTAG
D00461.1        CACATTTGATTATGTGACTAACGGCGCTGCAATCCAGCCTGTTGAGGGGCTCATCCGTAG
                ****** ************** ** ** ** ***** ** ******** ** ** **

MF418029.1      GCCCACACCCGAGGAAACAATAGCTCACAACGCTCACAAAAGCATGGCCATTGATAAGTC
KR152654.1      ACCCACCCCTGAGGAAACTATAGCTCACAACGCTCACAAGAGCATGGCAATTGATAAGTC
MF418035.1      ACCCACTCCTGAGGAAACTATAGCTCACAACGCTCACAAGAGCATGGCAATTGATAAGTC
MF418032.1      ACCAACTCCTGAGGAGACAATAGCTCATAATGCGCACAAGAGCATGGCGCTTGATAAGTC
MF496659.1      GCCAACTCCTGAGGAGACGATAGCCCATAATGCGCATAAGAGCATGGCGATTGATAAGTC
MF418028.1      GCCCACGCCTGAGGAGACGATAGCTCATAACGCTCATAAGAGCATGGCAATTGATAAGTC
MH282867.1      GCCTACGCCTGAAGAGACGATAGCTCATAACGCTCACAAGAGCATGGCAATTGACAAGTC
MK563994.1      GCCCACGCCTGAGGAGACGATAGCTCATAACGCTCATAAGAGCATGGCAATTGACAAGTC
JQ647830.1      GCCTACGCCTGAGGAGACGATAGCTCATAACGCTCACAAGAGCATGGCAATTGACAAGTC
MN095414.1      GCCCACGCCTGAGGAGACGGTAGCTCATAACGCTCACAAGAGCATGGCAATTGATAAGTC
KU058657.1      GCCCACGCCTGAGGAGACGATAGCTCATAACGCTCACAAGAGCATGGCAATTGATAAGTC
KU896946.1      GCCCACGCCTGAGGAGACGATAGCTCATAACGCTCACAAGAGCATGGCAATTGATAAGTC
MF033144.1      GCCCACGCCTGAGGAGACGATAGCTCATAACGCTCACAAGAGCATGGCAATTGATAAGTC
MF418030.1      ACCCACACCTGAGGAAACAATAGCTCACAATGCCCACAAGAGTATGGCAATTGACAAGTC
MF418031.1      ACCTACACCAGAGGAAACAATAGCTCACAATGCCCACAAGAGTATGGCAATTGACAAGTC
KP089978.1      GCCCACACCTGAGGAAACAATAGCCCACAATGCACACAAGAGTATGGCAATTGACAAGTC
KU896945.1      GCCCACGCCTGAGGAAACTATAGCTCACAATGCCCACAAGAGTATGGCAATTGACAAGTC
LC375228.1      GCCCACGCCTGAGGAAACAATAGCTCACAATGCCCACAAGAGTATGGCAATTGACAAGTC
KC430335.1      GCCCACGCCTGAGGAAACAATAGCTCACAATGCCCACAAGAGTATGGCAATTGACAAGTC
MF418033.1      GCCCACACCTGAGGAAACAATAGCTCACAATGCCCACAAGAGTATGGCGATTGATAAGTC
MW331321.1      GCCCACACCTGAGGAAACCATAGCTCACAATGCCCACAAGAGTATGGCAATTGACAAGTC
LC375227.1      GCCCACGCCTGAGGAAACAATAGCTCACAATGCCCACAAGAGTATGGCAATTGACAAGTC
MF375506.1      GCCCACACCTGAGGAAACAATAGCTCACAATGCCCACAAGAGTATGGCAATTGACAAGTC
MK442089.1      GCCCACACCTGAGGAAACAATAGCTCACAATGCCCACAAGAGTATGGCGATTGACAAGTC
AJ863510.1      GCCGACGCCTGAAGAGACAATAGCTCATAACGCTCACAAGAGCATGGCTATTGATAAGTC
D00461.1        GCCGACGCCTGAAGAGACAATAGCTCATAACGCTCACAAGAGCATGGCTATTGATAAGTC
                 ***** ******** ** ******** ** ** ******** ***** ***** *****
```

```
MF418029.1      GAATAGAAATGAGCGGCTGGCCAACACGAATGTTGAATACACTGGAGGTATGCTTGGTGC
KR152654.1      CAATAGGAATGAGCGACTGGCCAACACAAATGTTGAATACACTGGAGGTATGCTCGGTGC
MF418035.1      CAATAGGAATGAGCGGCTGGCCAACACAAATGTTGAGTACACTGGAGGTATGCTTGGTGC
MF418032.1      AAACAGAAATGAAAGGCTGGTCAACACTAATGTTGAGTACACTGGGGGCATGCTTGGCGC
MF496659.1      GAACAGAAATGAAAGACTCGCCAACACTAATGTTGAGTACACTGGGGGCATGCTTGGCGC
MF418028.1      GAACAGAAATGAAAGGCTGGCCAACACCAACGTTGAATACACCGGGGGCATGCTCGGTGC
MH282867.1      GAACAGAAATGAAAGGCTGGCCAACACCAACGTTGAATACACCGGGGGAATGCTAGGCGC
MK563994.1      GAACAGAAATGAAAGGCTGGCCAACACCAACGTTGAATACACCGGGGGCATGCTCGGTGC
JQ647830.1      CAACAGAAATGAAAGGCTGGCCAACACCAACGTTGAATACACTGGGGGCATGCTCGGCGC
MN095414.1      TAACAGAAATGAAAGGCTGGCTAACACCAACGTTGAATACACCGGGGGCATGCTCGGCGC
KU058657.1      TAACAGAAATGAAAGGCTGGCCAACACCAACGTTGAATACACCGGGGGCATGCTCGGCGC
KU896946.1      TAACAGAAATGAAAGGCTGGCCAACACCAACGTTGAATACACCGGGGGCATGCTCGGCGC
MF033144.1      TAACAGAAATGAAAGGCTGGCCAACACCAACGTTGAATACACCGGGGGCATGCTCGGCGC
MF418030.1      GAACAGAAATGAGCGATTGGCCAACACTAATGTTGAGTACACTGGAGGGATGCTTGGCGC
MF418031.1      GAACAGAAATGAGCGATTGGCCAACACTAATGTTGAGTACACTGGAGGGATGCTTGGCGC
KP089978.1      GAACAGAAATGAGCGATTGGCCAACACTAATGTTGAGTACACTGGAGGGATGCTTGGCGC
KU896945.1      GAACAGAAATGAGCGATTGGCCAACACTAATGTTGAGTACACTGGAGGGATGCTTGGCGC
LC375228.1      GAACAGAAATGAGCGGTTGGCCAACACTAATGTTGAGTACACTGGAGGCATGCTTGGCGC
KC430335.1      GAACAGAAATGAGCGATTGGCCAACACTAATGTTGAGTACACTGGAGGGATGCTTGGCGC
MF418033.1      GAACAGAAATGAGCGATTGGCCAACACCAATGTTGAGTACACTGGAGGGATGCTTGGCGC
MW331321.1      GAACAGAAATGAGCGATTGGCCAACACTAATGTTGAGTACACTGGAGGGATGCTTGGCGC
LC375227.1      GAACAGAAATGAGCGGTTGGCCAACACTAATGTTGAGTACACTGGAGGCATGCTTGGCGC
MF375506.1      GAACAGAAATGAGCGATTGGCCAACACTAATGTTGAGTACACTGGAGGGATGCTTGGCGC
MK442089.1      GAACAGAAATGAGCGATTGGCCAACACGAATGTTGAGTACACTGGAGGGATGCTTGGCGC
AJ863510.1      GAACAGAAATGAAAGGTTGGCTAACACCAACGTTGAGTATACTGGGGGCATGCTCGGTGC
D00461.1        GAACAGAAATGAAAGGTTGGCTAACACCAACGTTGAGTATACTGGGGGCATGCTCGGTGC
                ************  *  ********** ** ***** ******** ** ***** *****

MF418029.1      TGAGATCGTGCGCAATCATAGGAATGCAATTAACCAATGAAAGCGGAGCGTCTAGGAATG
KR152654.1      TGAGATCGTGCGCAATCACAGGAATGCAAATAACCAATGAAAGCGGAGCGTCTAAGTATG
MF418035.1      CGAGATCGTGCGCAATCATAGGAATGCAAATAACCAATGAAAGCGGAGCGTCTAAGTATG
MF418032.1      TGAGATTGTGCGCAACCATCGGAATGCGATAAACCAATGAAGGCGGAGCGTTTAGAAATG
MF496659.1      TGAGATTGTGCGCAACCATCGAAATGCAATAAACCAATGAAAGCGGAGCGTTTAGAAATG
MF418028.1      AGAGGTCGTGCGTAATCATCGGAATGCAGCAAACCAATGAGAGCGGAACGTCTAAATATG
MH282867.1      AGAGGTCGTGCGTAATCATCGGAATGCAGCAAATCAATGAGAGCGGAACGTCTAAATATG
MK563994.1      AGAGGTCGTGCGTAATCATCGGAATGCAGCAAACCAATGAGAGCGGAACGTCTAAATATG
JQ647830.1      TGAGGTCGTGCGTAACCATCGGAATGCAGCAAATCAATGAGAGCAGAACGTCTAAATATG
MN095414.1      TGAGATCGTGCGTAACCATCGGAATGCAGCAAACCAATGAGAGCGGAACGTCTAAATATG
KU058657.1      TGAGATCGTGCGTAACCATCGGAATGCAGCAAACCAATGAGAGCGGAACGTCTAAATATG
KU896946.1      TGAGATCGTGCGTAACCATCGGAATGCAGCAAACCAATGAGAGCGGAACGTCTAAATATG
MF033144.1      CGAGATCGTGCGTAACCATCGGAATGCAGCAAACCAATGAGAGCGGAACGTCTAAATATG
MF418030.1      TGAGATTGTGCGCAATCACCGCAATGCGATCAACCAATGAAGGCAGACCGTTTAGCCATG
MF418031.1      TGAAATTGTGCGCAATCACCGCAATGCGATCAACCAATGAAGGCAGACCGTTTAGCCATG
KP089978.1      TGAGATTGTGCGCAATCATCGTAATGCGATCAACCAATGAAGGCAGACCGTTTAGCTATG
KU896945.1      TGAGATTGTGCGCAATCACCGTAATGCGATCAACCAATGAAGGCAGACCGTTTAGCCACG
LC375228.1      TGAGATTGTGCGCAATCACCGTAATGCGATCAACCAATGAAGGCAGACCGTTTAGCCACG
KC430335.1      TGAGATTGTGCGCAATCACCGTAATGCGATCAACCAATGAAGGCAGACCGTTTAGCCATG
MF418033.1      CGAGATTGTGCGCAATCACCGTAATGCGATCAACCAATGAAGGCAGACCGTTTAGCCATG
MW331321.1      TGAGATTGTGCGCAATCACCGTAATGCGATCAACCAATGAAGGCAGACCGTTTAGCCATG
LC375227.1      TGAGATTGTGCGCAATCACCGTAATGCGATCAACCAATGAAGGCAGACCGTTTAGCCACG
MF375506.1      TGAGATTGTGCGCAATCACCGCAATGCGATCAACCAATGAAGGCAGACCGTTTAGCCATG
MK442089.1      CGAGATTGTGCGCAATCACCGTAATGCGATCAACCAATGAAGGCAGACCGTTTAGCCATG
AJ863510.1      TGAGATTGTGCGTAATCATCGGAATGCAATAAACCAATGAAAGCGGAACGTTTAGAAATG
D00461.1        TGAGATTGTGCGTAATCATCGGAATGCAATAAACCAATGAAAGCGGAACGTTTAGAAATG
                 ***** ***** ***** ** ***** *  *********  ** ** *** **   * *
```
