# Supplementary material for: Virus Elimination from Naturally Infected Field Cultivars of Potato (Solanum tuberosum) by Transgenic RNA Interference
Source: Int J Mol Sci. 2022 Jul 20;23(14):8020. doi: 10.3390/ijms23148020 (PMC9321115; doi:10.3390/ijms23148020)
Supplement: Supplementary file 1 [file ijms-23-08020-s001.zip › Figure S1 IJMS.pdf]

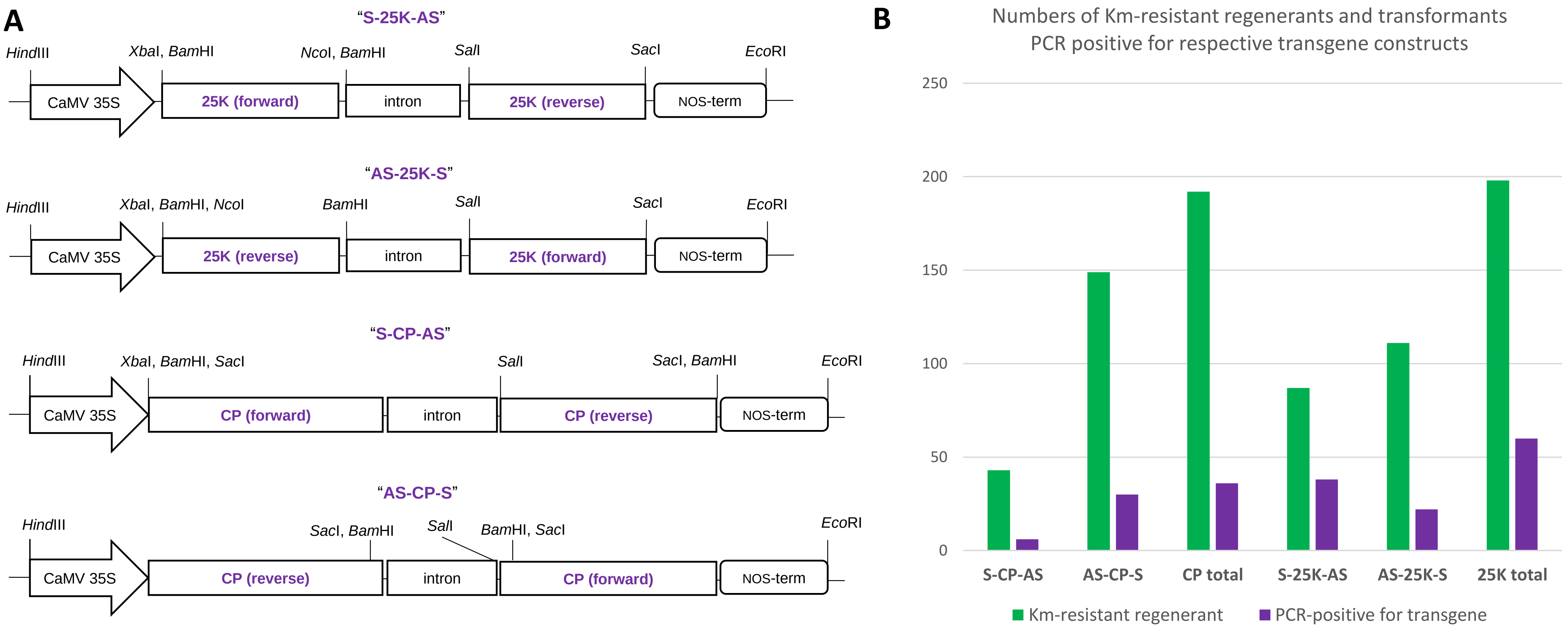

Figure S1. Potato transformation with intron-hairpin RNA constructs. (**A**) Schemes of the intron-hairpin RNA expression cassettes S-CP-AS, AS-CP-S, S-25K-AS and AS-25K-S cloned in the binary vector pCAMBIA2300. (**B**) Potato transformation with the intron-hairpin RNA constructs. Numbers of Km-resistant regenerants and PCR-positive transformant lines were calculated independently for each construct and for two CP constructs together (CP total) and two 25K constructs together (25K total).
