# Supplementary material for: Virus Elimination from Naturally Infected Field Cultivars of Potato (Solanum tuberosum) by Transgenic RNA Interference
Source: Int J Mol Sci. 2022 Jul 20;23(14):8020. doi: 10.3390/ijms23148020 (PMC9321115; doi:10.3390/ijms23148020)
Supplement: Supplementary file 1 [file ijms-23-08020-s001.zip › Table S1 IJMS.pdf]

Table S1. Efficiency of carlavirus elimination from PCR-positive transformant lines of naturally-infected potato cultivars

**Cultivar** (virus infection before transformation)	Line #	Construct used for transformation	Virus detection by ELISA								
			Before planting in the soil			Two months after planting in the soil			Four months after planting in the soil		
			**PVS**	**PVM**	PVY	**PVS**	**PVM**	PVY	**PVS**	**PVM**	PVY
**Albinka** (**PVM** + PVY)	parent	-	-	+	+	-	+	+	-	+	+
	**39**	S-25K-AS	-	+	+	-	-	+	-	-	+
	**43**	S-25K-AS	-	-	+	-	-	+	-	-	+
	**46**	S-25K-AS	-	-	+	-	-	+	-	-	+
	**47**	S-25K-AS	-	-	+	-	-	+	-	-	+
	**48**	S-25K-AS	-	-	+	-	-	+	-	-	+
	**74**	S-25K-AS	-	+	+	-	-	+	-	-	+
	**159**	S-25K-AS	-	-	+	-	-	+	-	-	+
	218	S-25K-AS	-	+	+	-	+	+	-	+	+
**Dunyasha** (**PVM**)	parent	-	-	+	-	-	+	-	-	+	-
	**44**	S-25K-AS	-	-	-	-	-	-	-	-	-
	**61**	S-25K-AS	-	+	-	-	-	-	-	-	-
	62	S-25K-AS	-	+	-	-	+	-	-	+	-
	65	S-25K-AS	-	+	-	-	+	-	-	+	-
	**67**	S-25K-AS	-	+	-	-	-	-	-	-	-
	**146**	AS-CP-S	-	-	-	-	+	-	-	-	-
	162	AS-CP-S	-	+	-	-	-	-	-	+	-
	293	AS-CP-S	-	+	-	-	+	-	-	+	-
	294	AS-CP-S	-	+	-	-	+	-	-	+	-
	312	AS-25K-S	-	+	-	-	+	-	-	+	-
	374	AS-25K-S	-	+	-	-	+	-	-	+	-
**Fortuna** (**PVS** + **PVM**)	parent	-	+	+	-	+	+	-	+	+	-
	309	AS-CP-S	+	+	-	+	+	-	+	+	-
	**360**	AS-CP-S	-	+	-	-	+	-	-	+	-
	361	AS-CP-S	+	+	-	+	+	-	+	+	-
**Kormilitsa** (**PVS**)	parent	-	+	-	-	+	-	-	+	-	-
	**103**	S-25K-AS	+	-	-	-	-	-	-	-	-
**Orbita** (**PVM**)	parent	-	-	+	-	-	+	-	-	+	-
	148	AS-CP-S	-	+	-	-	+	-	-	+	-
**Picasso** (**PVM** + PVY)	parent	-	-	+	+	-	+	+	-	+	+
	161	AS-CP-S	-	+	-	-	-	-	-	+	-
	172	AS-CP-S	-	-	+	-	-	+	-	+	+
	**192**	AS-CP-S	-	-	+	-	+	+	-	-	+
	**194**	AS-25K-S	-	-	+	-	-	+	-	-	+
	257	AS-CP-S	-	+	-	-	+	-	-	+	-
	351	AS-25K-S	-	+	+	-	+	+	-	+	+
	352	AS-25K-S	-	-	+	-	+	+	-	+	+
	381	AS-25K-S	-	+	+	-	+	+	-	+	+
	**383**	AS-25K-S	-	+	-	-	-	-	-	-	-
**Sante** (**PVM** + PVY)	parent	-	-	+	-	-	+	-	-	+	-
	**291**	AS-CP-S	-	-	-	-	-	-	-	-	-
	295	AS-CP-S	-	+	-	-	+	-	-	+	-
	339	AS-CP-S	-	-	-	-	+	-	-	+	-
**Tokhtar** (**PVM**)	parent	-	-	+	-	-	+	-	-	+	-
	69	S-CP-AS	-	+	-	-	+	-	-	+	-
	82	S-CP-AS	-	+	-	-	+	-	-	+	-
	110	AS-CP-S	-	+	-	-	+	-	-	+	-
	160	AS-CP-S	-	+	-	-	+	-	-	+	-
	179	AS-CP-S	-	+	-	-	+	-	-	+	-
	314	AS-CP-S	-	+	-	-	+	-	*	*	*
	315	AS-CP-S	-	+	-	-	+	-	-	+	-
	316	AS-CP-S	-	+	-	-	+	-	*	*	*
	398	AS-CP-S	-	+	-	-	+	-	*	*	*
**Zeren** (**PVS** + **PVM** + PVY)	parent	-	+	+	+	+	+	+	+	+	+
	**38**	S-25K-AS	+	+	+	+	-	-	+	-	-
	59	S-25K-AS	+	+	+	+	+	+	+	+	+
	**119**	S-25K-AS	-	+	-	-	-	-	-	-	-
	344	AS-25K-S	+	+	-	+	+	-	+	+	-
	**336**	AS-25K-S	-	-	-	-	-	-	-	-	-

* not detected, because plant died, or not enough tissue to sample
- virus negative
+ virus positive
